# Effects of Dexamethasone and Insulin Alone or in Combination on Energy and Protein Metabolism Indicators and Milk Production in Dairy Cows in Early Lactation – A Randomized Controlled Trial

**DOI:** 10.1371/journal.pone.0139276

**Published:** 2015-09-30

**Authors:** Mehrdad Sami, Mehrdad Mohri, Hesam A. Seifi

**Affiliations:** Department of Clinical Sciences, School of Veterinary Medicine, Ferdowsi University of Mashhad, P.O. Box 91775–1793, Mashhad, Iran; INRA, FRANCE

## Abstract

**Objectives:**

This study investigated the effects of dexamethasone and insulin, when administered at 3^rd^ or 10^th^ day of lactation on energy and protein metabolism in dairy cows.

**Materials and Methods:**

Two hundred Holstein cows were enrolled in a randomized controlled clinical trial. The cows were randomly assigned to receive 1 of 4 treatments at 3 or 10 days in milk: control group, 10-mL i.m. injection of sterile water, group insulin, s.c. injection of 100 units of insulin, group dexamethasone, i.m. injection of 20 mg of dexamethasone, group insulin plus dexamethasone, i.m. injection of 20 mg of dexamethasone and 100 units of insulin. The cows randomly assigned to receive the treatments on 3 or 10 days of lactation. Serum samples obtained at the time of enrollment, time of treatment and at 2, 4, 7 and 14 days after intervention. The sera were analyzed for β-hydroxybutyrate (BHBA), nonesterified fatty acids (NEFA), glucose, cholesterol, albumin, urea, and aspartate amino transferase (AST). Data were analyzed using a repeated measures mixed model that accounted for the effects of parity, body condition score, dystocia, retained placenta, metritis and the random effect of cow.

**Results:**

There was no significant interaction of group of treatment and time of intervention (day 3 or 10 post-partum) on serum components. Cows that received insulin or dexamethasone alone or in combination, had lower BHBA 2 days after treatment compared with control cows, whereas concentrations of NEFA, were unaffected suggesting that glucocorticoids lipolytic effects do not appear to be important in healthy cows. AST activities significantly reduced in cows that received dexamethasone with or without insulin at 2 and 4 days after treatment. Albumin and urea concentrations 2 days after treatment were higher for cows that received dexamethasone only or dexamethasone plus insulin compared with control and Ins received cows. There were no treatment effects on test-day milk production, milk fat and protein percentages.

**Conclusions:**

The results suggested that administration of glucocorticoids in early lactation resulted in short-term improvement of metabolism in postpartum dairy cows in biochemical terms.

## Introduction

With initiation of lactation, cows are faced with a sudden and tremendous increase in energy demand. This demand is coupled with a decrease in dry matter intake, which generally starts in the dry period. The rate of increase of feed intake post partum lags behind the demands of lactation, leading to a period of negative energy balance [1,2]. As some degree of negative energy balance (NEB) postpartum is physiologically normal, it is the depth, duration, and timing of it that influences the cow’s health and performance. There are several metabolic adaptations to manage NEB, including mobilization of non-esterified fatty acids (NEFA) from body fat reserves, breakdown of protein and glucose sparing for lactogenesis [3].

Subclinical ketosis (SCK) is a very common condition of postpartum dairy cows due to NEB. It was reported that the average of SCK incidence was 43% (ranging from 26 to 56%) and the peak incidence (28.9%) occurred at 5 days in milk [4]. The consequences associated with subclinical ketosis include increased risk of other diseases, decreased milk production, worse reproductive performance, and higher risk of culling in the first 30 days of lactation [4,5].

When treating cows for negative energy balance, it is essential that the need for glucose be met, and that the ketogenic process in the liver be reduced [1,6]. Of all the potential therapies for ketosis in dairy cows, glucocorticoids with or without insulin probably have been the most extensively evaluated [2]. Insulin is used in the treatment of ketosis, because as an anabolic hormone it acts to preserve nutrients in their storage forms by stimulating glycogenesis, lipogenesis, and glycerol synthesis and by inhibiting gluconeogenesis, glycogenolysis, and lipolysis [7].

Glucocorticoids probably have their effect by stimulating proteolysis and inhibiting glucose use in muscle, thereby providing gluconeogenic precursors and stimulating the rate of gluconeogenesis [8,9].

Unfortunately, very few ideal studies have been conducted and findings in the literature are somewhat inconsistent. It was shown that dexamethasone administration 7 and 11 days post partum increased glucose and insulin concentrations and decreased NEFA concentrations. No change in liver function of cows was seen in the study [10]. However, the sample size of the study was limited. In other experiment, dexamethasone resulted in hyperglycemic and hypoketonemic effect lasting 4 to 6 days in ketotic cows [11].

On the other hand, in a large field study on the effects of a corticosteroid with or without insulin, it was shown that isoflupredone alone or with insulin had no therapeutic and preventive effects on SCK 1 or 2 week after treatment [12].

The objectives of this field study were to evaluate the effects of dexamethasone and insulin, when administered at 3^rd^ or 10^th^ day of lactation on some selected energy and protein metabolism indicators as well as milk production in dairy cows. We particularly investigated the effects of treatments on 2^nd^ and 4^th^ d after intervention, because Seifi et al. evaluated the effect of glucocorticoid with or without insulin at least 1 week after treatment [12] and there was no data on energy metabolism within this critical period of time.

## Materials and Methods

### Ethical statement

This study was approved by the ethics committee of Ferdowsi University of Mashhad, Iran, to allow the treatments and collection of blood samples from the coccygeal vein of the animals. The experiment was conducted in a commercial dairy farm. The owner of the farm gave permission to conduct the study on this site. No other specific permissions were required for performing the experiment.

### Cows, Experimental design and Diets

The experiment was conducted in a large-scale dairy herd of about 2000 lactating Holstein cows, in Tehran province, Iran. The rolling herd average for milk production was about 10,675 kg. Two hundred Holstein cows were enrolled in a randomized controlled clinical trial. Seven cows did not complete the study: 4 cows had health problems (fatty liver, gangrenous mastitis, heart insufficiency and recumbency due to slipping). Three cows were culled due to unknown reasons. Thus 193 cows completed the experiment and were used in the analysis.

A 2×4 randomized factorial design of treatments was used. Cows were blocked by parity and expected calving date. Cows were enrolled approximately 14 days before expected calving date (25 animals in each group, five cows were in first lactation and the remainder were in second lactation or greater in each group). The cows randomly assigned to receive the treatments at 2 different time of intervention. Half of the cows received treatments at day 3 postpartum (early treatment) and at the 2^nd^ half at 10 postpartum (lately treatment). Animals at each time of intervention, were randomly assigned to receive 1 of 4 treatments: 1) group control (Con), 10 mL i.m. injection of sterile water in the left semitendinosus muscle plus a 1 mL s.c. injection of sterile water in the caudal left forelimb at the level of the mid-thorax; 2) group insulin (Ins), 10 mL i.m. injection of sterile water in the left semitendinosus muscle plus a 100 unit s.c. injection of isophane insulin, NPH (1 mL contains 100 I.U.) in the caudal left forelimb at the level of the mid-thorax; 3) group dexamethasone (Dex), 20 mg of dexamethasone (2mg/1mL) i.m. in the left semitendinosus muscle plus a 1 mL s.c. injection of sterile water in the caudal left forelimb at the level of the mid-thorax; and 4) group insulin plus dexamethasone (ID), 20 mg of dexamethasone i.m. in the left semitendinosus muscle plus a 100 unit s.c. injection of isophane insulin, NPH in the caudal left forelimb at the level of the mid-thorax.

Cows were scored for body condition on a scale of 1 to 5, in increments of 0.25 [13], at enrollment, the time of treatments and at last sampling. All scorings were performed by a single evaluator.

Definitions of periparturient health events were based on Duffield et al. [14]. The covariates which, may alter the results of the intervention such as parity group (primiparous and multiparous), BCS category, dystocia, retained placenta, and metritis were considered in the statistical analysis. There was no case of milk fever during this study. Individual milk production was collected for all cows from herd records. Data from the first six month of lactation were used to assess the effect of treatment on test-day milk production and components. In addition, Milk breeding value data were collected from Animal Breeding Center of Iran.

The ingredient and nutritional composition of the diets of dry and lactation period are given in Table 1. The herd used mix loose pens with adjacent outside yards and free-stall facilities with sand bedding. The animals had free access to water throughout the experiment.

**Table 1:** Ingredient and nutritional composition (% DM unless otherwise noted) of diets fed to cows during dry and lactation period.

Item	Far-off	Close-up	Fresh cow
**Ingredient**			
Alfalfa hay	26.74	20.47	19.53
Corn silage	22.79	32.95	18.03
Wheat straw	26.06	3.59	─
Beet sugar pulp, dried	─	─	4.99
Molasses, beet sugar	─	─	1.51
Barley meal	10.20	11.74	10.10
Cottonseed, whole with lint	─	2.45	10.00
Corn grain, ground, dry	─	11.6	12.71
Corn gluten meal, dried	─	─	1.92
Canola meal	6.07	─	─
Soybean meal, solv. 44% CP	─	8.98	8.40
Soybean meal, non-enzymatic brown	─	─	2.06
Soybean seed, whole heated	─	─	2.52
Soybean seed, extruded	─	3.37	─
Sunflower meal	6.20	─	─
Salt	0.30	0.08	0.26
Calcium carbonate	0.45	1.16	0.83
Sodium bicarbonate	─	─	1.44
Sodium bentonite	0.45	─	0.55
Di-calcium phosphate	─	─	0.39
Calcium chloride	─	0.78	─
Magnesium oxide	─	0.21	0.25
Magnesium Sulfate	─	0.91	─
Ammonium chloride	─	0.16	─
Transition minerals	─	1.40	─
Transition vitamins	─	0.71	─
Minerals	0.45	─	1.11
Vitamins	0.30	─	0.55
**Energy and nutrients**			
NE_L_, Mcal/kg	1.36	1.59	1.65
NDF	50.10	37.20	33.10
NDF (forage)	43.70	30.40	19.50
ADF	34.40	24.20	22.00
NFC	30.40	38.90	38.80
Ether extract	2.10	3.20	4.40
Crude protein	11.90	14.10	16.60
CP, RDP	8.70	10.20	10.90
CP, RUP	3.20	3.90	5.70
Ca	0.70	1.10	0.80
P	0.30	0.30	0.40
DCAD, mEq/kg	+190	-74	+347

Abbreviations. NE_L_: net energy for lactation; NDF: neutral detergent fiber; ADF: acid detergent fiber; NFC: non-fiber carbohydrates; CP, RDP: crude protein, rumen degradable protein; CP, RUP: crude protein, rumen un-degradable protein; DCAD: dietary cation and anion difference.

### Blood Samples and Laboratory Analysis

Blood samples were collected via the coccygeal vein into 9-mL evacuated tubes with clot activator, approximately 3 hour after morning feeding at the time of enrollment (approximately 14 days before expected calving date), intervention time (days 3 or 10 post calving), and again 2, 4, 7 and 14 days after intervention. The utmost care was taken to minimize stress during sample collection. The blood samples were chilled on ice packs immediately after collection and within 4 hour were centrifuged at 3000 × g for 15 min. Serum was harvested immediately and frozen at −20°C until delivery to the laboratory for further analysis. The NEFA and BHBA were analyzed with commercial kits based on enzymatic reactions (Randox Laboratories Ltd., Ardmore, UK). Concentrations of glucose, aspartate aminotransferase (AST), albumin, urea and cholesterol were analyzed by using commercially available kits (Parsazmoon, Tehran, Iran). Serum biochemistry analyses were conducted with a biochemical auto-analyzer (Biotecnica, BT 1500, Rome, Italy). Control serum (Randox Laboratories Ltd., Ardmore, UK) was used for controlling measurement accuracy. The intra and inter assay coefficient of variation (CV) for measured variables were: AST 3.06% and 1.38%, glucose 0.6% and 1.6%, cholesterol 0.61% and 1.22%, albumin 4.6% and 7.1%, urea 1% and 1.3%, BHBA 3.78% and 5.25% and NEFA 4.81% and 4.32%, respectively.

### Data Management and Statistical analysis

Data of serum profile and BCS were analyzed as repeated-measures-in-time ANOVA study using PROC MIXED of SAS (version 9.2; SAS Institute, Cary, NC). All outcome variables were screened for normality by visual assessment of the distributions and calculation of kurtosis and skewness. The distributions of cholesterol, albumin and urea were normal, whereas the distributions of BHBA, NEFA, glucose and AST were skewed to the right and were transformed with the natural logarithm to achieve a normal distribution. Because measurement time is unequally spaced, the spatial power covariance structure was used for models.

Data were analyzed as a randomized design with a 2 × 4 factorial arrangement of treatments (time of intervention × treatments). The model for each metabolite contained the effects of time of intervention (3 and 10 days post-partum), treatment (Con, Ins, Dex and ID), parity group, BCS category, time (i.e., sample), and the occurrence of dystocia, retained placenta, and metritis. Cows that had a BCS of ≤ 3 were classified as thin, a BCS of 3.25 or 3.5 as fair, and a BCS of ≥ 3.75 as fat. Parity was classified into 2 groups: primiparous and multiparous. Cow nested within intervention time and treatments was designed as a random effect and was used as the error term to test the effects intervention time and treatment effect.

All variables were offered to each model and then removed in a backward stepwise elimination approach. Interactions between treatment and the significant covariates were tested and included in the final model if significant. The interaction between time (intervention, 2, 4, 7 and 14 days after intervention) and treatment was tested. If there was a significant interaction, data were reanalyzed after stratification by sample time. Because there were 6 samples for each cow, a Bonferroni correction of the probability value was used (*P*<0.0083 = 0.05 divided by 6) when there was stratification on time.

Milk production data were analyzed using repeated-measures ANOVA (PROC MIXED in SAS, SAS Inst. Inc.). Separate models were built for milk weight, milk fat percentage, and milk protein percentage. Variables considered in each model, included treatment, time of intervention, BCS category at enrollment, parity group, subclinical ketosis within 1^st^ month of lactation (subclinical ketosis was considered as a concentration of BHBA equal or more than 1200 μmol/L), and disease (cows that had one or more illnesses after parturition were considered diseased; otherwise, cows were considered healthy).

## Results

In total, 200 animals were enrolled. Seven cows died or were sold after enrolment. There was no association of treatment assignment with parity group (*χ2*, *P* = 0.98). Cows were scored for body condition at enrollment, the time of treatments and at last sampling. There was no significant difference of BCS category (≤3.0, 3.25 to 3.5, or ≥3.75) among group of treatments (*P* = 0.26) and additionally, no significant interaction of treatment with time of scoring (*P* = 0.49) on BCS category (Table 2). There were no significant differences of milk breeding value among treatment groups (*P* = 0.39).

**Table 2:** Parity, BCS, dystocia, incidence of retained placenta and metritis and milk breeding value of cows that received placebo (group Con), insulin only (group Ins), dexamethasone only (group Dex), or dexamethasone plus insulin (group ID), once in day 3 or 10 of lactation.

Item	Total	Group Con (n = 49)	Group Ins (n = 47)	Group Dex (n = 49)	Group ID (n = 48)
Parity					
Primiparous	40	10	10	10	10
Multiparous	153	39	37	39	38
BCS at enrollment					
Thin (≤3)	33	6	9	8	10
Fair (3.25 to 3.5)	95	26	21	21	17
Fat (≥3.75)	75	17	17	20	21
Dystocia	52	12	14	16	10
Retained Placenta	27	8	6	7	6
Metritis	24	7	6	6	5
Milk Breeding Value					
Mean		277	372	404	217
SD		677	560	612	510
Minimum		-2133	-1387	-806	-528
Maximum		1302	1597	1903	1665

**Fig 1:**
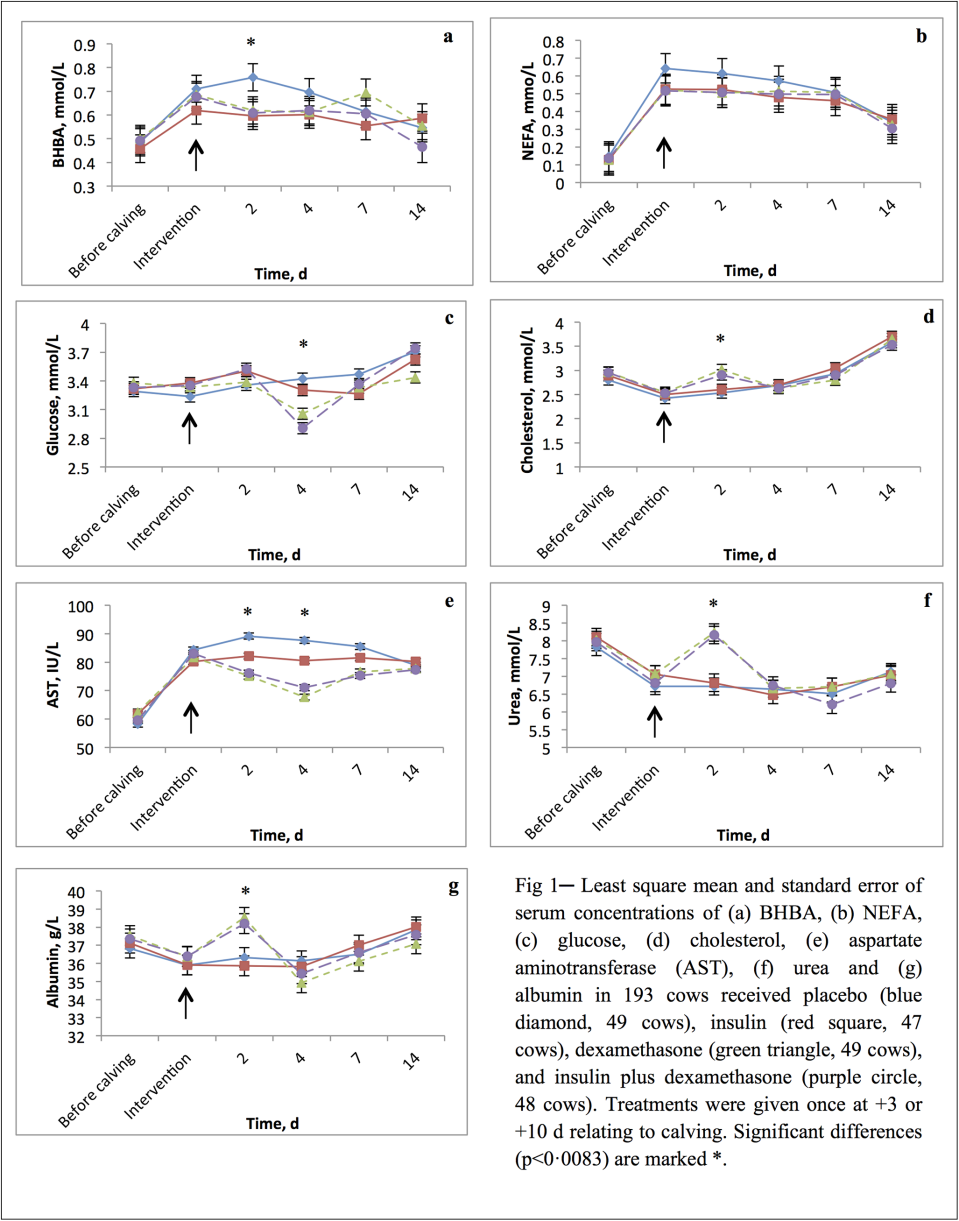
Least square mean and standard error of serum concentrations of (a) BHBA, (b) NEFA, (c) glucose, (d) cholesterol, (e) aspartate aminotransferase (AST), (f) urea and (g) albumin in 193 cows received placebo (blue diamond, 49 cows), insulin (red square, 47 cows), dexamethasone (green triangle, 49 cows), and insulin plus dexamethasone (purple circle, 48 cows). Treatments were given once at +3 or +10 d relating to calving. Significant differences (*P*<0·0083) are marked *.

Results for serum BHBA, NEFA, glucose, albumin, urea, and cholesterol concentrations and AST activities are presented in Fig 1. Time effect was significant for all metabolite variables. Interaction of time of intervention × treatment was not significant for all metabolites. Treatment × time interactions were significant for serum BHBA, glucose, AST, urea, albumin and cholesterol concentrations. All comparisons of treatment groups with controls were accounted to the effects of time of intervention, parity group, BCS category, dystocia, retained placenta, and metritis. Effects of various interventions on serum BHBA, NEFA, AST, glucose, cholesterol, urea and albumin concentrations are presented in Fig 1.

### BHBA

Concentrations of BHBA in treatment groups of Ins and ID were significantly lower than controls on day 2 after intervention (*P*<0.0083). In addition, serum BHBA concentrations tended to be lower in cows treated by Dex in comparison with Con (*P* = 0.012) on the same time of sampling. The BHBA concentrations were greatest in Dex group in comparison to Con, Ins and ID groups on day 7 after intervention. However, Ins group had significantly lower BHBA concentrations in comparison to Dex group on day 7 (*P* = 0.006).

### NEFA

Considering for the effects of time of intervention, parity group, BCS category, dystocia, retained placenta, and metritis as covariates, no significantly effect of treatment and interaction of treatment × time was observed on NEFA concentrations.

### Glucose

There was a significant difference of serum glucose in treatment groups on day 4 of intervention. Glucose concentrations reduced in cows received ID comparing to Con group (*P* = 0.0026). Parity and metritis were significant (*P*<0.05) covariates for glucose. Serum glucose concentrations were higher in primiparous compared to multiparous cows. Cows affected to metritis had significantly lower glucose concentrations.

### AST

Serum AST activities were significantly lower for treated cows with Dex or ID (*P*<0.0083) in comparison to Con and Ins groups on days 2 and 4 after treatment. Similarly, serum AST concentrations were lower (*P*<0.05) in groups treated by Dex or ID in comparison with those of the Ins on 4 day after intervention. BCS was significant (*P* = 0.002) as covariate for AST. Cows with BCS ≤ 3 had higher serum AST concentrations than fair and fat cows.

### Urea

Serum urea concentrations were significantly (*P*<0.0001) higher in groups, which, treated, by Dex or ID compared to Con and Ins at 2 days after treatments. Metritis was significant (*P*<0.0001), as covariates for urea. Cows with metritis had lower serum urea concentrations.

### Albumin

Serum albumin concentrations were significantly higher (*P*<0.0083) for Dex and ID treated groups in comparison with Con and Ins treated cows 2 days after intervention. Cows with metritis, or BCS ≤ 3.0 had significantly (*P* = 0.01) lower serum albumin concentrations.

### Cholesterol

The interaction of treatment × time was significant for cholesterol. Cholesterol concentrations in Dex (*P*<0.0083) and ID (*P*<0.05) groups were significantly greater than Con and Ins groups on day 2 after treatment.

### Milk production and components

There were no treatment effects on milk yield (*P* = 0.18), fat percentage (*P* = 0.61), or protein percentage (*P* = 0.14; Table 3). There were no treatment × test-day interactions. Parity class was significant as covariate for milk production (*P* = 0.0009) and milk protein percentage (*P*<0.0001). Multiparous cows had greater milk production and milk protein percentage.

**Table 3:** The effects of time of lactation, treatment (group ID, insulin plus dexamethasone; group Ins, insulin only; group Dex, dexamethasone; group Con, placebo), parity, and subclinical ketosis on milk production and milk components by repeated-measures of ANOVA. There was no treatment × time interaction.

	Outcomes								
	Milk Kg			Fat %			Protein %		
Variables	Mean	SD	*P*	Mean	SD	*P*	Mean	SD	*P*
**Treatment**	0.18			0.61			0.14		
Ins	43.4	9.8		3.46	0.83		2.85	0.49	
Dex	42.3	10.8		3.42	0.85		2.83	0.30	
ID	41.7	11.6		3.61	1.38		2.87	0.33	
Con	39.6	9.9		3.61	1.11		2.92	0.37	
**Time of lactation** <0.0001				<0.0001			<0.0001		
1^st^ month	37.0	11.6		3.77	1.27		3.05	0.68	
2^nd^ month	45.8	10.7		3.45	1.30		2.68	0.29	
3^rd^ month	44.6	9.7		3.22	0.99		2.78	0.20	
4^th^ month	42.8	9.7		3.50	0.93		2.88	0.22	
5^th^ month	41.4	9.9		3.60	0.80		2.90	0.24	
6^th^ month	39.1	9.2		3.62	0.88		2.92	0.20	
**Parity**		0.0009		0.10			<0.0001		
Primiparous	37.5	8.5		3.45	1.31		2.76	0.28	
Multiparous	43.0	10.9		3.55	0.99		2.90	0.40	

All underlying data of the study is available as the S1 Table.

## Discussion

Various studies have dealt with relationships among glucocorticoids alone or with insulin on ketosis treatment, but it’s controversial about improving effects on energy balance and preventing of ketosis or sub-clinical ketosis in dairy cows. Some studies reported glucocorticoids caused significant increases in plasma glucose and decreases in NEFA and BHBA in plasma [15,16]. However, a large-scale field-based study suggests that there is no benefit of routine use of corticosteroids at the time of calving, and metabolic state may be impaired with their use [12].

The present study investigated the metabolic effects of glucocorticoids and insulin on newly fresh healthy cows. In this regard, the experiments of Jorritsma et al. [17] and Seifi et al. [12] were similar to this study.

Our study differs from other works on several aspects. Firstly, glucocorticoid and/or insulin were administered once on 3^rd^ or 10^th^ day after parturition, and secondly, energy and protein metabolism indicators were measured several times during first two weeks after treatment.

There was no significant interaction between time of intervention (day 3 or 10 postpartum) and groups of experiment on serum components. It seems there is no difference of time of administration of glucocorticoids and/or insulin on serum metabolites during early lactation of healthy cows. However, remarkable differences on metabolites were shown among treatment groups.

BHBA concentrations were significantly lower for cows treated with Ins, Dex or ID 2 days after treatment. It was shown that treatment with a single dose of isoflupredone acetate with or without insulin within 8 days in milk, not only had no significant positive effects on SCK prevention, even treatment with isoflupredone plus insulin increased the risk of developing and remaining ketotic compared with treatment with isoflupredone alone [12]. Cows that received isoflupredone with or without insulin had significantly higher concentrations of BHBA and NEFA at the first sampling time (1 week) after intervention [12], whereas in our study serum BHBA concentrations were significantly lower in cows treated by Dex or ID and also Ins alone than controls at the first sampling time (2 days) after treatment. The major difference between 2 studies is timing of samplings. We measured the metabolites 48 h after treatment, whereas samples were taken 1 week after treatment in Seifi et al. [12]. Interestingly, our results showed that serum BHBA concentrations were peaking at 1 week after Dex administration, which was similar to changes of BHBA concentrations after isoflupredone administration reported earlier [12].

In short term, Glucocorticoids shift glucose distribution away from the intracellular compartment and into the extracellular and vascular compartments [18]. This results in a reduction in gluconeogenesis [19]. Decreased gluconeogenesis causes an increased Krebs cycle intermediates and concomitant reduction in ketogenesis [20,21].

On the other hand, hypoketonemic effect of Ins therapy may be due to its activity on decrease activity of carnitine palmitoyl transferase I (CPT I) and increase affinity of CPT I for malonyl-CoA [22]. In the liver, this enzyme is a potent regulator of translocation of long-chain fatty acids from the cytoplasm into the mitochondria, where they partition for esterification and oxidation [23]. Moreover, insulin’s inhibitory effect on ketogenesis is also related to its stimulatory effect on activity of acetyl-CoA carboxylase or formation of malonyl-CoA that inhibits activity of CPT I [24].

No treatment effect on serum NEFA concentrations was observed. Injection of a glucocorticoid (isoflupredone) increased serum NEFA concentrations, and increased the risk for subclinical ketosis in healthy early post-partum cows [12]. In other studies, glucocorticoids did not increase or decrease NEFA concentrations [17,25]. It remains to be elucidated whether glucocorticoids increase lipolysis in the adipose tissue of healthy cows, since increased release of NEFA would not be consistent with glucocorticoids treatment. Decrease of BHBA and unaffected concentrations of NEFA in cows that received glucocorticoids, suggesting that glucocorticoids lipolytic effects do not appear to be important in healthy transition cows after single dose treatment.

Glucose concentrations were significantly lower in ID group in comparison to Con and Dex groups on day 4 after treatment. This finding is in agreement with the results of earlier study [12]. They showed glucose concentrations significantly decreased in cows treated by isoflupredone plus insulin, but not isoflupredone alone, both 1 and 2 week after treatment. This difference was attributed to the effect of insulin [12]. However, there was no such effect of Ins alone treatment on glucose concentrations in the present study. The activity of insulin is expected to last no more than 24 hours [26]. It is not clear why treatment of glucocorticoid plus insulin had decreasing effect on glucose on 4 day after therapy in this study or 1 and 2 week after treatment in the study of Seifi et al. [12].

The hyperglycemic effect of glucocorticoids [17,27] was not seen in this study. However, It was reported that the hyperglycemic effect of glucocorticoids peaked 24 hours after injection, and glucose concentration declined afterwards [27]. Therefore, it seems this effect could not be detected in our study because the first sampling was on d 2 after treatment. On the other hand, glucose is an insensitive measure of energy status because it is subject to tight homeostatic regulation [1]. In addition, serum glucose had no strong correlations with other energy-related metabolites [28].

AST activities decreased in Dex and ID treatment groups compared with controls on days 2 and 4 after treatment. The reduction in the activity of AST possibly suggests either a reduction in liver damage or a reduction in muscle breakdown [17]. AST activity evaluation is part of analyzing biochemical parameters in peri-partum dairy cows [28].

AST is a widely distributed enzyme, which is found in many tissues and organs, with high activity in the liver that leaks out into the general circulation when liver cells are injured [29]. Glucocorticoids help maintain cell membrane integrity [30] and interfere with AST leakage. This fact may be an explanation to lower AST activity in Dex received groups.

In the present study treatments of Dex or ID caused an increase in concentrations of serum urea and albumin on day 2 after treatment. This increase may be attributed to glucocorticoids effect, because Ins alone did not exert such an effect.

Albumin is synthesized in the liver and blood albumin concentration reflects amino acid supply into liver and metabolic responses repartitioning available amino acids [31]. Glucocorticoids inhibit protein synthesis and stimulate protein catabolism in the cells [32,33] and enhance amino acid mobilization into liver [20]. Coincidentally with the reduced proteins elsewhere in the body, the liver proteins become enhanced. The increased plasma concentration of amino acids and enhanced transport of amino acids into the hepatic cells by glucocorticoids could also account for enhanced rate of deamination of amino acids by the liver [33], which results in increase the concentration of urea. In addition, increased protein synthesis in the liver, and increased formation of plasma proteins by the liver in Dex received groups, increased serum albumin concentration.

On the other hand, within 48 hours of administration, glucocorticoids will stimulate appetite for 18 to 24 hours [34], which may account to increased urea formation due to enhanced ammonia absorption from rumen [35].

Increase of cholesterol in Dex received groups occurred on day 2 after treatment. The effect of glucocorticoids in inducing hypercholesterolemia is a known effect in humans [36]. Hypercholesterolemia is usually recognized as a secondary hyperlipidemia in animals [37]. The importance of this finding in dairy cows is not clear to the authors.

Despite the numerical increase of milk production in treatment groups than controls, there were no significant effects on milk production or milk components. A short-term depression in milk production due to glucocorticoids was reported [11]. However, this effect was not seen in a longer duration of time [12].

## Conclusions

This study demonstrated that administration of Ins and Dex had beneficial effects on energy metabolism in short term (2 days), which was demonstrated by decreased concentrations of BHBA. In spite of never given insulin as the sole treatment of ketosis, because of the risk of hypoglycemia, Ins received cows showed better energy metabolism than controls. In addition, increased concentrations of albumin and urea and decreased activities of AST in Dex treated cows implicated an improvement of metabolism in postpartum dairy cows. However, all the effects were seen in short term and there was no longer time improvement of NEB or protein metabolism.

The study was limited in measuring feed intake and predicting energy balance, because the experiment was performed in a commercial farm and cows were housed with the main herd. So, measurement of individual cow feed intake was not possible. This study was not designed as a ketosis treatment study, because all fresh animals were enrolled. Therefore, the results should be considered in NEB clinical cases with caution.

## Supporting Information

S1 Table Caption: Underlying data of the study including blood metabolites concentrations, groups and covariates. (PDF)

S2 Table Caption: REFLECT checklist. (DOC)
