# Supplementary material for: Effects of Dexamethasone and Insulin Alone or in Combination on Energy and Protein Metabolism Indicators and Milk Production in Dairy Cows in Early Lactation – A Randomized Controlled Trial
Source: PLoS One. 2015 Sep 30;10(9):e0139276. doi: 10.1371/journal.pone.0139276 (PMC4589323; doi:10.1371/journal.pone.0139276)
Supplement: S1 Table — (PDF) [file pone.0139276.s001.pdf]

Cow	time of intervention	Subgroup	Parity	Parity 2 g	parity 3 g	Time	DIM
28	+3 DIM	Ins	1	1	1	precalving	-14
28	+3 DIM	Ins	1	1	1	Intervention	3
28	+3 DIM	Ins	1	1	1	Int+2	5
28	+3 DIM	Ins	1	1	1	Int+4	7
28	+3 DIM	Ins	1	1	1	Int+7	10
28	+3 DIM	Ins	1	1	1	Int+14	17
47	+3 DIM	Con	4	2	3	precalving	-14
47	+3 DIM	Con	4	2	3	Intervention	3
47	+3 DIM	Con	4	2	3	Int+2	5
47	+3 DIM	Con	4	2	3	Int+4	7
47	+3 DIM	Con	4	2	3	Int+7	10
47	+3 DIM	Con	4	2	3	Int+14	17
56	+3 DIM	Con	1	1	1	precalving	-14
56	+3 DIM	Con	1	1	1	Intervention	3
56	+3 DIM	Con	1	1	1	Int+2	5
56	+3 DIM	Con	1	1	1	Int+4	7
56	+3 DIM	Con	1	1	1	Int+7	10
56	+3 DIM	Con	1	1	1	Int+14	17
67	+3 DIM	Ins	1	1	1	precalving	-14
67	+3 DIM	Ins	1	1	1	Intervention	3
67	+3 DIM	Ins	1	1	1	Int+2	5
67	+3 DIM	Ins	1	1	1	Int+4	7
67	+3 DIM	Ins	1	1	1	Int+7	10
67	+3 DIM	Ins	1	1	1	Int+14	17
71	+3 DIM	Dex	1	1	1	precalving	-14
71	+3 DIM	Dex	1	1	1	Intervention	3
71	+3 DIM	Dex	1	1	1	Int+2	5
71	+3 DIM	Dex	1	1	1	Int+4	7
71	+3 DIM	Dex	1	1	1	Int+7	10
71	+3 DIM	Dex	1	1	1	Int+14	17
78	+3 DIM	Ins	1	1	1	precalving	-14
78	+3 DIM	Ins	1	1	1	Intervention	3
78	+3 DIM	Ins	1	1	1	Int+2	5
78	+3 DIM	Ins	1	1	1	Int+4	7
78	+3 DIM	Ins	1	1	1	Int+7	10
78	+3 DIM	Ins	1	1	1	Int+14	17
94	+3 DIM	Ins+Dex	1	1	1	precalving	-14
94	+3 DIM	Ins+Dex	1	1	1	Intervention	3
94	+3 DIM	Ins+Dex	1	1	1	Int+2	5
94	+3 DIM	Ins+Dex	1	1	1	Int+4	7
94	+3 DIM	Ins+Dex	1	1	1	Int+7	10
94	+3 DIM	Ins+Dex	1	1	1	Int+14	17
95	+10 DIM	Con	1	1	1	precalving	-14
95	+10 DIM	Con	1	1	1	Intervention	10
95	+10 DIM	Con	1	1	1	Int+2	12
95	+10 DIM	Con	1	1	1	Int+4	14
95	+10 DIM	Con	1	1	1	Int+7	17
95	+10 DIM	Con	1	1	1	Int+14	24

Cow	time of intervention	Subgroup	Parity	Parity 2 g	parity 3 g	Time	DIM
99	+10 DIM	Con	1	1	1	precalving	-14
99	+10 DIM	Con	1	1	1	Intervention	10
99	+10 DIM	Con	1	1	1	Int+2	12
99	+10 DIM	Con	1	1	1	Int+4	14
99	+10 DIM	Con	1	1	1	Int+7	17
99	+10 DIM	Con	1	1	1	Int+14	24
102	+3 DIM	Con	3	2	3	precalving	-14
102	+3 DIM	Con	3	2	3	Intervention	3
102	+3 DIM	Con	3	2	3	Int+2	5
102	+3 DIM	Con	3	2	3	Int+4	7
102	+3 DIM	Con	3	2	3	Int+7	10
102	+3 DIM	Con	3	2	3	Int+14	17
106	+10 DIM	Ins	1	1	1	precalving	-14
106	+10 DIM	Ins	1	1	1	Intervention	10
106	+10 DIM	Ins	1	1	1	Int+2	12
106	+10 DIM	Ins	1	1	1	Int+4	14
106	+10 DIM	Ins	1	1	1	Int+7	17
106	+10 DIM	Ins	1	1	1	Int+14	24
107	+3 DIM	Ins	1	1	1	precalving	-14
107	+3 DIM	Ins	1	1	1	Intervention	3
107	+3 DIM	Ins	1	1	1	Int+2	5
107	+3 DIM	Ins	1	1	1	Int+4	7
107	+3 DIM	Ins	1	1	1	Int+7	10
107	+3 DIM	Ins	1	1	1	Int+14	17
110	+10 DIM	Ins+Dex	1	1	1	precalving	-14
110	+10 DIM	Ins+Dex	1	1	1	Intervention	10
110	+10 DIM	Ins+Dex	1	1	1	Int+2	12
110	+10 DIM	Ins+Dex	1	1	1	Int+4	14
110	+10 DIM	Ins+Dex	1	1	1	Int+7	17
110	+10 DIM	Ins+Dex	1	1	1	Int+14	24
112	+10 DIM	Ins	1	1	1	precalving	-14
112	+10 DIM	Ins	1	1	1	Intervention	10
112	+10 DIM	Ins	1	1	1	Int+2	12
112	+10 DIM	Ins	1	1	1	Int+4	14
112	+10 DIM	Ins	1	1	1	Int+7	17
112	+10 DIM	Ins	1	1	1	Int+14	24
118	+3 DIM	Ins+Dex	1	1	1	precalving	-14
118	+3 DIM	Ins+Dex	1	1	1	Intervention	3
118	+3 DIM	Ins+Dex	1	1	1	Int+2	5
118	+3 DIM	Ins+Dex	1	1	1	Int+4	7
118	+3 DIM	Ins+Dex	1	1	1	Int+7	10
118	+3 DIM	Ins+Dex	1	1	1	Int+14	17
120	+10 DIM	Con	1	1	1	precalving	-14
120	+10 DIM	Con	1	1	1	Intervention	10
120	+10 DIM	Con	1	1	1	Int+2	12
120	+10 DIM	Con	1	1	1	Int+4	14
120	+10 DIM	Con	1	1	1	Int+7	17
120	+10 DIM	Con	1	1	1	Int+14	24

Cow	time of intervention	Subgroup	Parity	Parity 2 g	parity 3 g	Time	DIM
132	+10 DIM	Ins	1	1	1	precalving	-14
132	+10 DIM	Ins	1	1	1	Intervention	10
132	+10 DIM	Ins	1	1	1	Int+2	12
132	+10 DIM	Ins	1	1	1	Int+4	14
132	+10 DIM	Ins	1	1	1	Int+7	17
132	+10 DIM	Ins	1	1	1	Int+14	24
147	+10 DIM	Dex	1	1	1	precalving	-14
147	+10 DIM	Dex	1	1	1	Intervention	10
147	+10 DIM	Dex	1	1	1	Int+2	12
147	+10 DIM	Dex	1	1	1	Int+4	14
147	+10 DIM	Dex	1	1	1	Int+7	17
147	+10 DIM	Dex	1	1	1	Int+14	24
151	+10 DIM	Ins+Dex	3	2	3	precalving	-14
151	+10 DIM	Ins+Dex	3	2	3	Intervention	10
151	+10 DIM	Ins+Dex	3	2	3	Int+2	12
151	+10 DIM	Ins+Dex	3	2	3	Int+4	14
151	+10 DIM	Ins+Dex	3	2	3	Int+7	17
151	+10 DIM	Ins+Dex	3	2	3	Int+14	24
186	+3 DIM	Con	1	1	1	precalving	-14
186	+3 DIM	Con	1	1	1	Intervention	3
186	+3 DIM	Con	1	1	1	Int+2	5
186	+3 DIM	Con	1	1	1	Int+4	7
186	+3 DIM	Con	1	1	1	Int+7	10
186	+3 DIM	Con	1	1	1	Int+14	17
188	+3 DIM	Ins	1	1	1	precalving	-14
188	+3 DIM	Ins	1	1	1	Intervention	3
188	+3 DIM	Ins	1	1	1	Int+2	5
188	+3 DIM	Ins	1	1	1	Int+4	7
188	+3 DIM	Ins	1	1	1	Int+7	10
188	+3 DIM	Ins	1	1	1	Int+14	17
190	+10 DIM	Ins+Dex	1	1	1	precalving	-14
190	+10 DIM	Ins+Dex	1	1	1	Intervention	10
190	+10 DIM	Ins+Dex	1	1	1	Int+2	12
190	+10 DIM	Ins+Dex	1	1	1	Int+4	14
190	+10 DIM	Ins+Dex	1	1	1	Int+7	17
190	+10 DIM	Ins+Dex	1	1	1	Int+14	24
208	+3 DIM	Con	1	1	1	precalving	-14
208	+3 DIM	Con	1	1	1	Intervention	3
208	+3 DIM	Con	1	1	1	Int+2	5
208	+3 DIM	Con	1	1	1	Int+4	7
208	+3 DIM	Con	1	1	1	Int+7	10
208	+3 DIM	Con	1	1	1	Int+14	17
224	+3 DIM	Dex	1	1	1	precalving	-14
224	+3 DIM	Dex	1	1	1	Intervention	3
224	+3 DIM	Dex	1	1	1	Int+2	5
224	+3 DIM	Dex	1	1	1	Int+4	7
224	+3 DIM	Dex	1	1	1	Int+7	10
224	+3 DIM	Dex	1	1	1	Int+14	17

Cow	time of intervention	Subgroup	Parity	Parity 2 g	parity 3 g	Time	DIM
225	+10 DIM	Con	1	1	1	precalving	-14
225	+10 DIM	Con	1	1	1	Intervention	10
225	+10 DIM	Con	1	1	1	Int+2	12
225	+10 DIM	Con	1	1	1	Int+4	14
225	+10 DIM	Con	1	1	1	Int+7	17
225	+10 DIM	Con	1	1	1	Int+14	24
231	+3 DIM	Dex	1	1	1	precalving	-14
231	+3 DIM	Dex	1	1	1	Intervention	3
231	+3 DIM	Dex	1	1	1	Int+2	5
231	+3 DIM	Dex	1	1	1	Int+4	7
231	+3 DIM	Dex	1	1	1	Int+7	10
231	+3 DIM	Dex	1	1	1	Int+14	17
245	+3 DIM	Dex	1	1	1	precalving	-14
245	+3 DIM	Dex	1	1	1	Intervention	3
245	+3 DIM	Dex	1	1	1	Int+2	5
245	+3 DIM	Dex	1	1	1	Int+4	7
245	+3 DIM	Dex	1	1	1	Int+7	10
245	+3 DIM	Dex	1	1	1	Int+14	17
247	+10 DIM	Ins	1	1	1	precalving	-14
247	+10 DIM	Ins	1	1	1	Intervention	10
247	+10 DIM	Ins	1	1	1	Int+2	12
247	+10 DIM	Ins	1	1	1	Int+4	14
247	+10 DIM	Ins	1	1	1	Int+7	17
247	+10 DIM	Ins	1	1	1	Int+14	24
248	+10 DIM	Dex	1	1	1	precalving	-14
248	+10 DIM	Dex	1	1	1	Intervention	10
248	+10 DIM	Dex	1	1	1	Int+2	12
248	+10 DIM	Dex	1	1	1	Int+4	14
248	+10 DIM	Dex	1	1	1	Int+7	17
248	+10 DIM	Dex	1	1	1	Int+14	24
249	+10 DIM	Ins	1	1	1	precalving	-14
249	+10 DIM	Ins	1	1	1	Intervention	10
249	+10 DIM	Ins	1	1	1	Int+2	12
249	+10 DIM	Ins	1	1	1	Int+4	14
249	+10 DIM	Ins	1	1	1	Int+7	17
249	+10 DIM	Ins	1	1	1	Int+14	24
256	+10 DIM	Dex	1	1	1	precalving	-14
256	+10 DIM	Dex	1	1	1	Intervention	10
256	+10 DIM	Dex	1	1	1	Int+2	12
256	+10 DIM	Dex	1	1	1	Int+4	14
256	+10 DIM	Dex	1	1	1	Int+7	17
256	+10 DIM	Dex	1	1	1	Int+14	24
261	+3 DIM	Ins+Dex	1	1	1	precalving	-14
261	+3 DIM	Ins+Dex	1	1	1	Intervention	3
261	+3 DIM	Ins+Dex	1	1	1	Int+2	5
261	+3 DIM	Ins+Dex	1	1	1	Int+4	7
261	+3 DIM	Ins+Dex	1	1	1	Int+7	10
261	+3 DIM	Ins+Dex	1	1	1	Int+14	17

Cow	time of intervention	Subgroup	Parity	Parity 2 g	parity 3 g	Time	DIM
329	+3 DIM	Dex	4	2	3	precalving	-14
329	+3 DIM	Dex	4	2	3	Intervention	3
329	+3 DIM	Dex	4	2	3	Int+2	5
329	+3 DIM	Dex	4	2	3	Int+4	7
329	+3 DIM	Dex	4	2	3	Int+7	10
329	+3 DIM	Dex	4	2	3	Int+14	17
337	+3 DIM	Con	4	2	3	precalving	-14
337	+3 DIM	Con	4	2	3	Intervention	3
337	+3 DIM	Con	4	2	3	Int+2	5
337	+3 DIM	Con	4	2	3	Int+4	7
337	+3 DIM	Con	4	2	3	Int+7	10
337	+3 DIM	Con	4	2	3	Int+14	17
524	+10 DIM	Con	5	2	3	precalving	-14
524	+10 DIM	Con	5	2	3	Intervention	10
524	+10 DIM	Con	5	2	3	Int+2	12
524	+10 DIM	Con	5	2	3	Int+4	14
524	+10 DIM	Con	5	2	3	Int+7	17
524	+10 DIM	Con	5	2	3	Int+14	24
662	+10 DIM	Ins	6	2	3	precalving	-14
662	+10 DIM	Ins	6	2	3	Intervention	10
662	+10 DIM	Ins	6	2	3	Int+2	12
662	+10 DIM	Ins	6	2	3	Int+4	14
662	+10 DIM	Ins	6	2	3	Int+7	17
662	+10 DIM	Ins	6	2	3	Int+14	24
731	+3 DIM	Ins	4	2	3	precalving	-14
731	+3 DIM	Ins	4	2	3	Intervention	3
731	+3 DIM	Ins	4	2	3	Int+2	5
731	+3 DIM	Ins	4	2	3	Int+4	7
731	+3 DIM	Ins	4	2	3	Int+7	10
731	+3 DIM	Ins	4	2	3	Int+14	17
813	+3 DIM	Ins+Dex	1	1	1	precalving	-14
813	+3 DIM	Ins+Dex	1	1	1	Intervention	3
813	+3 DIM	Ins+Dex	1	1	1	Int+2	5
813	+3 DIM	Ins+Dex	1	1	1	Int+4	7
813	+3 DIM	Ins+Dex	1	1	1	Int+7	10
813	+3 DIM	Ins+Dex	1	1	1	Int+14	17
856	+10 DIM	Con	4	2	3	precalving	-14
856	+10 DIM	Con	4	2	3	Intervention	10
856	+10 DIM	Con	4	2	3	Int+2	12
856	+10 DIM	Con	4	2	3	Int+4	14
856	+10 DIM	Con	4	2	3	Int+7	17
856	+10 DIM	Con	4	2	3	Int+14	24
938	+3 DIM	Dex	1	1	1	precalving	-14
938	+3 DIM	Dex	1	1	1	Intervention	3
938	+3 DIM	Dex	1	1	1	Int+2	5
938	+3 DIM	Dex	1	1	1	Int+4	7
938	+3 DIM	Dex	1	1	1	Int+7	10
938	+3 DIM	Dex	1	1	1	Int+14	17

Cow	time of intervention	Subgroup	Parity	Parity 2 g	parity 3 g	Time	DIM
944	+3 DIM	Ins+Dex	2	2	2	precalving	-14
944	+3 DIM	Ins+Dex	2	2	2	Intervention	3
944	+3 DIM	Ins+Dex	2	2	2	Int+2	5
944	+3 DIM	Ins+Dex	2	2	2	Int+4	7
944	+3 DIM	Ins+Dex	2	2	2	Int+7	10
944	+3 DIM	Ins+Dex	2	2	2	Int+14	17
1281	+3 DIM	Ins+Dex	7	2	3	precalving	-14
1281	+3 DIM	Ins+Dex	7	2	3	Intervention	3
1281	+3 DIM	Ins+Dex	7	2	3	Int+2	5
1281	+3 DIM	Ins+Dex	7	2	3	Int+4	7
1281	+3 DIM	Ins+Dex	7	2	3	Int+7	10
1281	+3 DIM	Ins+Dex	7	2	3	Int+14	17
2493	+3 DIM	Dex	4	2	3	precalving	-14
2493	+3 DIM	Dex	4	2	3	Intervention	3
2493	+3 DIM	Dex	4	2	3	Int+2	5
2493	+3 DIM	Dex	4	2	3	Int+4	7
2493	+3 DIM	Dex	4	2	3	Int+7	10
2493	+3 DIM	Dex	4	2	3	Int+14	17
2624	+3 DIM	Con	4	2	3	precalving	-14
2624	+3 DIM	Con	4	2	3	Intervention	3
2624	+3 DIM	Con	4	2	3	Int+2	5
2624	+3 DIM	Con	4	2	3	Int+4	7
2624	+3 DIM	Con	4	2	3	Int+7	10
2624	+3 DIM	Con	4	2	3	Int+14	17
2694	+3 DIM	Ins+Dex	3	2	3	precalving	-14
2694	+3 DIM	Ins+Dex	3	2	3	Intervention	3
2694	+3 DIM	Ins+Dex	3	2	3	Int+2	5
2694	+3 DIM	Ins+Dex	3	2	3	Int+4	7
2694	+3 DIM	Ins+Dex	3	2	3	Int+7	10
2694	+3 DIM	Ins+Dex	3	2	3	Int+14	17
2741	+3 DIM	Con	4	2	3	precalving	-14
2741	+3 DIM	Con	4	2	3	Intervention	3
2741	+3 DIM	Con	4	2	3	Int+2	5
2741	+3 DIM	Con	4	2	3	Int+4	7
2741	+3 DIM	Con	4	2	3	Int+7	10
2741	+3 DIM	Con	4	2	3	Int+14	17
2764	+10 DIM	Ins	3	2	3	precalving	-14
2764	+10 DIM	Ins	3	2	3	Intervention	10
2764	+10 DIM	Ins	3	2	3	Int+2	12
2764	+10 DIM	Ins	3	2	3	Int+4	14
2764	+10 DIM	Ins	3	2	3	Int+7	17
2764	+10 DIM	Ins	3	2	3	Int+14	24
2813	+10 DIM	Con	3	2	3	precalving	-14
2813	+10 DIM	Con	3	2	3	Intervention	10
2813	+10 DIM	Con	3	2	3	Int+2	12
2813	+10 DIM	Con	3	2	3	Int+4	14
2813	+10 DIM	Con	3	2	3	Int+7	17
2813	+10 DIM	Con	3	2	3	Int+14	24

Cow	time of intervention	Subgroup	Parity	Parity 2 g	parity 3 g	Time	DIM
2940	+3 DIM	Con	7	2	3	precalving	-14
2940	+3 DIM	Con	7	2	3	Intervention	3
2940	+3 DIM	Con	7	2	3	Int+2	5
2940	+3 DIM	Con	7	2	3	Int+4	7
2940	+3 DIM	Con	7	2	3	Int+7	10
2940	+3 DIM	Con	7	2	3	Int+14	17
3526	+10 DIM	Dex	2	2	2	precalving	-14
3526	+10 DIM	Dex	2	2	2	Intervention	10
3526	+10 DIM	Dex	2	2	2	Int+2	12
3526	+10 DIM	Dex	2	2	2	Int+4	14
3526	+10 DIM	Dex	2	2	2	Int+7	17
3526	+10 DIM	Dex	2	2	2	Int+14	24
4019	+10 DIM	Dex	5	2	3	precalving	-14
4019	+10 DIM	Dex	5	2	3	Intervention	10
4019	+10 DIM	Dex	5	2	3	Int+2	12
4019	+10 DIM	Dex	5	2	3	Int+4	14
4019	+10 DIM	Dex	5	2	3	Int+7	17
4019	+10 DIM	Dex	5	2	3	Int+14	24
4044	+10 DIM	Ins+Dex	4	2	3	precalving	-14
4044	+10 DIM	Ins+Dex	4	2	3	Intervention	10
4044	+10 DIM	Ins+Dex	4	2	3	Int+2	12
4044	+10 DIM	Ins+Dex	4	2	3	Int+4	14
4044	+10 DIM	Ins+Dex	4	2	3	Int+7	17
4044	+10 DIM	Ins+Dex	4	2	3	Int+14	24
4053	+3 DIM	Ins	6	2	3	precalving	-14
4053	+3 DIM	Ins	6	2	3	Intervention	3
4053	+3 DIM	Ins	6	2	3	Int+2	5
4053	+3 DIM	Ins	6	2	3	Int+4	7
4053	+3 DIM	Ins	6	2	3	Int+7	10
4053	+3 DIM	Ins	6	2	3	Int+14	17
5061	+10 DIM	Dex	4	2	3	precalving	-14
5061	+10 DIM	Dex	4	2	3	Intervention	10
5061	+10 DIM	Dex	4	2	3	Int+2	12
5061	+10 DIM	Dex	4	2	3	Int+4	14
5061	+10 DIM	Dex	4	2	3	Int+7	17
5061	+10 DIM	Dex	4	2	3	Int+14	24
5104	+10 DIM	Dex	2	2	2	precalving	-14
5104	+10 DIM	Dex	2	2	2	Intervention	10
5104	+10 DIM	Dex	2	2	2	Int+2	12
5104	+10 DIM	Dex	2	2	2	Int+4	14
5104	+10 DIM	Dex	2	2	2	Int+7	17
5104	+10 DIM	Dex	2	2	2	Int+14	24
5165	+3 DIM	Con	3	2	3	precalving	-14
5165	+3 DIM	Con	3	2	3	Intervention	3
5165	+3 DIM	Con	3	2	3	Int+2	5
5165	+3 DIM	Con	3	2	3	Int+4	7
5165	+3 DIM	Con	3	2	3	Int+7	10
5165	+3 DIM	Con	3	2	3	Int+14	17

Cow	time of intervention	Subgroup	Parity	Parity 2 g	parity 3 g	Time	DIM
5170	+10 DIM	Ins	5	2	3	precalving	-14
5170	+10 DIM	Ins	5	2	3	Intervention	10
5170	+10 DIM	Ins	5	2	3	Int+2	12
5170	+10 DIM	Ins	5	2	3	Int+4	14
5170	+10 DIM	Ins	5	2	3	Int+7	17
5170	+10 DIM	Ins	5	2	3	Int+14	24
5254	+3 DIM	Ins	3	2	3	precalving	-14
5254	+3 DIM	Ins	3	2	3	Intervention	3
5254	+3 DIM	Ins	3	2	3	Int+2	5
5254	+3 DIM	Ins	3	2	3	Int+4	7
5254	+3 DIM	Ins	3	2	3	Int+7	10
5254	+3 DIM	Ins	3	2	3	Int+14	17
5371	+10 DIM	Ins	2	2	2	precalving	-14
5371	+10 DIM	Ins	2	2	2	Intervention	10
5371	+10 DIM	Ins	2	2	2	Int+2	12
5371	+10 DIM	Ins	2	2	2	Int+4	14
5371	+10 DIM	Ins	2	2	2	Int+7	17
5371	+10 DIM	Ins	2	2	2	Int+14	24
5397	+10 DIM	Ins	2	2	2	precalving	-14
5397	+10 DIM	Ins	2	2	2	Intervention	10
5397	+10 DIM	Ins	2	2	2	Int+2	12
5397	+10 DIM	Ins	2	2	2	Int+4	14
5397	+10 DIM	Ins	2	2	2	Int+7	17
5397	+10 DIM	Ins	2	2	2	Int+14	24
5405	+10 DIM	Ins+Dex	2	2	2	precalving	-14
5405	+10 DIM	Ins+Dex	2	2	2	Intervention	10
5405	+10 DIM	Ins+Dex	2	2	2	Int+2	12
5405	+10 DIM	Ins+Dex	2	2	2	Int+4	14
5405	+10 DIM	Ins+Dex	2	2	2	Int+7	17
5405	+10 DIM	Ins+Dex	2	2	2	Int+14	24
5415	+3 DIM	Ins+Dex	2	2	2	precalving	-14
5415	+3 DIM	Ins+Dex	2	2	2	Intervention	3
5415	+3 DIM	Ins+Dex	2	2	2	Int+2	5
5415	+3 DIM	Ins+Dex	2	2	2	Int+4	7
5415	+3 DIM	Ins+Dex	2	2	2	Int+7	10
5415	+3 DIM	Ins+Dex	2	2	2	Int+14	17
5771	+3 DIM	Dex	9	2	3	precalving	-14
5771	+3 DIM	Dex	9	2	3	Intervention	3
5771	+3 DIM	Dex	9	2	3	Int+2	5
5771	+3 DIM	Dex	9	2	3	Int+4	7
5771	+3 DIM	Dex	9	2	3	Int+7	10
5771	+3 DIM	Dex	9	2	3	Int+14	17
5919	+10 DIM	Con	7	2	3	precalving	-14
5919	+10 DIM	Con	7	2	3	Intervention	10
5919	+10 DIM	Con	7	2	3	Int+2	12
5919	+10 DIM	Con	7	2	3	Int+4	14
5919	+10 DIM	Con	7	2	3	Int+7	17
5919	+10 DIM	Con	7	2	3	Int+14	24

Cow	time of intervention	Subgroup	Parity	Parity 2 g	parity 3 g	Time	DIM
5971	+3 DIM	Ins+Dex	6	2	3	precalving	-14
5971	+3 DIM	Ins+Dex	6	2	3	Intervention	3
5971	+3 DIM	Ins+Dex	6	2	3	Int+2	5
5971	+3 DIM	Ins+Dex	6	2	3	Int+4	7
5971	+3 DIM	Ins+Dex	6	2	3	Int+7	10
5971	+3 DIM	Ins+Dex	6	2	3	Int+14	17
6052	+10 DIM	Ins+Dex	5	2	3	precalving	-14
6052	+10 DIM	Ins+Dex	5	2	3	Intervention	10
6052	+10 DIM	Ins+Dex	5	2	3	Int+2	12
6052	+10 DIM	Ins+Dex	5	2	3	Int+4	14
6052	+10 DIM	Ins+Dex	5	2	3	Int+7	17
6052	+10 DIM	Ins+Dex	5	2	3	Int+14	24
6123	+3 DIM	Dex	5	2	3	precalving	-14
6123	+3 DIM	Dex	5	2	3	Intervention	3
6123	+3 DIM	Dex	5	2	3	Int+2	5
6123	+3 DIM	Dex	5	2	3	Int+4	7
6123	+3 DIM	Dex	5	2	3	Int+7	10
6123	+3 DIM	Dex	5	2	3	Int+14	17
6145	+10 DIM	Dex	1	1	1	precalving	-14
6145	+10 DIM	Dex	1	1	1	Intervention	10
6145	+10 DIM	Dex	1	1	1	Int+2	12
6145	+10 DIM	Dex	1	1	1	Int+4	14
6145	+10 DIM	Dex	1	1	1	Int+7	17
6145	+10 DIM	Dex	1	1	1	Int+14	24
6203	+10 DIM	Dex	5	2	3	precalving	-14
6203	+10 DIM	Dex	5	2	3	Intervention	10
6203	+10 DIM	Dex	5	2	3	Int+2	12
6203	+10 DIM	Dex	5	2	3	Int+4	14
6203	+10 DIM	Dex	5	2	3	Int+7	17
6203	+10 DIM	Dex	5	2	3	Int+14	24
6220	+10 DIM	Ins+Dex	5	2	3	precalving	-14
6220	+10 DIM	Ins+Dex	5	2	3	Intervention	10
6220	+10 DIM	Ins+Dex	5	2	3	Int+2	12
6220	+10 DIM	Ins+Dex	5	2	3	Int+4	14
6220	+10 DIM	Ins+Dex	5	2	3	Int+7	17
6220	+10 DIM	Ins+Dex	5	2	3	Int+14	24
6284	+3 DIM	Ins+Dex	4	2	3	precalving	-14
6284	+3 DIM	Ins+Dex	4	2	3	Intervention	3
6284	+3 DIM	Ins+Dex	4	2	3	Int+2	5
6284	+3 DIM	Ins+Dex	4	2	3	Int+4	7
6284	+3 DIM	Ins+Dex	4	2	3	Int+7	10
6284	+3 DIM	Ins+Dex	4	2	3	Int+14	17
6515	+10 DIM	Con	3	2	3	precalving	-14
6515	+10 DIM	Con	3	2	3	Intervention	10
6515	+10 DIM	Con	3	2	3	Int+2	12
6515	+10 DIM	Con	3	2	3	Int+4	14
6515	+10 DIM	Con	3	2	3	Int+7	17
6515	+10 DIM	Con	3	2	3	Int+14	24

Cow	time of intervention	Subgroup	Parity	Parity 2 g	parity 3 g	Time	DIM
6540	+10 DIM	Ins	4	2	3	precalving	-14
6540	+10 DIM	Ins	4	2	3	Intervention	10
6540	+10 DIM	Ins	4	2	3	Int+2	12
6540	+10 DIM	Ins	4	2	3	Int+4	14
6540	+10 DIM	Ins	4	2	3	Int+7	17
6540	+10 DIM	Ins	4	2	3	Int+14	24
6724	+10 DIM	Ins	5	2	3	precalving	-14
6724	+10 DIM	Ins	5	2	3	Intervention	10
6724	+10 DIM	Ins	5	2	3	Int+2	12
6724	+10 DIM	Ins	5	2	3	Int+4	14
6724	+10 DIM	Ins	5	2	3	Int+7	17
6724	+10 DIM	Ins	5	2	3	Int+14	24
7048	+10 DIM	Ins	4	2	3	precalving	-14
7048	+10 DIM	Ins	4	2	3	Intervention	10
7048	+10 DIM	Ins	4	2	3	Int+2	12
7048	+10 DIM	Ins	4	2	3	Int+4	14
7048	+10 DIM	Ins	4	2	3	Int+7	17
7048	+10 DIM	Ins	4	2	3	Int+14	24
7050	+10 DIM	Con	4	2	3	precalving	-14
7050	+10 DIM	Con	4	2	3	Intervention	10
7050	+10 DIM	Con	4	2	3	Int+2	12
7050	+10 DIM	Con	4	2	3	Int+4	14
7050	+10 DIM	Con	4	2	3	Int+7	17
7050	+10 DIM	Con	4	2	3	Int+14	24
7067	+10 DIM	Ins+Dex	3	2	3	precalving	-14
7067	+10 DIM	Ins+Dex	3	2	3	Intervention	10
7067	+10 DIM	Ins+Dex	3	2	3	Int+2	12
7067	+10 DIM	Ins+Dex	3	2	3	Int+4	14
7067	+10 DIM	Ins+Dex	3	2	3	Int+7	17
7067	+10 DIM	Ins+Dex	3	2	3	Int+14	24
7068	+10 DIM	Dex	4	2	3	precalving	-14
7068	+10 DIM	Dex	4	2	3	Intervention	10
7068	+10 DIM	Dex	4	2	3	Int+2	12
7068	+10 DIM	Dex	4	2	3	Int+4	14
7068	+10 DIM	Dex	4	2	3	Int+7	17
7068	+10 DIM	Dex	4	2	3	Int+14	24
7113	+10 DIM	Dex	4	2	3	precalving	-14
7113	+10 DIM	Dex	4	2	3	Intervention	10
7113	+10 DIM	Dex	4	2	3	Int+2	12
7113	+10 DIM	Dex	4	2	3	Int+4	14
7113	+10 DIM	Dex	4	2	3	Int+7	17
7113	+10 DIM	Dex	4	2	3	Int+14	24
7121	+3 DIM	Ins+Dex	1	1	1	precalving	-14
7121	+3 DIM	Ins+Dex	1	1	1	Intervention	3
7121	+3 DIM	Ins+Dex	1	1	1	Int+2	5
7121	+3 DIM	Ins+Dex	1	1	1	Int+4	7
7121	+3 DIM	Ins+Dex	1	1	1	Int+7	10
7121	+3 DIM	Ins+Dex	1	1	1	Int+14	17

Cow	time of intervention	Subgroup	Parity	Parity 2 g	parity 3 g	Time	DIM
7133	+10 DIM	Ins+Dex	4	2	3	precalving	-14
7133	+10 DIM	Ins+Dex	4	2	3	Intervention	10
7133	+10 DIM	Ins+Dex	4	2	3	Int+2	12
7133	+10 DIM	Ins+Dex	4	2	3	Int+4	14
7133	+10 DIM	Ins+Dex	4	2	3	Int+7	17
7133	+10 DIM	Ins+Dex	4	2	3	Int+14	24
7136	+3 DIM	Ins	4	2	3	precalving	-14
7136	+3 DIM	Ins	4	2	3	Intervention	3
7136	+3 DIM	Ins	4	2	3	Int+2	5
7136	+3 DIM	Ins	4	2	3	Int+4	7
7136	+3 DIM	Ins	4	2	3	Int+7	10
7136	+3 DIM	Ins	4	2	3	Int+14	17
7140	+10 DIM	Ins+Dex	4	2	3	precalving	-14
7140	+10 DIM	Ins+Dex	4	2	3	Intervention	10
7140	+10 DIM	Ins+Dex	4	2	3	Int+2	12
7140	+10 DIM	Ins+Dex	4	2	3	Int+4	14
7140	+10 DIM	Ins+Dex	4	2	3	Int+7	17
7140	+10 DIM	Ins+Dex	4	2	3	Int+14	24
7141	+3 DIM	Ins	4	2	3	precalving	-14
7141	+3 DIM	Ins	4	2	3	Intervention	3
7141	+3 DIM	Ins	4	2	3	Int+2	5
7141	+3 DIM	Ins	4	2	3	Int+4	7
7141	+3 DIM	Ins	4	2	3	Int+7	10
7141	+3 DIM	Ins	4	2	3	Int+14	17
7151	+10 DIM	Ins+Dex	1	1	1	precalving	-14
7151	+10 DIM	Ins+Dex	1	1	1	Intervention	10
7151	+10 DIM	Ins+Dex	1	1	1	Int+2	12
7151	+10 DIM	Ins+Dex	1	1	1	Int+4	14
7151	+10 DIM	Ins+Dex	1	1	1	Int+7	17
7151	+10 DIM	Ins+Dex	1	1	1	Int+14	24
7157	+3 DIM	Con	3	2	3	precalving	-14
7157	+3 DIM	Con	3	2	3	Intervention	3
7157	+3 DIM	Con	3	2	3	Int+2	5
7157	+3 DIM	Con	3	2	3	Int+4	7
7157	+3 DIM	Con	3	2	3	Int+7	10
7157	+3 DIM	Con	3	2	3	Int+14	17
7177	+10 DIM	Con	4	2	3	precalving	-14
7177	+10 DIM	Con	4	2	3	Intervention	10
7177	+10 DIM	Con	4	2	3	Int+2	12
7177	+10 DIM	Con	4	2	3	Int+4	14
7177	+10 DIM	Con	4	2	3	Int+7	17
7177	+10 DIM	Con	4	2	3	Int+14	24
7194	+10 DIM	Ins+Dex	4	2	3	precalving	-14
7194	+10 DIM	Ins+Dex	4	2	3	Intervention	10
7194	+10 DIM	Ins+Dex	4	2	3	Int+2	12
7194	+10 DIM	Ins+Dex	4	2	3	Int+4	14
7194	+10 DIM	Ins+Dex	4	2	3	Int+7	17
7194	+10 DIM	Ins+Dex	4	2	3	Int+14	24

Cow	time of intervention	Subgroup	Parity	Parity 2 g	parity 3 g	Time	DIM
7268	+3 DIM	Dex	3	2	3	precalving	-14
7268	+3 DIM	Dex	3	2	3	Intervention	3
7268	+3 DIM	Dex	3	2	3	Int+2	5
7268	+3 DIM	Dex	3	2	3	Int+4	7
7268	+3 DIM	Dex	3	2	3	Int+7	10
7268	+3 DIM	Dex	3	2	3	Int+14	17
7322	+3 DIM	Ins+Dex	3	2	3	precalving	-14
7322	+3 DIM	Ins+Dex	3	2	3	Intervention	3
7322	+3 DIM	Ins+Dex	3	2	3	Int+2	5
7322	+3 DIM	Ins+Dex	3	2	3	Int+4	7
7322	+3 DIM	Ins+Dex	3	2	3	Int+7	10
7322	+3 DIM	Ins+Dex	3	2	3	Int+14	17
7353	+3 DIM	Ins	4	2	3	precalving	-14
7353	+3 DIM	Ins	4	2	3	Intervention	3
7353	+3 DIM	Ins	4	2	3	Int+2	5
7353	+3 DIM	Ins	4	2	3	Int+4	7
7353	+3 DIM	Ins	4	2	3	Int+7	10
7353	+3 DIM	Ins	4	2	3	Int+14	17
7368	+3 DIM	Con	3	2	3	precalving	-14
7368	+3 DIM	Con	3	2	3	Intervention	3
7368	+3 DIM	Con	3	2	3	Int+2	5
7368	+3 DIM	Con	3	2	3	Int+4	7
7368	+3 DIM	Con	3	2	3	Int+7	10
7368	+3 DIM	Con	3	2	3	Int+14	17
7403	+3 DIM	Con	3	2	3	precalving	-14
7403	+3 DIM	Con	3	2	3	Intervention	3
7403	+3 DIM	Con	3	2	3	Int+2	5
7403	+3 DIM	Con	3	2	3	Int+4	7
7403	+3 DIM	Con	3	2	3	Int+7	10
7403	+3 DIM	Con	3	2	3	Int+14	17
7412	+10 DIM	Dex	3	2	3	precalving	-14
7412	+10 DIM	Dex	3	2	3	Intervention	10
7412	+10 DIM	Dex	3	2	3	Int+2	12
7412	+10 DIM	Dex	3	2	3	Int+4	14
7412	+10 DIM	Dex	3	2	3	Int+7	17
7412	+10 DIM	Dex	3	2	3	Int+14	24
7521	+10 DIM	Con	3	2	3	precalving	-14
7521	+10 DIM	Con	3	2	3	Intervention	10
7521	+10 DIM	Con	3	2	3	Int+2	12
7521	+10 DIM	Con	3	2	3	Int+4	14
7521	+10 DIM	Con	3	2	3	Int+7	17
7521	+10 DIM	Con	3	2	3	Int+14	24
7537	+3 DIM	Ins	3	2	3	precalving	-14
7537	+3 DIM	Ins	3	2	3	Intervention	3
7537	+3 DIM	Ins	3	2	3	Int+2	5
7537	+3 DIM	Ins	3	2	3	Int+4	7
7537	+3 DIM	Ins	3	2	3	Int+7	10
7537	+3 DIM	Ins	3	2	3	Int+14	17

Cow	time of intervention	Subgroup	Parity	Parity 2 g	parity 3 g	Time	DIM
7543	+3 DIM	Dex	3	2	3	precalving	-14
7543	+3 DIM	Dex	3	2	3	Intervention	3
7543	+3 DIM	Dex	3	2	3	Int+2	5
7543	+3 DIM	Dex	3	2	3	Int+4	7
7543	+3 DIM	Dex	3	2	3	Int+7	10
7543	+3 DIM	Dex	3	2	3	Int+14	17
7550	+3 DIM	Ins	3	2	3	precalving	-14
7550	+3 DIM	Ins	3	2	3	Intervention	3
7550	+3 DIM	Ins	3	2	3	Int+2	5
7550	+3 DIM	Ins	3	2	3	Int+4	7
7550	+3 DIM	Ins	3	2	3	Int+7	10
7550	+3 DIM	Ins	3	2	3	Int+14	17
7712	+10 DIM	Con	4	2	3	precalving	-14
7712	+10 DIM	Con	4	2	3	Intervention	10
7712	+10 DIM	Con	4	2	3	Int+2	12
7712	+10 DIM	Con	4	2	3	Int+4	14
7712	+10 DIM	Con	4	2	3	Int+7	17
7712	+10 DIM	Con	4	2	3	Int+14	24
8015	+10 DIM	Ins	3	2	3	precalving	-14
8015	+10 DIM	Ins	3	2	3	Intervention	10
8015	+10 DIM	Ins	3	2	3	Int+2	12
8015	+10 DIM	Ins	3	2	3	Int+4	14
8015	+10 DIM	Ins	3	2	3	Int+7	17
8015	+10 DIM	Ins	3	2	3	Int+14	24
8017	+10 DIM	Dex	3	2	3	precalving	-14
8017	+10 DIM	Dex	3	2	3	Intervention	10
8017	+10 DIM	Dex	3	2	3	Int+2	12
8017	+10 DIM	Dex	3	2	3	Int+4	14
8017	+10 DIM	Dex	3	2	3	Int+7	17
8017	+10 DIM	Dex	3	2	3	Int+14	24
8027	+3 DIM	Dex	3	2	3	precalving	-14
8027	+3 DIM	Dex	3	2	3	Intervention	3
8027	+3 DIM	Dex	3	2	3	Int+2	5
8027	+3 DIM	Dex	3	2	3	Int+4	7
8027	+3 DIM	Dex	3	2	3	Int+7	10
8027	+3 DIM	Dex	3	2	3	Int+14	17
8040	+3 DIM	Ins+Dex	3	2	3	precalving	-14
8040	+3 DIM	Ins+Dex	3	2	3	Intervention	3
8040	+3 DIM	Ins+Dex	3	2	3	Int+2	5
8040	+3 DIM	Ins+Dex	3	2	3	Int+4	7
8040	+3 DIM	Ins+Dex	3	2	3	Int+7	10
8040	+3 DIM	Ins+Dex	3	2	3	Int+14	17
8046	+3 DIM	Dex	3	2	3	precalving	-14
8046	+3 DIM	Dex	3	2	3	Intervention	3
8046	+3 DIM	Dex	3	2	3	Int+2	5
8046	+3 DIM	Dex	3	2	3	Int+4	7
8046	+3 DIM	Dex	3	2	3	Int+7	10
8046	+3 DIM	Dex	3	2	3	Int+14	17

Cow	time of intervention	Subgroup	Parity	Parity 2 g	parity 3 g	Time	DIM
8047	+10 DIM	Ins	3	2	3	precalving	-14
8047	+10 DIM	Ins	3	2	3	Intervention	10
8047	+10 DIM	Ins	3	2	3	Int+2	12
8047	+10 DIM	Ins	3	2	3	Int+4	14
8047	+10 DIM	Ins	3	2	3	Int+7	17
8047	+10 DIM	Ins	3	2	3	Int+14	24
8082	+10 DIM	Con	1	1	1	precalving	-14
8082	+10 DIM	Con	1	1	1	Intervention	10
8082	+10 DIM	Con	1	1	1	Int+2	12
8082	+10 DIM	Con	1	1	1	Int+4	14
8082	+10 DIM	Con	1	1	1	Int+7	17
8082	+10 DIM	Con	1	1	1	Int+14	24
8086	+10 DIM	Ins	3	2	3	precalving	-14
8086	+10 DIM	Ins	3	2	3	Intervention	10
8086	+10 DIM	Ins	3	2	3	Int+2	12
8086	+10 DIM	Ins	3	2	3	Int+4	14
8086	+10 DIM	Ins	3	2	3	Int+7	17
8086	+10 DIM	Ins	3	2	3	Int+14	24
8122	+10 DIM	Con	3	2	3	precalving	-14
8122	+10 DIM	Con	3	2	3	Intervention	10
8122	+10 DIM	Con	3	2	3	Int+2	12
8122	+10 DIM	Con	3	2	3	Int+4	14
8122	+10 DIM	Con	3	2	3	Int+7	17
8122	+10 DIM	Con	3	2	3	Int+14	24
8129	+10 DIM	Dex	3	2	3	precalving	-14
8129	+10 DIM	Dex	3	2	3	Intervention	10
8129	+10 DIM	Dex	3	2	3	Int+2	12
8129	+10 DIM	Dex	3	2	3	Int+4	14
8129	+10 DIM	Dex	3	2	3	Int+7	17
8129	+10 DIM	Dex	3	2	3	Int+14	24
8133	+10 DIM	Ins+Dex	3	2	3	precalving	-14
8133	+10 DIM	Ins+Dex	3	2	3	Intervention	10
8133	+10 DIM	Ins+Dex	3	2	3	Int+2	12
8133	+10 DIM	Ins+Dex	3	2	3	Int+4	14
8133	+10 DIM	Ins+Dex	3	2	3	Int+7	17
8133	+10 DIM	Ins+Dex	3	2	3	Int+14	24
8152	+10 DIM	Dex	3	2	3	precalving	-14
8152	+10 DIM	Dex	3	2	3	Intervention	10
8152	+10 DIM	Dex	3	2	3	Int+2	12
8152	+10 DIM	Dex	3	2	3	Int+4	14
8152	+10 DIM	Dex	3	2	3	Int+7	17
8152	+10 DIM	Dex	3	2	3	Int+14	24
8154	+10 DIM	Dex	3	2	3	precalving	-14
8154	+10 DIM	Dex	3	2	3	Intervention	10
8154	+10 DIM	Dex	3	2	3	Int+2	12
8154	+10 DIM	Dex	3	2	3	Int+4	14
8154	+10 DIM	Dex	3	2	3	Int+7	17
8154	+10 DIM	Dex	3	2	3	Int+14	24

Cow	time of intervention	Subgroup	Parity	Parity 2 g	parity 3 g	Time	DIM
8156	+3 DIM	Ins+Dex	3	2	3	precalving	-14
8156	+3 DIM	Ins+Dex	3	2	3	Intervention	3
8156	+3 DIM	Ins+Dex	3	2	3	Int+2	5
8156	+3 DIM	Ins+Dex	3	2	3	Int+4	7
8156	+3 DIM	Ins+Dex	3	2	3	Int+7	10
8156	+3 DIM	Ins+Dex	3	2	3	Int+14	17
8158	+10 DIM	Ins+Dex	3	2	3	precalving	-14
8158	+10 DIM	Ins+Dex	3	2	3	Intervention	10
8158	+10 DIM	Ins+Dex	3	2	3	Int+2	12
8158	+10 DIM	Ins+Dex	3	2	3	Int+4	14
8158	+10 DIM	Ins+Dex	3	2	3	Int+7	17
8158	+10 DIM	Ins+Dex	3	2	3	Int+14	24
8160	+3 DIM	Dex	3	2	3	precalving	-14
8160	+3 DIM	Dex	3	2	3	Intervention	3
8160	+3 DIM	Dex	3	2	3	Int+2	5
8160	+3 DIM	Dex	3	2	3	Int+4	7
8160	+3 DIM	Dex	3	2	3	Int+7	10
8160	+3 DIM	Dex	3	2	3	Int+14	17
8177	+3 DIM	Ins+Dex	3	2	3	precalving	-14
8177	+3 DIM	Ins+Dex	3	2	3	Intervention	3
8177	+3 DIM	Ins+Dex	3	2	3	Int+2	5
8177	+3 DIM	Ins+Dex	3	2	3	Int+4	7
8177	+3 DIM	Ins+Dex	3	2	3	Int+7	10
8177	+3 DIM	Ins+Dex	3	2	3	Int+14	17
8181	+3 DIM	Ins	3	2	3	precalving	-14
8181	+3 DIM	Ins	3	2	3	Intervention	3
8181	+3 DIM	Ins	3	2	3	Int+2	5
8181	+3 DIM	Ins	3	2	3	Int+4	7
8181	+3 DIM	Ins	3	2	3	Int+7	10
8181	+3 DIM	Ins	3	2	3	Int+14	17
8186	+3 DIM	Con	3	2	3	precalving	-14
8186	+3 DIM	Con	3	2	3	Intervention	3
8186	+3 DIM	Con	3	2	3	Int+2	5
8186	+3 DIM	Con	3	2	3	Int+4	7
8186	+3 DIM	Con	3	2	3	Int+7	10
8186	+3 DIM	Con	3	2	3	Int+14	17
8221	+3 DIM	Con	3	2	3	precalving	-14
8221	+3 DIM	Con	3	2	3	Intervention	3
8221	+3 DIM	Con	3	2	3	Int+2	5
8221	+3 DIM	Con	3	2	3	Int+4	7
8221	+3 DIM	Con	3	2	3	Int+7	10
8221	+3 DIM	Con	3	2	3	Int+14	17
8230	+3 DIM	Ins	3	2	3	precalving	-14
8230	+3 DIM	Ins	3	2	3	Intervention	3
8230	+3 DIM	Ins	3	2	3	Int+2	5
8230	+3 DIM	Ins	3	2	3	Int+4	7
8230	+3 DIM	Ins	3	2	3	Int+7	10
8230	+3 DIM	Ins	3	2	3	Int+14	17

Cow	time of intervention	Subgroup	Parity	Parity 2 g	parity 3 g	Time	DIM
8244	+3 DIM	Ins	3	2	3	precalving	-14
8244	+3 DIM	Ins	3	2	3	Intervention	3
8244	+3 DIM	Ins	3	2	3	Int+2	5
8244	+3 DIM	Ins	3	2	3	Int+4	7
8244	+3 DIM	Ins	3	2	3	Int+7	10
8244	+3 DIM	Ins	3	2	3	Int+14	17
8252	+3 DIM	Dex	3	2	3	precalving	-14
8252	+3 DIM	Dex	3	2	3	Intervention	3
8252	+3 DIM	Dex	3	2	3	Int+2	5
8252	+3 DIM	Dex	3	2	3	Int+4	7
8252	+3 DIM	Dex	3	2	3	Int+7	10
8252	+3 DIM	Dex	3	2	3	Int+14	17
8268	+3 DIM	Dex	3	2	3	precalving	-14
8268	+3 DIM	Dex	3	2	3	Intervention	3
8268	+3 DIM	Dex	3	2	3	Int+2	5
8268	+3 DIM	Dex	3	2	3	Int+4	7
8268	+3 DIM	Dex	3	2	3	Int+7	10
8268	+3 DIM	Dex	3	2	3	Int+14	17
8313	+3 DIM	Ins	3	2	3	precalving	-14
8313	+3 DIM	Ins	3	2	3	Intervention	3
8313	+3 DIM	Ins	3	2	3	Int+2	5
8313	+3 DIM	Ins	3	2	3	Int+4	7
8313	+3 DIM	Ins	3	2	3	Int+7	10
8313	+3 DIM	Ins	3	2	3	Int+14	17
8339	+10 DIM	Dex	2	2	2	precalving	-14
8339	+10 DIM	Dex	2	2	2	Intervention	10
8339	+10 DIM	Dex	2	2	2	Int+2	12
8339	+10 DIM	Dex	2	2	2	Int+4	14
8339	+10 DIM	Dex	2	2	2	Int+7	17
8339	+10 DIM	Dex	2	2	2	Int+14	24
8372	+3 DIM	Ins+Dex	3	2	3	precalving	-14
8372	+3 DIM	Ins+Dex	3	2	3	Intervention	3
8372	+3 DIM	Ins+Dex	3	2	3	Int+2	5
8372	+3 DIM	Ins+Dex	3	2	3	Int+4	7
8372	+3 DIM	Ins+Dex	3	2	3	Int+7	10
8372	+3 DIM	Ins+Dex	3	2	3	Int+14	17
8375	+10 DIM	Con	3	2	3	precalving	-14
8375	+10 DIM	Con	3	2	3	Intervention	10
8375	+10 DIM	Con	3	2	3	Int+2	12
8375	+10 DIM	Con	3	2	3	Int+4	14
8375	+10 DIM	Con	3	2	3	Int+7	17
8375	+10 DIM	Con	3	2	3	Int+14	24
8376	+10 DIM	Con	3	2	3	precalving	-14
8376	+10 DIM	Con	3	2	3	Intervention	10
8376	+10 DIM	Con	3	2	3	Int+2	12
8376	+10 DIM	Con	3	2	3	Int+4	14
8376	+10 DIM	Con	3	2	3	Int+7	17
8376	+10 DIM	Con	3	2	3	Int+14	24

Cow	time of intervention	Subgroup	Parity	Parity 2 g	parity 3 g	Time	DIM
8389	+3 DIM	Dex	3	2	3	precalving	-14
8389	+3 DIM	Dex	3	2	3	Intervention	3
8389	+3 DIM	Dex	3	2	3	Int+2	5
8389	+3 DIM	Dex	3	2	3	Int+4	7
8389	+3 DIM	Dex	3	2	3	Int+7	10
8389	+3 DIM	Dex	3	2	3	Int+14	17
8393	+10 DIM	Dex	3	2	3	precalving	-14
8393	+10 DIM	Dex	3	2	3	Intervention	10
8393	+10 DIM	Dex	3	2	3	Int+2	12
8393	+10 DIM	Dex	3	2	3	Int+4	14
8393	+10 DIM	Dex	3	2	3	Int+7	17
8393	+10 DIM	Dex	3	2	3	Int+14	24
8398	+10 DIM	Ins	2	2	2	precalving	-14
8398	+10 DIM	Ins	2	2	2	Intervention	10
8398	+10 DIM	Ins	2	2	2	Int+2	12
8398	+10 DIM	Ins	2	2	2	Int+4	14
8398	+10 DIM	Ins	2	2	2	Int+7	17
8398	+10 DIM	Ins	2	2	2	Int+14	24
8401	+3 DIM	Ins+Dex	3	2	3	precalving	-14
8401	+3 DIM	Ins+Dex	3	2	3	Intervention	3
8401	+3 DIM	Ins+Dex	3	2	3	Int+2	5
8401	+3 DIM	Ins+Dex	3	2	3	Int+4	7
8401	+3 DIM	Ins+Dex	3	2	3	Int+7	10
8401	+3 DIM	Ins+Dex	3	2	3	Int+14	17
8402	+3 DIM	Ins+Dex	3	2	3	precalving	-14
8402	+3 DIM	Ins+Dex	3	2	3	Intervention	3
8402	+3 DIM	Ins+Dex	3	2	3	Int+2	5
8402	+3 DIM	Ins+Dex	3	2	3	Int+4	7
8402	+3 DIM	Ins+Dex	3	2	3	Int+7	10
8402	+3 DIM	Ins+Dex	3	2	3	Int+14	17
8414	+10 DIM	Ins+Dex	3	2	3	precalving	-14
8414	+10 DIM	Ins+Dex	3	2	3	Intervention	10
8414	+10 DIM	Ins+Dex	3	2	3	Int+2	12
8414	+10 DIM	Ins+Dex	3	2	3	Int+4	14
8414	+10 DIM	Ins+Dex	3	2	3	Int+7	17
8414	+10 DIM	Ins+Dex	3	2	3	Int+14	24
8469	+10 DIM	Ins+Dex	2	2	2	precalving	-14
8469	+10 DIM	Ins+Dex	2	2	2	Intervention	10
8469	+10 DIM	Ins+Dex	2	2	2	Int+2	12
8469	+10 DIM	Ins+Dex	2	2	2	Int+4	14
8469	+10 DIM	Ins+Dex	2	2	2	Int+7	17
8469	+10 DIM	Ins+Dex	2	2	2	Int+14	24
8510	+3 DIM	Ins	2	2	2	precalving	-14
8510	+3 DIM	Ins	2	2	2	Intervention	3
8510	+3 DIM	Ins	2	2	2	Int+2	5
8510	+3 DIM	Ins	2	2	2	Int+4	7
8510	+3 DIM	Ins	2	2	2	Int+7	10
8510	+3 DIM	Ins	2	2	2	Int+14	17

Cow	time of intervention	Subgroup	Parity	Parity 2 g	parity 3 g	Time	DIM
8522	+3 DIM	Con	2	2	2	precalving	-14
8522	+3 DIM	Con	2	2	2	Intervention	3
8522	+3 DIM	Con	2	2	2	Int+2	5
8522	+3 DIM	Con	2	2	2	Int+4	7
8522	+3 DIM	Con	2	2	2	Int+7	10
8522	+3 DIM	Con	2	2	2	Int+14	17
8599	+10 DIM	Dex	2	2	2	precalving	-14
8599	+10 DIM	Dex	2	2	2	Intervention	10
8599	+10 DIM	Dex	2	2	2	Int+2	12
8599	+10 DIM	Dex	2	2	2	Int+4	14
8599	+10 DIM	Dex	2	2	2	Int+7	17
8599	+10 DIM	Dex	2	2	2	Int+14	24
8684	+3 DIM	Ins	2	2	2	precalving	-14
8684	+3 DIM	Ins	2	2	2	Intervention	3
8684	+3 DIM	Ins	2	2	2	Int+2	5
8684	+3 DIM	Ins	2	2	2	Int+4	7
8684	+3 DIM	Ins	2	2	2	Int+7	10
8684	+3 DIM	Ins	2	2	2	Int+14	17
8704	+3 DIM	Ins	2	2	2	precalving	-14
8704	+3 DIM	Ins	2	2	2	Intervention	3
8704	+3 DIM	Ins	2	2	2	Int+2	5
8704	+3 DIM	Ins	2	2	2	Int+4	7
8704	+3 DIM	Ins	2	2	2	Int+7	10
8704	+3 DIM	Ins	2	2	2	Int+14	17
8706	+3 DIM	Ins	2	2	2	precalving	-14
8706	+3 DIM	Ins	2	2	2	Intervention	3
8706	+3 DIM	Ins	2	2	2	Int+2	5
8706	+3 DIM	Ins	2	2	2	Int+4	7
8706	+3 DIM	Ins	2	2	2	Int+7	10
8706	+3 DIM	Ins	2	2	2	Int+14	17
9004	+10 DIM	Con	2	2	2	precalving	-14
9004	+10 DIM	Con	2	2	2	Intervention	10
9004	+10 DIM	Con	2	2	2	Int+2	12
9004	+10 DIM	Con	2	2	2	Int+4	14
9004	+10 DIM	Con	2	2	2	Int+7	17
9004	+10 DIM	Con	2	2	2	Int+14	24
9027	+3 DIM	Dex	2	2	2	precalving	-14
9027	+3 DIM	Dex	2	2	2	Intervention	3
9027	+3 DIM	Dex	2	2	2	Int+2	5
9027	+3 DIM	Dex	2	2	2	Int+4	7
9027	+3 DIM	Dex	2	2	2	Int+7	10
9027	+3 DIM	Dex	2	2	2	Int+14	17
9028	+10 DIM	Ins	2	2	2	precalving	-14
9028	+10 DIM	Ins	2	2	2	Intervention	10
9028	+10 DIM	Ins	2	2	2	Int+2	12
9028	+10 DIM	Ins	2	2	2	Int+4	14
9028	+10 DIM	Ins	2	2	2	Int+7	17
9028	+10 DIM	Ins	2	2	2	Int+14	24

Cow	time of intervention	Subgroup	Parity	Parity 2 g	parity 3 g	Time	DIM
9030	+3 DIM	Dex	2	2	2	precalving	-14
9030	+3 DIM	Dex	2	2	2	Intervention	3
9030	+3 DIM	Dex	2	2	2	Int+2	5
9030	+3 DIM	Dex	2	2	2	Int+4	7
9030	+3 DIM	Dex	2	2	2	Int+7	10
9030	+3 DIM	Dex	2	2	2	Int+14	17
9031	+10 DIM	Ins	2	2	2	precalving	-14
9031	+10 DIM	Ins	2	2	2	Intervention	10
9031	+10 DIM	Ins	2	2	2	Int+2	12
9031	+10 DIM	Ins	2	2	2	Int+4	14
9031	+10 DIM	Ins	2	2	2	Int+7	17
9031	+10 DIM	Ins	2	2	2	Int+14	24
9060	+3 DIM	Dex	2	2	2	precalving	-14
9060	+3 DIM	Dex	2	2	2	Intervention	3
9060	+3 DIM	Dex	2	2	2	Int+2	5
9060	+3 DIM	Dex	2	2	2	Int+4	7
9060	+3 DIM	Dex	2	2	2	Int+7	10
9060	+3 DIM	Dex	2	2	2	Int+14	17
9066	+3 DIM	Ins+Dex	2	2	2	precalving	-14
9066	+3 DIM	Ins+Dex	2	2	2	Intervention	3
9066	+3 DIM	Ins+Dex	2	2	2	Int+2	5
9066	+3 DIM	Ins+Dex	2	2	2	Int+4	7
9066	+3 DIM	Ins+Dex	2	2	2	Int+7	10
9066	+3 DIM	Ins+Dex	2	2	2	Int+14	17
9074	+10 DIM	Ins+Dex	2	2	2	precalving	-14
9074	+10 DIM	Ins+Dex	2	2	2	Intervention	10
9074	+10 DIM	Ins+Dex	2	2	2	Int+2	12
9074	+10 DIM	Ins+Dex	2	2	2	Int+4	14
9074	+10 DIM	Ins+Dex	2	2	2	Int+7	17
9074	+10 DIM	Ins+Dex	2	2	2	Int+14	24
9087	+10 DIM	Con	2	2	2	precalving	-14
9087	+10 DIM	Con	2	2	2	Intervention	10
9087	+10 DIM	Con	2	2	2	Int+2	12
9087	+10 DIM	Con	2	2	2	Int+4	14
9087	+10 DIM	Con	2	2	2	Int+7	17
9087	+10 DIM	Con	2	2	2	Int+14	24
9092	+3 DIM	Dex	2	2	2	precalving	-14
9092	+3 DIM	Dex	2	2	2	Intervention	3
9092	+3 DIM	Dex	2	2	2	Int+2	5
9092	+3 DIM	Dex	2	2	2	Int+4	7
9092	+3 DIM	Dex	2	2	2	Int+7	10
9092	+3 DIM	Dex	2	2	2	Int+14	17
9101	+10 DIM	Con	2	2	2	precalving	-14
9101	+10 DIM	Con	2	2	2	Intervention	10
9101	+10 DIM	Con	2	2	2	Int+2	12
9101	+10 DIM	Con	2	2	2	Int+4	14
9101	+10 DIM	Con	2	2	2	Int+7	17
9101	+10 DIM	Con	2	2	2	Int+14	24

Cow	time of intervention	Subgroup	Parity	Parity 2 g	parity 3 g	Time	DIM
9112	+10 DIM	Con	2	2	2	precalving	-14
9112	+10 DIM	Con	2	2	2	Intervention	10
9112	+10 DIM	Con	2	2	2	Int+2	12
9112	+10 DIM	Con	2	2	2	Int+4	14
9112	+10 DIM	Con	2	2	2	Int+7	17
9112	+10 DIM	Con	2	2	2	Int+14	24
9123	+10 DIM	Ins+Dex	2	2	2	precalving	-14
9123	+10 DIM	Ins+Dex	2	2	2	Intervention	10
9123	+10 DIM	Ins+Dex	2	2	2	Int+2	12
9123	+10 DIM	Ins+Dex	2	2	2	Int+4	14
9123	+10 DIM	Ins+Dex	2	2	2	Int+7	17
9123	+10 DIM	Ins+Dex	2	2	2	Int+14	24
9128	+10 DIM	Con	2	2	2	precalving	-14
9128	+10 DIM	Con	2	2	2	Intervention	10
9128	+10 DIM	Con	2	2	2	Int+2	12
9128	+10 DIM	Con	2	2	2	Int+4	14
9128	+10 DIM	Con	2	2	2	Int+7	17
9128	+10 DIM	Con	2	2	2	Int+14	24
9132	+10 DIM	Dex	2	2	2	precalving	-14
9132	+10 DIM	Dex	2	2	2	Intervention	10
9132	+10 DIM	Dex	2	2	2	Int+2	12
9132	+10 DIM	Dex	2	2	2	Int+4	14
9132	+10 DIM	Dex	2	2	2	Int+7	17
9132	+10 DIM	Dex	2	2	2	Int+14	24
9141	+3 DIM	Con	2	2	2	precalving	-14
9141	+3 DIM	Con	2	2	2	Intervention	3
9141	+3 DIM	Con	2	2	2	Int+2	5
9141	+3 DIM	Con	2	2	2	Int+4	7
9141	+3 DIM	Con	2	2	2	Int+7	10
9141	+3 DIM	Con	2	2	2	Int+14	17
9147	+3 DIM	Ins+Dex	2	2	2	precalving	-14
9147	+3 DIM	Ins+Dex	2	2	2	Intervention	3
9147	+3 DIM	Ins+Dex	2	2	2	Int+2	5
9147	+3 DIM	Ins+Dex	2	2	2	Int+4	7
9147	+3 DIM	Ins+Dex	2	2	2	Int+7	10
9147	+3 DIM	Ins+Dex	2	2	2	Int+14	17
9160	+3 DIM	Ins+Dex	2	2	2	precalving	-14
9160	+3 DIM	Ins+Dex	2	2	2	Intervention	3
9160	+3 DIM	Ins+Dex	2	2	2	Int+2	5
9160	+3 DIM	Ins+Dex	2	2	2	Int+4	7
9160	+3 DIM	Ins+Dex	2	2	2	Int+7	10
9160	+3 DIM	Ins+Dex	2	2	2	Int+14	17
9166	+3 DIM	Con	2	2	2	precalving	-14
9166	+3 DIM	Con	2	2	2	Intervention	3
9166	+3 DIM	Con	2	2	2	Int+2	5
9166	+3 DIM	Con	2	2	2	Int+4	7
9166	+3 DIM	Con	2	2	2	Int+7	10
9166	+3 DIM	Con	2	2	2	Int+14	17

Cow	time of intervention	Subgroup	Parity	Parity 2 g	parity 3 g	Time	DIM
9167	+10 DIM	Ins	2	2	2	precalving	-14
9167	+10 DIM	Ins	2	2	2	Intervention	10
9167	+10 DIM	Ins	2	2	2	Int+2	12
9167	+10 DIM	Ins	2	2	2	Int+4	14
9167	+10 DIM	Ins	2	2	2	Int+7	17
9167	+10 DIM	Ins	2	2	2	Int+14	24
9168	+3 DIM	Con	2	2	2	precalving	-14
9168	+3 DIM	Con	2	2	2	Intervention	3
9168	+3 DIM	Con	2	2	2	Int+2	5
9168	+3 DIM	Con	2	2	2	Int+4	7
9168	+3 DIM	Con	2	2	2	Int+7	10
9168	+3 DIM	Con	2	2	2	Int+14	17
9178	+3 DIM	Ins	2	2	2	precalving	-14
9178	+3 DIM	Ins	2	2	2	Intervention	3
9178	+3 DIM	Ins	2	2	2	Int+2	5
9178	+3 DIM	Ins	2	2	2	Int+4	7
9178	+3 DIM	Ins	2	2	2	Int+7	10
9178	+3 DIM	Ins	2	2	2	Int+14	17
9181	+10 DIM	Ins+Dex	2	2	2	precalving	-14
9181	+10 DIM	Ins+Dex	2	2	2	Intervention	10
9181	+10 DIM	Ins+Dex	2	2	2	Int+2	12
9181	+10 DIM	Ins+Dex	2	2	2	Int+4	14
9181	+10 DIM	Ins+Dex	2	2	2	Int+7	17
9181	+10 DIM	Ins+Dex	2	2	2	Int+14	24
9194	+3 DIM	Dex	2	2	2	precalving	-14
9194	+3 DIM	Dex	2	2	2	Intervention	3
9194	+3 DIM	Dex	2	2	2	Int+2	5
9194	+3 DIM	Dex	2	2	2	Int+4	7
9194	+3 DIM	Dex	2	2	2	Int+7	10
9194	+3 DIM	Dex	2	2	2	Int+14	17
9204	+3 DIM	Ins+Dex	2	2	2	precalving	-14
9204	+3 DIM	Ins+Dex	2	2	2	Intervention	3
9204	+3 DIM	Ins+Dex	2	2	2	Int+2	5
9204	+3 DIM	Ins+Dex	2	2	2	Int+4	7
9204	+3 DIM	Ins+Dex	2	2	2	Int+7	10
9204	+3 DIM	Ins+Dex	2	2	2	Int+14	17
9210	+10 DIM	Dex	2	2	2	precalving	-14
9210	+10 DIM	Dex	2	2	2	Intervention	10
9210	+10 DIM	Dex	2	2	2	Int+2	12
9210	+10 DIM	Dex	2	2	2	Int+4	14
9210	+10 DIM	Dex	2	2	2	Int+7	17
9210	+10 DIM	Dex	2	2	2	Int+14	24
9211	+3 DIM	Con	2	2	2	precalving	-14
9211	+3 DIM	Con	2	2	2	Intervention	3
9211	+3 DIM	Con	2	2	2	Int+2	5
9211	+3 DIM	Con	2	2	2	Int+4	7
9211	+3 DIM	Con	2	2	2	Int+7	10
9211	+3 DIM	Con	2	2	2	Int+14	17

Cow	time of intervention	Subgroup	Parity	Parity 2 g	parity 3 g	Time	DIM
9218	+3 DIM	Dex	2	2	2	precalving	-14
9218	+3 DIM	Dex	2	2	2	Intervention	3
9218	+3 DIM	Dex	2	2	2	Int+2	5
9218	+3 DIM	Dex	2	2	2	Int+4	7
9218	+3 DIM	Dex	2	2	2	Int+7	10
9218	+3 DIM	Dex	2	2	2	Int+14	17
9226	+3 DIM	Con	1	1	1	precalving	-14
9226	+3 DIM	Con	1	1	1	Intervention	3
9226	+3 DIM	Con	1	1	1	Int+2	5
9226	+3 DIM	Con	1	1	1	Int+4	7
9226	+3 DIM	Con	1	1	1	Int+7	10
9226	+3 DIM	Con	1	1	1	Int+14	17
9228	+10 DIM	Ins+Dex	2	2	2	precalving	-14
9228	+10 DIM	Ins+Dex	2	2	2	Intervention	10
9228	+10 DIM	Ins+Dex	2	2	2	Int+2	12
9228	+10 DIM	Ins+Dex	2	2	2	Int+4	14
9228	+10 DIM	Ins+Dex	2	2	2	Int+7	17
9228	+10 DIM	Ins+Dex	2	2	2	Int+14	24
9229	+3 DIM	Dex	2	2	2	precalving	-14
9229	+3 DIM	Dex	2	2	2	Intervention	3
9229	+3 DIM	Dex	2	2	2	Int+2	5
9229	+3 DIM	Dex	2	2	2	Int+4	7
9229	+3 DIM	Dex	2	2	2	Int+7	10
9229	+3 DIM	Dex	2	2	2	Int+14	17
9290	+3 DIM	Ins+Dex	2	2	2	precalving	-14
9290	+3 DIM	Ins+Dex	2	2	2	Intervention	3
9290	+3 DIM	Ins+Dex	2	2	2	Int+2	5
9290	+3 DIM	Ins+Dex	2	2	2	Int+4	7
9290	+3 DIM	Ins+Dex	2	2	2	Int+7	10
9290	+3 DIM	Ins+Dex	2	2	2	Int+14	17
9313	+10 DIM	Con	2	2	2	precalving	-14
9313	+10 DIM	Con	2	2	2	Intervention	10
9313	+10 DIM	Con	2	2	2	Int+2	12
9313	+10 DIM	Con	2	2	2	Int+4	14
9313	+10 DIM	Con	2	2	2	Int+7	17
9313	+10 DIM	Con	2	2	2	Int+14	24
9341	+10 DIM	Ins	2	2	2	precalving	-14
9341	+10 DIM	Ins	2	2	2	Intervention	10
9341	+10 DIM	Ins	2	2	2	Int+2	12
9341	+10 DIM	Ins	2	2	2	Int+4	14
9341	+10 DIM	Ins	2	2	2	Int+7	17
9341	+10 DIM	Ins	2	2	2	Int+14	24
9352	+10 DIM	Ins	2	2	2	precalving	-14
9352	+10 DIM	Ins	2	2	2	Intervention	10
9352	+10 DIM	Ins	2	2	2	Int+2	12
9352	+10 DIM	Ins	2	2	2	Int+4	14
9352	+10 DIM	Ins	2	2	2	Int+7	17
9352	+10 DIM	Ins	2	2	2	Int+14	24

Cow	time of intervention	Subgroup	Parity	Parity 2 g	parity 3 g	Time	DIM
9358	+10 DIM	Con	2	2	2	precalving	-14
9358	+10 DIM	Con	2	2	2	Intervention	10
9358	+10 DIM	Con	2	2	2	Int+2	12
9358	+10 DIM	Con	2	2	2	Int+4	14
9358	+10 DIM	Con	2	2	2	Int+7	17
9358	+10 DIM	Con	2	2	2	Int+14	24
9359	+10 DIM	Ins+Dex	2	2	2	precalving	-14
9359	+10 DIM	Ins+Dex	2	2	2	Intervention	10
9359	+10 DIM	Ins+Dex	2	2	2	Int+2	12
9359	+10 DIM	Ins+Dex	2	2	2	Int+4	14
9359	+10 DIM	Ins+Dex	2	2	2	Int+7	17
9359	+10 DIM	Ins+Dex	2	2	2	Int+14	24
9365	+3 DIM	Ins+Dex	2	2	2	precalving	-14
9365	+3 DIM	Ins+Dex	2	2	2	Intervention	3
9365	+3 DIM	Ins+Dex	2	2	2	Int+2	5
9365	+3 DIM	Ins+Dex	2	2	2	Int+4	7
9365	+3 DIM	Ins+Dex	2	2	2	Int+7	10
9365	+3 DIM	Ins+Dex	2	2	2	Int+14	17
9375	+10 DIM	Ins+Dex	1	1	1	precalving	-14
9375	+10 DIM	Ins+Dex	1	1	1	Intervention	10
9375	+10 DIM	Ins+Dex	1	1	1	Int+2	12
9375	+10 DIM	Ins+Dex	1	1	1	Int+4	14
9375	+10 DIM	Ins+Dex	1	1	1	Int+7	17
9375	+10 DIM	Ins+Dex	1	1	1	Int+14	24
9377	+3 DIM	Con	2	2	2	precalving	-14
9377	+3 DIM	Con	2	2	2	Intervention	3
9377	+3 DIM	Con	2	2	2	Int+2	5
9377	+3 DIM	Con	2	2	2	Int+4	7
9377	+3 DIM	Con	2	2	2	Int+7	10
9377	+3 DIM	Con	2	2	2	Int+14	17
9435	+3 DIM	Con	1	1	1	precalving	-14
9435	+3 DIM	Con	1	1	1	Intervention	3
9435	+3 DIM	Con	1	1	1	Int+2	5
9435	+3 DIM	Con	1	1	1	Int+4	7
9435	+3 DIM	Con	1	1	1	Int+7	10
9435	+3 DIM	Con	1	1	1	Int+14	17
9587	+10 DIM	Dex	1	1	1	precalving	-14
9587	+10 DIM	Dex	1	1	1	Intervention	10
9587	+10 DIM	Dex	1	1	1	Int+2	12
9587	+10 DIM	Dex	1	1	1	Int+4	14
9587	+10 DIM	Dex	1	1	1	Int+7	17
9587	+10 DIM	Dex	1	1	1	Int+14	24
9594	+10 DIM	Ins+Dex	1	1	1	precalving	-14
9594	+10 DIM	Ins+Dex	1	1	1	Intervention	10
9594	+10 DIM	Ins+Dex	1	1	1	Int+2	12
9594	+10 DIM	Ins+Dex	1	1	1	Int+4	14
9594	+10 DIM	Ins+Dex	1	1	1	Int+7	17
9594	+10 DIM	Ins+Dex	1	1	1	Int+14	24

Cow	time of intervention	Subgroup	Parity	Parity 2 g	parity 3 g	Time	DIM
9929	+3 DIM	Ins	2	2	2	precalving	-14
9929	+3 DIM	Ins	2	2	2	Intervention	3
9929	+3 DIM	Ins	2	2	2	Int+2	5
9929	+3 DIM	Ins	2	2	2	Int+4	7
9929	+3 DIM	Ins	2	2	2	Int+7	10
9929	+3 DIM	Ins	2	2	2	Int+14	17
9948	+10 DIM	Dex	2	2	2	precalving	-14
9948	+10 DIM	Dex	2	2	2	Intervention	10
9948	+10 DIM	Dex	2	2	2	Int+2	12
9948	+10 DIM	Dex	2	2	2	Int+4	14
9948	+10 DIM	Dex	2	2	2	Int+7	17
9948	+10 DIM	Dex	2	2	2	Int+14	24
51008	+10 DIM	Ins+Dex	6	2	3	precalving	-14
51008	+10 DIM	Ins+Dex	6	2	3	Intervention	10
51008	+10 DIM	Ins+Dex	6	2	3	Int+2	12
51008	+10 DIM	Ins+Dex	6	2	3	Int+4	14
51008	+10 DIM	Ins+Dex	6	2	3	Int+7	17
51008	+10 DIM	Ins+Dex	6	2	3	Int+14	24
51108	+10 DIM	Con	5	2	3	precalving	-14
51108	+10 DIM	Con	5	2	3	Intervention	10
51108	+10 DIM	Con	5	2	3	Int+2	12
51108	+10 DIM	Con	5	2	3	Int+4	14
51108	+10 DIM	Con	5	2	3	Int+7	17
51108	+10 DIM	Con	5	2	3	Int+14	24
55112	+3 DIM	Dex	4	2	3	precalving	-14
55112	+3 DIM	Dex	4	2	3	Intervention	3
55112	+3 DIM	Dex	4	2	3	Int+2	5
55112	+3 DIM	Dex	4	2	3	Int+4	7
55112	+3 DIM	Dex	4	2	3	Int+7	10
55112	+3 DIM	Dex	4	2	3	Int+14	17
81580	+3 DIM	Ins	3	2	3	precalving	-14
81580	+3 DIM	Ins	3	2	3	Intervention	3
81580	+3 DIM	Ins	3	2	3	Int+2	5
81580	+3 DIM	Ins	3	2	3	Int+4	7
81580	+3 DIM	Ins	3	2	3	Int+7	10
81580	+3 DIM	Ins	3	2	3	Int+14	17
82520	+3 DIM	Ins	2	2	2	precalving	-14
82520	+3 DIM	Ins	2	2	2	Intervention	3
82520	+3 DIM	Ins	2	2	2	Int+2	5
82520	+3 DIM	Ins	2	2	2	Int+4	7
82520	+3 DIM	Ins	2	2	2	Int+7	10
82520	+3 DIM	Ins	2	2	2	Int+14	17
91410	+10 DIM	Dex	2	2	2	precalving	-14
91410	+10 DIM	Dex	2	2	2	Intervention	10
91410	+10 DIM	Dex	2	2	2	Int+2	12
91410	+10 DIM	Dex	2	2	2	Int+4	14
91410	+10 DIM	Dex	2	2	2	Int+7	17
91410	+10 DIM	Dex	2	2	2	Int+14	24

Cow	time of intervention	Subgroup	Parity	Parity 2 g	parity 3 g	Time	DIM
91780	+3 DIM	Con	2	2	2	precalving	-14
91780	+3 DIM	Con	2	2	2	Intervention	3
91780	+3 DIM	Con	2	2	2	Int+2	5
91780	+3 DIM	Con	2	2	2	Int+4	7
91780	+3 DIM	Con	2	2	2	Int+7	10
91780	+3 DIM	Con	2	2	2	Int+14	17

Cow	BCS	BCSg	NEFA	BHBA	GLU	Alb	Urea	AST
28	3.75	fat	0.14	0.309	73	3.6	18	42
28	3.5	fair	0.86	0.773	74	4.2	19	83
28	3.5	fair	0.967	0.656	54	3.7	16	65
28	3.5	fair	1.56	1.32	39	4	18	73
28	3.25	fair	0.813	0.879	72	4	15	84
28	3.25	fair	0.496	0.543	76	4	16	65
47	3.25	fair	0.247	0.532	34	4	16	61
47	3	fair	0.711	0.591	66	3.3	20	86
47	3	fair	0.918	1.26	66	3.6	15	96
47	3	fair	1.11	1.43	65	3.5	19	101
47	2.25	thin	0.533	1.09	57	3.2	15	118
47	2.25	thin	0.186	0.484	56	2.9	13	96
56	4	fat	0.086	0.42	88	4.1	21	62
56	4	fat	0.429	0.388	60	3.9	19	88
56	4	fat	0.538	0.895	60	3.8	19	67
56	4	fat	0.786	0.68	64	4.2	24	74
56	3.25	fair	0.912	1.01	63	4.3	21	58
56	3.25	fair	0.776	0.949	79	3.9	19	69
67	4	fat	0.144	0.504	70	4.1	21	74
67	4	fat	0.704	0.999	65	3.8	17	83
67	4	fat	0.824	1.18	74	4.2	18	112
67	4	fat	0.433	0.679	57	3.9	20	97
67	3.5	fair	0.912	0.691	76	4.2	21	101
67	3.5	fair	0.422	0.451	74	4.2	20	88
71	4.25	fat	0.108	0.434	56	4.3	23	65
71	4.5	fat	0.469	0.583	69	3.7	17	117
71	4.5	fat	0.613	0.42	52	4	21	81
71	4.5	fat	0.385	0.485	53	3.7	17	58
71	3.25	fair	0.605	0.512	65	3.9	19	64
71	3.25	fair	0.355	0.461	62	4	22	70
78	3.5	fair	0.174	0.606	81	3.9	23	75
78	3.5	fair	0.569	0.682	73	3.9	21	105
78	3.5	fair	0.339	0.809	77	3.5	17	90
78	3.5	fair	0.529	0.779	86	3.9	18	93
78	4.25	fat	0.395	0.63	86	4	26	83
78	4.25	fat	0.403	0.645	74	4	26	75
94	3.75	fat	0.053	0.342	71	3.3	16	57
94	3.5	fair	0.625	0.621	92	3.5	17	116
94	3.5	fair	0.184	0.323	66	3.6	17	129
94	3.5	fair	0.27	0.418	57	4	13	91
94	2.25	thin	0.707	0.757	72	3.8	12	84
94	2.25	thin	0.268	.	65	3.8	20	71
95	4	fat	0.181	0.448	62	4	25	72
95	3.75	fat	0.643	0.422	37	3.4	16	95
95	3.75	fat	0.834	0.474	50	3.6	18	98
95	3.75	fat	0.534	0.417	59	3.8	21	88
95	3.5	fair	0.281	0.311	44	3.2	19	77
95	3.5	fair	0.235	0.493	53	3.8	22	82

Cow	BCS	BCSg	NEFA	BHBA	GLU	Alb	Urea	AST
99	3.75	fat	0.13	0.446	51	3.9	29	35
99	4.25	fat	0.522	0.691	39	4	26	80
99	4.25	fat	0.374	0.404	38	3.8	28	69
99	4.25	fat	0.677	0.355	34	4	24	69
99	3.5	fair	0.369	0.541	37	3.7	20	53
99	3.5	fair	0.258	0.217	62	4.2	23	62
102	3.75	fat	0.137	0.417	53	3.8	19	76
102	2.5	thin	0.746	0.491	47	3.7	17	94
102	2.5	thin	0.288	0.385	53	3.4	12	99
102	2.5	thin	0.538	0.47	54	3.7	13	115
102	2.5	thin	0.223	0.419	45	3.6	19	84
102	2.5	thin	0.227	0.443	54	3.7	18	70
106	3.5	fair	0.082	0.489	50	4	24	73
106	2.5	thin	0.615	0.818	33	3.5	23	71
106	2.5	thin	0.625	0.689	52	3.7	23	76
106	2.5	thin	0.322	0.655	42	4	24	72
106	3.25	fair	0.268	0.419	35	3.9	21	65
106	3.25	fair	0.279	.	53	3.8	26	69
107	3.75	fat	0.098	0.396	49	3.8	25	77
107	4.25	fat	0.569	0.61	53	3.7	21	119
107	4.25	fat	0.503	0.639	49	3.5	25	122
107	4.25	fat	0.865	0.967	43	3.9	22	129
107	3.5	fair	0.355	0.52	36	3.9	22	115
107	3.5	fair	0.627	.	55	4.4	26	131
110	4.25	fat	0.18	0.485	78	4.3	24	60
110	4.25	fat	0.447	0.605	75	3.9	24	65
110	4.25	fat	0.434	0.583	75	4.3	26	53
110	4.25	fat	0.33	0.955	65	3.8	23	42
110	4	fat	0.606	0.702	84	4	16	43
110	4	fat	0.33	0.515	71	3.8	24	44
112	3.5	fair	0.472	0.402	83	3.9	21	53
112	2.25	thin	0.519	0.465	66	3.1	14	83
112	2.25	thin	0.365	0.428	60	3.2	14	80
112	2.25	thin	0.261	0.585	63	3.2	14	74
112	2.5	thin	0.374	0.414	68	3.1	17	67
112	2.5	thin	0.374	0.488	65	3.5	19	70
118	3.5	fair	0.164	0.532	67	4	23	66
118	3.75	fat	0.344	0.552	77	4.2	27	105
118	3.75	fat	0.492	0.507	50	4.5	26	82
118	3.75	fat	0.477	0.388	49	3.9	19	64
118	4	fat	0.333	0.435	64	4.2	25	62
118	4	fat	0.245	0.548	62	4.1	31	73
120	3.5	fair	0.146	0.714	83	4	20	70
120	2.75	thin	0.432	0.629	85	3.4	12	126
120	2.75	thin	0.317	0.614	88	3.5	17	102
120	2.75	thin	0.633	0.653	90	3.6	17	105
120	2.5	thin	0.457	0.306	67	3.7	17	87
120	2.5	thin	0.253	0.445	90	3.8	17	68

Cow	BCS	BCSg	NEFA	BHBA	GLU	Alb	Urea	AST
132	3.5	fair	0.236	0.354	78	3.6	18	60
132	3.5	fair	0.519	0.452	78	3.7	20	65
132	3.5	fair	0.287	0.467	90	3.8	18	63
132	3.5	fair	0.408	0.602	87	3.8	21	67
132	3	fair	0.215	0.432	78	3.8	25	62
132	3	fair	0.129	0.677	78	4	21	64
147	4.25	fat	0.239	0.423	87	4.3	26	51
147	3.5	fair	0.868	1.02	64	3.3	12	87
147	3.5	fair	0.595	0.691	76	3.7	13	86
147	3.5	fair	0.553	0.481	60	3.5	13	75
147	3	fair	0.438	0.632	61	3.3	16	59
147	3	fair	0.215	0.448	83	4.1	23	62
151	4	fat	0.107	0.544	73	3.9	22	86
151	4	fat	0.526	0.884	47	4.2	20	99
151	4	fat	0.445	0.705	47	4.2	25	73
151	4	fat	0.415	0.608	41	3.7	25	65
151	4.25	fat	0.658	0.78	52	4.1	21	76
151	4.25	fat	0.452	.	62	4.4	28	75
186	3.5	fair	0.256	0.605	97	4	25	77
186	2.75	thin	0.748	0.631	98	3.5	22	82
186	2.75	thin	0.639	0.638	92	3.3	20	79
186	2.75	thin	0.795	0.783	85	3.1	17	74
186	2.75	thin	0.517	0.523	98	3.3	12	91
186	2.75	thin	0.386	0.597	75	3.7	23	86
188	4.25	fat	0.188	0.399	70	3.7	21	58
188	4.25	fat	0.533	0.63	65	3.6	19	63
188	4.25	fat	1.4	0.519	56	3.5	21	70
188	4.25	fat	0.87	0.773	71	3.7	24	70
188	2.75	thin	0.932	0.835	72	3.8	19	72
188	2.75	thin	0.786	0.9	68	3.9	28	72
190	3.25	fair	0.173	0.32	72	4.1	22	68
190	3.5	fair	0.535	0.598	62	3.4	16	69
190	3.5	fair	0.333	0.357	73	3.7	19	71
190	3.5	fair	0.194	0.347	57	3.5	16	66
190	3	fair	0.208	0.483	45	3.6	22	74
190	3	fair	0.391	0.265	75	3.9	19	77
208	3.75	fat	0.305	0.453	77	4	26	58
208	2.75	thin	1.39	0.898	40	3.7	21	61
208	2.75	thin	2.07	1.83	32	3.7	22	67
208	2.75	thin	0.882	1.04	36	3.7	15	82
208	2.75	thin	0.575	0.661	59	3.9	17	92
208	2.75	thin	0.302	0.472	66	3.8	32	55
224	4.25	fat	0.132	0.471	71	3.6	20	58
224	4	fat	0.52	0.525	65	3.4	22	91
224	4	fat	0.44	0.743	77	3.8	20	89
224	4	fat	0.668	0.639	64	3.4	19	68
224	3.75	fat	0.491	0.759	69	3.6	18	97
224	3.75	fat	0.483	0.425	56	3.8	17	85

Cow	BCS	BCSg	NEFA	BHBA	GLU	Alb	Urea	AST
225	4	fat	0.206	0.36	43	4.1	29	52
225	2.75	thin	0.495	0.448	58	3.4	20	61
225	2.75	thin	0.683	0.548	59	4.1	21	71
225	2.75	thin	0.483	0.507	59	3.7	18	71
225	2.5	thin	0.908	0.625	67	4.3	21	72
225	2.5	thin	0.548	.	38	3.5	20	72
231	3.75	fat	0.115	0.384	60	4.1	24	74
231	4.25	fat	0.481	0.48	43	3.8	22	74
231	4.25	fat	0.75	0.653	48	4.2	25	73
231	4.25	fat	1.15	0.785	32	3.8	22	69
231	3.25	fair	1.03	1.27	43	4	19	79
231	3.25	fair	0.372	0.475	54	4.1	22	81
245	4	fat	0.115	0.598	61	3.6	23	63
245	3.75	fat	0.983	1.09	70	3.9	27	67
245	3.75	fat	0.768	0.978	82	3.6	25	68
245	3.75	fat	0.967	0.689	59	2.8	18	71
245	2.5	thin	1.1	1.72	50	3	17	105
245	2.5	thin	0.678	3.25	44	3.3	19	148
247	3.5	fair	0.156	0.406	54	4	27	57
247	3.5	fair	0.743	0.646	53	3.7	19	67
247	3.5	fair	0.688	0.592	44	3.7	16	86
247	3.5	fair	0.449	0.535	64	3.7	17	84
247	3.5	fair	0.359	0.541	43	3.5	18	72
247	3.5	fair	0.406	0.413	63	3.8	19	60
248	4.5	fat	0.358	0.462	76	4.3	24	57
248	3.75	fat	0.456	0.441	126	3.6	17	69
248	3.75	fat	0.441	0.415	99	3.7	16	61
248	3.75	fat	0.296	0.285	70	3	20	64
248	3.75	fat	0.408	0.507	88	3.3	19	80
248	3.75	fat	0.217	0.404	72	2.9	18	59
249	4	fat	0.091	0.381	61	3.8	26	58
249	2.75	thin	0.309	0.48	79	3.3	17	99
249	2.75	thin	0.501	0.441	101	3.1	14	100
249	2.75	thin	0.29	0.221	71	3.3	18	94
249	3.5	fair	0.372	0.536	62	3.5	16	69
249	3.5	fair	0.315	1.19	66	3.9	19	81
256	3.5	fair	0.373	0.469	75	3.9	30	67
256	2.25	thin	0.949	0.903	64	4.1	22	90
256	2.25	thin	0.689	0.784	55	4.3	28	84
256	2.25	thin	0.446	1.42	51	3.7	26	63
256	3.25	fair	0.831	1.32	55	4	29	68
256	3.25	fair	0.498	0.739	55	3.9	27	70
261	4.5	fat	0.087	0.412	51	3.5	26	55
261	3.75	fat	0.609	0.568	53	3.4	16	88
261	3.75	fat	0.412	0.342	80	4.1	27	85
261	3.75	fat	0.645	0.457	58	3.7	18	61
261	3.5	fair	0.661	0.578	57	4.1	18	62
261	3.5	fair	0.338	0.35	63	3.9	24	62

Cow	BCS	BCSg	NEFA	BHBA	GLU	Alb	Urea	AST
329	3	fair	0.103	0.388	70	3.6	21	56
329	2.25	thin	0.225	0.713	61	3.7	28	73
329	2.25	thin	0.632	0.775	45	3.7	26	64
329	2.25	thin	0.65	1.44	50	2.7	25	54
329	1.75	thin	0.489	0.624	45	2.2	16	145
329	1.75	thin	0.239	0.382	72	2.9	19	87
337	3	fair	0.121	0.459	63	4	22	80
337	3	fair	0.909	0.591	51	3.8	29	76
337	3	fair	0.98	0.73	73	3.9	28	78
337	3	fair	0.629	0.628	84	4	28	83
337	2.25	thin	0.342	0.578	75	4.3	19	92
337	2.25	thin	0.199	0.635	77	4.2	30	96
524	3.75	fat	0.177	0.499	74	3.6	17	56
524	2.25	thin	0.727	1.78	86	2.9	15	187
524	2.25	thin	0.662	1.04	76	2.9	15	177
524	2.25	thin	0.613	0.591	79	2.9	14	154
524	3	fair	0.41	0.799	70	3.1	16	130
524	3	fair	0.238	0.622	76	3.3	17	91
662	4	fat	0.243	0.488	58	3.9	31	71
662	3.5	fair	0.62	0.602	52	2.4	28	175
662	3.5	fair	0.344	0.369	65	2.8	20	139
662	3.5	fair	0.395	0.519	46	2.6	17	93
662	3.25	fair	0.338	0.428	65	3.1	18	82
662	3.25	fair	0.182	0.438	69	3.4	21	72
731	3.5	fair	0.096	0.485	61	4	24	79
731	2.5	thin	0.403	0.466	66	3.2	17	89
731	2.5	thin	0.709	0.614	62	2.9	16	76
731	2.5	thin	0.768	0.638	66	3.5	12	94
731	3.25	fair	0.45	0.539	73	3.5	18	95
731	3.25	fair	0.371	0.698	76	3.4	21	80
813	3.5	fair	0.404	0.45	89	3.7	21	79
813	3.5	fair	0.508	0.681	134	3.7	22	121
813	3.5	fair	0.944	0.675	97	3.4	18	83
813	3.5	fair	0.733	0.798	82	2.8	16	78
813	3.5	fair	0.637	0.494	108	2.9	15	96
813	2.75	thin	0.361	0.601	80	2.9	15	99
856	3.5	fair	0.207	0.703	50	4	21	57
856	3.25	fair	0.85	0.733	39	4	20	85
856	3.25	fair	0.594	0.834	42	3.6	18	83
856	3.25	fair	0.795	0.967	43	4.1	20	94
856	3.75	fat	0.882	0.979	32	4.1	21	92
856	3.75	fat	0.416	0.549	50	4	19	83
938	3.75	fat	0.141	0.408	61	3.8	23	52
938	3.5	fair	0.39	0.433	57	3.4	19	79
938	3.5	fair	0.496	0.481	65	3.9	22	71
938	3.5	fair	0.504	0.458	43	3.8	23	53
938	3.25	fair	0.318	0.543	58	4.2	21	64
938	3.25	fair	0.476	.	66	3.9	25	62

Cow	BCS	BCSg	NEFA	BHBA	GLU	Alb	Urea	AST
944	3.5	fair	0.16	0.546	51	4.1	25	53
944	4	fat	0.208	0.586	57	3.9	24	56
944	4	fat	0.333	0.668	34	4.4	27	67
944	4	fat	0.367	0.832	21	4	22	53
944	3.75	fat	0.572	0.849	43	4.2	20	68
944	3.75	fat	0.358	0.615	48	4.3	25	63
1281	3.75	fat	0.8	0.357	26	3.5	24	47
1281	4	fat	0.727	0.37	35	3.3	21	66
1281	4	fat	0.484	0.475	30	3.5	18	74
1281	4	fat	0.343	0.455	17	3.3	15	140
1281	3.25	fair	0.231	0.208	60	3	16	106
1281	3.25	fair	0.082	.	54	3	11	68
2493	3	fair	0.158	0.418	63	3.7	19	73
2493	4	fat	0.926	0.652	72	3.1	14	100
2493	4	fat	0.706	0.648	71	3.3	19	94
2493	4	fat	0.921	0.476	64	3.5	16	88
2493	2.75	thin	0.532	0.491	72	3.4	18	89
2493	2.75	thin	0.792	0.811	72	3.6	19	85
2624	3.25	fair	0.229	0.443	64	3.9	23	47
2624	3	fair	0.698	0.541	60	3.4	23	54
2624	3	fair	0.831	0.787	62	3.2	20	55
2624	3	fair	0.471	0.503	57	3.3	18	75
2624	2	thin	0.847	0.451	58	3	15	92
2624	2	thin	0.413	1.49	49	4.3	23	69
2694	3	fair	0.094	0.497	42	3.7	19	60
2694	2	thin	0.881	1.04	48	4	20	83
2694	2	thin	0.879	1.3	38	4	21	80
2694	2	thin	1.11	1.11	49	4.2	15	86
2694	2	thin	1.04	0.758	53	4.3	18	91
2694	2	thin	0.499	0.91	46	4.3	23	84
2741	3.75	fat	0.235	0.296	61	4	21	66
2741	3.75	fat	0.496	0.872	56	3.7	15	77
2741	3.75	fat	0.511	0.69	51	3.8	22	78
2741	3.75	fat	0.514	0.615	51	3.9	21	53
2741	3.75	fat	0.495	0.69	43	3.7	19	77
2741	3.75	fat	0.442	0.47	48	4	22	61
2764	3	fair	0.152	0.28	63	3.7	25	78
2764	2.75	thin	0.639	0.953	43	4.1	23	78
2764	2.75	thin	0.587	0.688	61	4.2	29	76
2764	2.75	thin	0.492	0.647	54	3.8	25	79
2764	2.75	thin	0.624	0.436	60	4.3	28	77
2764	2.75	thin	0.959	1.01	57	4.3	25	87
2813	3.25	fair	0.172	0.315	27	3.6	18	59
2813	2	thin	0.622	0.348	62	3.4	15	84
2813	2	thin	0.244	0.404	55	3.5	14	76
2813	2	thin	0.158	0.39	50	3.4	21	72
2813	1.75	thin	0.126	0.323	45	3.2	15	109
2813	1.75	thin	0.144	0.38	41	3.5	21	88

Cow	BCS	BCSg	NEFA	BHBA	GLU	Alb	Urea	AST
2940	3.5	fair	0.162	0.481	57	3.7	20	63
2940	3.5	fair	1.15	1.07	23	3.2	17	62
2940	3.5	fair	1.06	1.82	39	3.5	18	97
2940	3.5	fair	1.11	1.23	45	3.5	13	113
2940	2	thin	0.837	0.949	54	3.5	19	115
2940	2	thin	0.428	0.576	70	3.6	31	145
3526	3.5	fair	0.095	0.725	68	3.7	26	81
3526	3.25	fair	0.392	0.444	84	3.7	20	93
3526	3.25	fair	0.261	0.511	69	4.2	23	82
3526	3.25	fair	0.253	0.493	74	3.7	21	71
3526	2.75	thin	0.449	0.384	67	3.6	25	71
3526	2.75	thin	0.186	0.36	73	3.8	23	78
4019	3	fair	0.217	0.433	63	3.7	15	79
4019	2.75	thin	0.172	0.632	43	3.9	23	50
4019	2.75	thin	0.104	0.833	53	4.2	24	49
4019	2.75	thin	0.145	0.486	39	3.8	21	34
4019	2.75	thin	0.144	0.654	43	4	25	62
4019	2.75	thin	0.125	0.659	28	3.9	26	64
4044	3.5	fair	0.178	0.536	59	3.2	21	70
4044	3	fair	0.443	0.651	79	3.5	17	66
4044	3	fair	0.412	0.465	91	3.7	23	58
4044	3	fair	0.595	0.413	73	3.4	17	61
4044	3.25	fair	0.157	0.309	68	3	13	13
4044	3.25	fair	0.119	0.339	82	3.6	19	19
4053	3	fair	0.168	0.46	65	3.9	30	101
4053	3	fair	0.16	0.355	64	3.9	30	96
4053	3	fair	0.175	0.418	62	3.7	23	77
4053	3	fair	0.225	0.35	57	3.8	27	69
4053	2.5	thin	0.18	0.344	44	3.9	25	68
4053	2.5	thin	0.123	0.415	58	4	28	72
5061	3.5	fair	0.193	0.499	72	3.7	18	85
5061	3.5	fair	0.527	0.46	52	3.3	15	87
5061	3.5	fair	0.736	0.604	62	3.6	21	97
5061	3.5	fair	0.327	0.348	65	3	14	79
5061	3	fair	0.26	0.593	54	3.5	13	88
5061	3	fair	0.347	0.521	64	3.4	13	77
5104	3.25	fair	0.546	0.327	51	3.8	23	77
5104	1.75	thin	0.381	0.498	51	3	13	99
5104	1.75	thin	0.409	0.243	56	3.1	18	88
5104	1.75	thin	0.489	0.273	52	3	14	95
5104	1.5	thin	0.66	0.254	50	3.2	11	172
5104	1.5	thin	0.078	0.442	53	3.1	19	91
5165	3.75	fat	0.091	0.588	41	4	21	76
5165	3.75	fat	0.136	0.507	54	3.4	17	79
5165	3.75	fat	0.136	0.433	60	3.5	19	114
5165	3.75	fat	0.395	0.43	65	3.9	18	98
5165	4.25	fat	0.419	0.488	54	3.7	21	95
5165	4.25	fat	0.24	0.614	51	3.6	19	80

Cow	BCS	BCSg	NEFA	BHBA	GLU	Alb	Urea	AST
5170	3.75	fat	0.077	0.622	36	4	21	34
5170	2.75	thin	1.16	1.73	46	3.8	20	82
5170	2.75	thin	1.09	1.1	46	3.7	20	85
5170	2.75	thin	1.01	1.51	35	4	22	82
5170	2.5	thin	0.871	0.648	41	4	19	78
5170	2.5	thin	0.778	1.08	44	3.8	19	95
5254	3.5	fair	0.105	0.419	62	3.8	23	115
5254	3.5	fair	0.341	0.546	78	3.3	18	82
5254	3.5	fair	0.298	0.436	76	3.5	17	99
5254	3.5	fair	0.25	0.458	80	3.8	22	100
5254	2.25	thin	0.379	0.501	78	3.6	21	96
5254	2.25	thin	0.23	0.801	76	3.9	24	93
5371	4	fat	0.143	0.571	55	3.7	30	80
5371	2.75	thin	0.954	0.728	57	4.1	28	74
5371	2.75	thin	0.584	0.448	48	3.7	29	225
5371	2.75	thin	1.04	0.764	54	3.9	21	373
5371	3	fair	0.683	0.44	65	3.4	20	270
5371	3	fair	0.25	0.517	60	4	29	158
5397	3.5	fair	0.097	0.605	63	3.7	26	75
5397	3	fair	0.328	0.525	63	4.4	33	82
5397	3	fair	0.218	0.563	62	4.1	27	59
5397	3	fair	0.204	0.362	60	3.2	24	47
5397	2.5	thin	0.262	0.425	63	4.3	24	64
5397	2.5	thin	0.199	0.406	62	4.2	26	55
5405	3.5	fair	0.117	0.508	61	3.7	23	69
5405	3.25	fair	0.127	0.336	49	3.3	16	81
5405	3.25	fair	0.203	0.55	48	3.5	20	67
5405	3.25	fair	0.281	0.353	63	3.8	20	65
5405	2.5	thin	0.239	0.319	50	3.7	15	73
5405	2.5	thin	0.137	0.317	56	3.5	19	58
5415	3.75	fat	0.381	0.589	53	3.9	15	67
5415	3.25	fair	0.899	1.84	51	3.7	17	83
5415	3.25	fair	0.747	0.589	46	3.5	21	103
5415	3.25	fair	0.859	1.11	47	3.7	20	90
5415	2.25	thin	0.609	0.858	42	2.9	16	77
5415	2.25	thin	0.46	0.72	48	3.7	20	77
5771	3	fair	0.152	0.493	31	3.7	23	72
5771	3	fair	0.471	1.36	54	3.9	21	89
5771	3	fair	0.565	0.683	67	4	28	82
5771	3	fair	1.2	1.1	57	3.7	23	94
5771	2.75	thin	0.739	2.04	56	3.5	20	183
5771	2.75	thin	0.312	0.397	62	3.3	20	170
5919	2.75	thin	0.524	0.609	68	3.4	30	81
5919	2.75	thin	0.57	0.907	64	3.8	18	78
5919	2.75	thin	0.27	0.48	62	3.8	14	67
5919	2.75	thin	0.506	0.761	67	4.4	24	77
5919	2.5	thin	0.3	0.525	63	3.8	17	61
5919	2.5	thin	0.134	0.578	67	4.6	18	64

Cow	BCS	BCSg	NEFA	BHBA	GLU	Alb	Urea	AST
5971	3.25	fair	0.054	0.503	59	3.6	22	66
5971	3	fair	0.534	0.813	58	3.4	17	76
5971	3	fair	0.712	0.946	72	3.6	26	74
5971	3	fair	1.37	0.873	67	3.4	15	69
5971	2	thin	0.578	1.14	74	3.2	12	70
5971	2	thin	0.399	0.479	78	3.4	16	68
6052	3.75	fat	0.254	0.574	61	4.1	21	69
6052	3.5	fair	0.549	0.582	41	3.9	21	58
6052	3.5	fair	0.326	0.734	44	4.3	23	55
6052	3.5	fair	0.41	0.612	38	4	19	52
6052	3.75	fat	0.497	0.737	42	4.1	21	46
6052	3.75	fat	0.356	0.587	57	4.1	19	50
6123	3.5	fair	0.082	0.411	73	3.5	23	62
6123	3.75	fat	0.835	0.882	74	3.2	22	86
6123	3.75	fat	0.595	0.546	62	3.6	24	98
6123	3.75	fat	0.739	0.521	48	2.2	12	63
6123	3.25	fair	0.898	1.63	60	3.5	19	96
6123	3.25	fair	0.339	0.373	58	2.9	18	207
6145	3.5	fair	0.18	0.542	59	4.4	22	63
6145	3.25	fair	0.638	0.978	47	3.9	21	64
6145	3.25	fair	0.546	0.558	57	4.1	26	65
6145	3.25	fair	0.476	0.624	43	3.7	20	71
6145	2.25	thin	0.396	0.545	45	3.8	19	57
6145	2.25	thin	0.158	0.491	61	4	19	60
6203	3.75	fat	0.103	0.519	38	3.6	21	57
6203	3.25	fair	0.742	1.19	47	3.9	21	77
6203	3.25	fair	0.274	0.705	67	4.1	25	65
6203	3.25	fair	0.279	0.557	45	3.3	16	51
6203	3	fair	0.678	0.912	46	4	20	74
6203	3	fair	0.794	0.792	62	4.1	18	72
6220	3.5	fair	0.062	0.278	26	3	14	41
6220	2.75	thin	0.342	0.403	51	3.8	20	69
6220	2.75	thin	0.563	0.688	50	3.9	22	60
6220	2.75	thin	0.395	0.412	51	3.4	15	54
6220	3	fair	0.141	0.443	48	3.6	15	70
6220	3	fair	0.249	0.335	58	3.7	15	57
6284	3.75	fat	0.101	0.483	56	4.1	21	61
6284	3.5	fair	1.62	0.992	47	4.3	17	112
6284	3.5	fair	1.22	1.02	51	4.2	22	114
6284	3.5	fair	1.69	1.04	37	3.7	21	102
6284	3	fair	1.5	0.822	38	3.6	12	128
6284	3	fair	1.06	0.706	59	3.6	14	114
6515	3.5	fair	0.109	0.432	77	3.8	26	59
6515	2.75	thin	2.2	3.13	196	3.7	30	351
6515	2.75	thin	1.48	6.3	74	3.8	17	645
6515	2.75	thin	1.74	5.66	50	3.6	21	435
6515	1.75	thin	1.54	3.66	47	3.3	14	247
6515	1.75	thin	0.951	1.95	73	3.4	14	198

Cow	BCS	BCSg	NEFA	BHBA	GLU	Alb	Urea	AST
6540	3.75	fat	0.179	0.603	59	4	19	46
6540	3	fair	1.65	0.903	55	3.8	18	73
6540	3	fair	0.797	0.629	72	3.8	28	82
6540	3	fair	0.405	0.544	61	4.1	17	80
6540	2.25	thin	0.406	0.579	59	4.1	21	72
6540	2.25	thin	0.403	0.578	50	4.2	20	66
6724	3.5	fair	0.111	0.605	58	3.7	26	77
6724	3.75	fat	0.734	0.764	48	4.2	22	65
6724	3.75	fat	0.829	0.837	64	4.2	25	74
6724	3.75	fat	0.411	0.544	64	4.6	23	78
6724	3.5	fair	0.394	0.434	52	4.1	18	66
6724	3.5	fair	0.262	0.544	57	3.6	19	54
7048	3.5	fair	0.104	0.618	50	3.8	26	47
7048	3.25	fair	0.59	0.622	62	2.9	18	67
7048	3.25	fair	0.775	0.55	50	3	18	72
7048	3.25	fair	0.519	0.502	47	3.2	16	72
7048	2.75	thin	0.506	0.39	47	3.3	18	76
7048	2.75	thin	0.326	0.461	30	3.5	16	114
7050	4	fat	0.119	0.673	48	4	25	45
7050	3.5	fair	1.17	2.52	48	3.5	16	132
7050	3.5	fair	1.19	1.6	43	3.4	18	123
7050	3.5	fair	0.643	1.18	52	3.3	13	115
7050	3.25	fair	0.408	0.633	50	2.9	13	75
7050	3.25	fair	0.35	0.685	61	3.7	21	90
7067	3	fair	0.103	0.321	49	3.6	17	90
7067	3.5	fair	0.157	0.545	40	3.6	24	93
7067	3.5	fair	0.48	0.42	37	3.8	30	90
7067	3.5	fair	0.322	0.749	45	3.2	27	71
7067	3.25	fair	0.205	0.37	55	3.9	21	83
7067	3.25	fair	0.169	.	52	3.4	26	88
7068	4.5	fat	0.098	0.508	49	4	25	59
7068	4.25	fat	0.427	0.606	46	3.8	19	61
7068	4.25	fat	0.332	0.46	58	3.9	23	55
7068	4.25	fat	0.25	0.367	56	3.7	15	57
7068	4.25	fat	0.329	0.431	46	3.9	16	58
7068	4.25	fat	0.245	0.547	55	3.8	17	62
7113	4.25	fat	0.193	0.324	49	4.1	17	46
7113	3.75	fat	1.84	1.76	40	3.9	12	102
7113	3.75	fat	0.894	0.938	42	4.2	17	98
7113	3.75	fat	0.652	0.736	39	3.8	15	80
7113	3.75	fat	1.23	0.833	34	3.8	10	91
7113	3.75	fat	0.27	0.35	54	3.7	14	92
7121	4.25	fat	0.103	0.431	58	3.9	21	65
7121	3.5	fair	0.713	0.71	56	3.7	21	74
7121	3.5	fair	0.765	0.787	36	4.2	28	72
7121	3.5	fair	0.892	0.836	48	4	23	66
7121	3.5	fair	0.669	0.621	52	3.9	17	55
7121	3.5	fair	0.41	0.408	62	4.1	15	65

Cow	BCS	BCSg	NEFA	BHBA	GLU	Alb	Urea	AST
7133	3.75	fat	0.123	0.689	52	3.9	27	50
7133	2.75	thin	1.11	0.677	49	3.9	26	100
7133	2.75	thin	0.254	0.471	54	4.4	28	94
7133	2.75	thin	0.342	0.582	33	3.8	26	68
7133	2.5	thin	0.389	0.491	31	3.4	20	69
7133	2.5	thin	0.355	.	46	4.2	31	92
7136	3.5	fair	0.131	0.479	50	3.7	26	74
7136	3.25	fair	0.962	0.92	55	3.7	20	77
7136	3.25	fair	1.8	1.33	63	3.7	23	84
7136	3.25	fair	1.46	1.64	61	4	18	90
7136	2.25	thin	0.677	1.12	50	3.8	16	112
7136	2.25	thin	0.591	0.673	71	3	12	93
7140	4.25	fat	2.28	1.46	26	4.6	19	62
7140	2	thin	1.12	3.03	62	3.6	11	141
7140	2	thin	1.27	1.83	63	4	12	164
7140	2	thin	1.13	1.25	52	3.4	11	116
7140	.	.	1.27	1.92	40	3.5	13	141
7140	.	.	.	.	.	.	.	.
7141	4.25	fat	0.12	0.569	60	3.5	23	87
7141	3.75	fat	0.19	0.436	85	3.5	20	96
7141	3.75	fat	0.181	0.299	77	3.2	21	83
7141	3.75	fat	0.171	0.338	72	3.3	20	83
7141	4.25	fat	0.246	0.544	63	3.6	23	99
7141	4.25	fat	0.202	0.407	80	3.6	20	101
7151	3.5	fair	0.094	0.447	77	3.7	23	73
7151	2.5	thin	0.37	0.583	83	3.8	30	90
7151	2.5	thin	0.443	0.442	72	4.2	29	88
7151	2.5	thin	0.194	0.47	79	3.2	25	76
7151	2.25	thin	0.356	0.502	86	3.9	28	92
7151	2.25	thin	0.2	0.478	77	4.2	23	137
7157	3.25	fair	0.074	0.579	78	3.9	26	64
7157	2.5	thin	0.644	0.587	85	3.9	19	89
7157	2.5	thin	0.832	0.898	80	3.7	22	80
7157	2.5	thin	0.964	1.1	79	2.7	18	79
7157	2.5	thin	0.713	0.642	70	3.7	17	81
7157	2.5	thin	0.48	0.346	83	4	19	83
7177	3.5	fair	0.224	0.379	64	3.1	19	46
7177	3.75	fat	1.47	1.61	46	3.4	21	99
7177	3.75	fat	1.18	1.7	57	3.6	22	96
7177	3.75	fat	0.764	2.15	53	3.7	23	84
7177	3.5	fair	0.62	1.02	71	3.4	25	81
7177	3.5	fair	0.292	0.525	69	3.2	22	71
7194	4.5	fat	0.081	0.413	41	3.9	25	54
7194	3.75	fat	0.246	0.597	43	3.5	15	58
7194	3.75	fat	0.542	0.442	65	3.7	22	47
7194	3.75	fat	0.49	0.401	56	3.4	19	46
7194	3.5	fair	0.4	0.28	60	3.3	18	90
7194	3.5	fair	0.165	.	60	2.9	18	60

Cow	BCS	BCSg	NEFA	BHBA	GLU	Alb	Urea	AST
7268	3.75	fat	0.113	0.809	59	4.2	27	52
7268	3.25	fair	1.12	0.862	62	4	26	103
7268	3.25	fair	0.763	0.665	50	4.2	30	87
7268	3.25	fair	0.766	0.703	44	3.9	23	75
7268	3	fair	0.809	1.11	50	4.2	24	82
7268	3	fair	0.638	0.973	50	4.6	28	87
7322	4	fat	0.114	0.658	42	4	21	46
7322	4.5	fat	0.86	0.773	60	3.6	21	62
7322	4.5	fat	1.28	0.925	63	3.1	31	39
7322	4.5	fat	0.905	1.15	37	2.8	36	71
7322	3.25	fair	0.855	1.18	52	2.9	14	256
7322	3.25	fair	0.247	0.334	53	3.2	11	114
7353	4.5	fat	0.11	0.433	83	4.1	25	51
7353	3.5	fair	0.161	0.441	71	4.2	30	62
7353	3.5	fair	0.293	0.391	70	4.6	29	63
7353	3.5	fair	0.209	0.407	69	3.6	20	54
7353	3.5	fair	0.707	0.527	53	4	20	70
7353	3.5	fair	0.397	0.54	58	4	24	67
7368	3.25	fair	0.025	0.492	63	3.8	22	53
7368	3.25	fair	0.433	0.939	45	3.5	11	74
7368	3.25	fair	1.09	1.17	48	3.6	12	86
7368	3.25	fair	0.861	1.02	49	3.7	14	88
7368	2.25	thin	0.851	0.719	43	4	17	94
7368	2.25	thin	0.288	0.691	59	4.1	15	115
7403	3.5	fair	0.164	0.538	54	3.6	20	60
7403	2.75	thin	1.22	0.675	40	3.7	21	82
7403	2.75	thin	1.92	1.18	56	3.8	26	88
7403	2.75	thin	1.12	0.976	40	3.4	15	96
7403	2.75	thin	0.595	0.606	71	3.7	12	108
7403	2.75	thin	0.454	0.372	70	3.6	15	87
7412	3.25	fat	0.066	0.549	47	3.9	29	47
7412	3	fair	0.182	0.318	53	3.4	18	56
7412	3	fair	0.319	0.335	67	3.7	24	51
7412	3	fair	0.236	0.299	54	2.9	18	38
7412	2.75	thin	0.094	0.247	47	3.1	17	47
7412	2.75	thin	0.419	0.338	72	3.9	27	91
7521	3.75	fair	0.075	0.462	53	3.3	21	45
7521	2.75	thin	0.752	0.84	57	3.6	18	94
7521	2.75	thin	0.335	0.616	59	3.5	22	86
7521	2.75	thin	0.227	0.579	65	3.6	20	92
7521	3.25	fair	0.403	0.507	62	3.6	21	86
7521	3.25	fair	0.277	0.527	75	3.8	22	88
7537	4.75	fat	0.128	0.536	84	4	21	71
7537	4.5	fat	0.477	0.454	89	3.7	13	90
7537	4.5	fat	0.57	0.466	93	3.6	15	100
7537	4.5	fat	0.861	0.626	88	3.5	13	87
7537	4.25	fat	1.09	0.821	94	3.6	18	87
7537	4.25	fat	0.641	0.747	97	3.6	18	79

Cow	BCS	BCSg	NEFA	BHBA	GLU	Alb	Urea	AST
7543	4.25	fat	0.129	0.533	47	4.2	29	48
7543	2.75	thin	0.51	0.416	80	3.7	32	73
7543	2.75	thin	0.657	0.949	62	3.5	34	54
7543	2.75	thin	0.777	0.654	63	2.7	17	48
7543	2.25	thin	0.448	1.04	52	2.7	16	54
7543	2.25	thin	0.33	0.737	38	3.3	17	68
7550	4.25	fat	0.127	0.388	61	3.9	24	52
7550	4.5	fat	0.929	0.699	46	3.7	20	71
7550	4.5	fat	0.971	0.907	48	3.5	15	67
7550	4.5	fat	0.759	0.745	56	3.4	16	78
7550	3.25	fair	0.88	0.896	44	3.9	17	89
7550	3.25	fair	0.479	0.391	63	4.2	19	73
7712	3.75	fat	0.103	0.282	55	3.4	13	43
7712	3.75	fat	0.21	0.513	55	4	17	66
7712	3.75	fat	0.628	0.713	55	4	15	66
7712	3.75	fat	0.42	0.478	61	4.1	22	57
7712	3.75	fat	0.187	0.394	66	4.1	21	58
7712	3.75	fat	0.299	.	74	4.4	19	43
8015	3.25	fair	0.069	0.228	29	4	20	69
8015	2.75	thin	0.44	0.347	54	3.7	18	99
8015	2.75	thin	0.646	0.521	49	3.9	17	102
8015	2.75	thin	0.403	0.505	51	3.6	18	94
8015	2.25	thin	0.574	0.276	50	3.2	12	78
8015	2.25	thin	0.472	0.671	53	3.9	18	96
8017	3	fair	0.073	0.39	64	3.9	19	78
8017	3	fair	0.442	0.662	60	3.9	18	71
8017	3	fair	0.329	0.716	56	3.9	19	130
8017	3	fair	0.953	0.577	56	3.9	19	160
8017	2.5	thin	0.41	0.571	59	3.9	16	105
8017	2.5	thin	0.195	0.823	57	3.9	19	80
8027	3.25	fair	0.075	0.591	59	3.7	23	64
8027	3.25	fair	0.249	0.38	68	3.6	16	89
8027	3.25	fair	0.336	0.579	74	3.5	25	78
8027	3.25	fair	0.453	0.403	77	3.6	22	76
8027	2.25	thin	0.601	0.566	64	3.9	24	79
8027	2.25	thin	0.351	0.488	57	3.8	24	84
8040	3.75	fat	0.07	0.68	71	3.7	26	62
8040	3.75	fat	0.331	0.567	94	3.5	16	188
8040	3.75	fat	0.246	0.522	87	3.8	26	172
8040	3.75	fat	0.33	0.588	89	3.3	20	97
8040	2.75	thin	0.725	0.737	71	3.4	19	86
8040	2.75	thin	0.352	0.369	93	3.6	16	84
8046	3.25	fair	0.133	0.508	47	3.4	17	48
8046	3.25	fair	1.12	1.15	48	3.3	19	65
8046	3.25	fair	0.487	0.663	56	2.9	17	51
8046	3.25	fair	0.832	1.75	42	3.3	22	67
8046	2	thin	0.702	1.9	226	2.4	12	76
8046	2	thin	0.36	0.646	54	3	13	96

Cow	BCS	BCSg	NEFA	BHBA	GLU	Alb	Urea	AST
8047	3.5	fair	0.228	0.329	70	3.9	20	32
8047	3.5	fair	0.791	0.641	147	3.8	18	71
8047	3.5	fair	0.446	0.55	69	3.8	14	68
8047	3.5	fair	0.192	0.71	67	3.6	13	65
8047	3.25	fair	0.191	0.529	67	3.9	19	77
8047	3.25	fair	0.159	0.429	88	4.1	18	84
8082	4.25	fat	0.083	0.504	60	4	36	62
8082	3.75	fat	0.501	0.6	56	4.1	30	78
8082	3.75	fat	0.491	0.421	61	4.2	32	80
8082	3.75	fat	0.319	0.6	68	4	35	75
8082	3.5	fair	0.67	0.45	59	3.7	29	68
8082	3.5	fair	0.434	0.541	73	4.5	31	89
8086	3.5	fair	0.087	0.393	39	3	26	46
8086	2.5	thin	0.592	0.538	42	3.6	22	69
8086	2.5	thin	0.564	0.774	43	3.5	20	73
8086	2.5	thin	0.541	0.453	35	3.5	22	68
8086	3	fair	0.35	0.453	27	3.5	19	67
8086	3	fair	0.25	0.432	51	3.7	22	83
8122	3.5	fair	0.05	0.549	25	2.6	15	47
8122	2.75	thin	0.49	0.521	59	3.6	17	82
8122	2.75	thin	0.431	0.549	51	3.8	17	88
8122	2.75	thin	0.335	0.503	57	3.7	17	82
8122	3	fair	0.419	0.433	57	3.6	17	85
8122	3	fair	0.314	0.663	62	3.5	12	80
8129	3.25	fair	0.106	0.573	53	3.7	25	50
8129	2.25	thin	0.623	1.2	40	4.2	19	77
8129	2.25	thin	1.32	1.12	41	4.1	26	72
8129	2.25	thin	0.777	0.831	58	3.9	13	98
8129	2.25	thin	0.345	0.5	57	3.5	10	78
8129	2.25	thin	0.694	0.791	70	4.4	24	88
8133	3.75	fat	0.187	0.382	54	3.9	17	84
8133	3.25	fair	1.21	1.72	33	4	15	94
8133	3.25	fair	0.93	1.03	62	4.4	26	84
8133	3.25	fair	0.955	1.11	49	4.1	18	74
8133	3.25	fair	1.24	1.43	44	4.2	18	56
8133	3.25	fair	0.326	0.599	58	4.1	14	77
8152	3.5	fair	0.152	0.543	74	4	24	72
8152	4.25	fat	1.83	3.65	36	4	25	209
8152	4.25	fat	0.566	1.89	58	4.6	36	179
8152	4.25	fat	1.02	1.38	45	4	23	117
8152	2.5	thin	1.19	2.16	47	4.4	24	107
8152	2.5	thin	0.454	.	55	3.6	19	87
8154	4	fat	0.099	0.503	55	4.2	21	43
8154	4	fat	0.546	1.22	47	4.2	20	81
8154	4	fat	0.72	0.602	55	4.6	25	62
8154	4	fat	0.616	0.815	42	4	19	51
8154	4.25	fat	1.12	0.899	62	4.4	37	61
8154	4.25	fat	0.388	0.501	67	4.4	20	56

Cow	BCS	BCSg	NEFA	BHBA	GLU	Alb	Urea	AST
8156	3.25	fair	0.149	0.617	81	4.1	24	43
8156	3	fair	0.548	0.746	85	3.6	21	84
8156	3	fair	0.739	1.1	79	3.2	20	60
8156	3	fair	1.72	1.01	72	3.2	16	93
8156	2.75	thin	0.876	1.08	80	3.2	18	113
8156	2.75	thin	0.793	1.02	81	3.2	13	99
8158	3	fair	0.4	0.732	58	3.8	36	53
8158	3	fair	0.689	0.677	40	3.9	25	86
8158	3	fair	0.636	0.69	38	4.3	35	86
8158	3	fair	0.328	0.54	38	3.7	26	69
8158	3.5	fair	0.298	0.577	58	3.9	23	78
8158	3.5	fair	0.223	0.641	51	3.8	29	76
8160	4	fat	0.088	0.425	66	3.9	22	67
8160	3	fair	0.271	0.466	43	3.7	15	67
8160	3	fair	0.337	0.375	59	4.1	22	68
8160	3	fair	0.615	0.539	38	3.7	17	63
8160	2.25	thin	0.444	0.529	56	3.8	19	64
8160	2.25	thin	0.284	0.468	57	4	19	63
8177	3.75	fat	0.9	0.427	56	4.1	28	60
8177	3.75	fat	1.08	0.995	52	3.9	24	83
8177	3.75	fat	0.881	0.913	50	4.4	32	79
8177	3.75	fat	0.605	1.15	44	3.8	23	66
8177	2.25	thin	0.983	1.68	47	4.1	24	82
8177	2.25	thin	0.233	0.43	65	4.2	21	81
8181	3	fair	0.102	0.417	55	3.9	23	80
8181	3	fair	0.097	0.395	36	3.6	23	60
8181	3	fair	0.11	0.502	40	3.9	22	58
8181	3	fair	0.132	0.362	44	3.7	19	65
8181	3.25	fair	0.24	0.488	34	3.9	17	81
8181	3.25	fair	0.197	0.528	53	4.4	19	76
8186	3.25	fair	0.106	0.446	60	3.9	19	52
8186	3	fair	0.693	0.798	47	3.8	16	80
8186	3	fair	0.864	0.652	52	3.8	16	74
8186	3	fair	0.747	0.731	59	4	18	80
8186	2.25	thin	0.666	0.522	69	4	13	73
8186	2.25	thin	0.4	0.759	50	3.5	19	59
8221	3.75	fat	0.138	0.483	65	4.1	26	43
8221	3.25	fair	0.539	0.491	65	3.8	20	53
8221	3.25	fair	0.799	0.723	60	4	20	68
8221	3.25	fair	1.75	1.01	58	4.1	25	56
8221	3.25	fair	0.907	0.887	58	4	20	59
8221	3.25	fair	0.53	0.623	69	4.5	27	67
8230	3.5	fair	0.078	0.306	42	3.5	23	43
8230	4	fat	0.349	0.49	50	3.7	25	83
8230	4	fat	0.582	0.496	55	3.8	23	96
8230	4	fat	0.519	0.557	39	3.9	25	93
8230	3.25	fair	0.577	0.547	52	4.2	25	100
8230	3.25	fair	0.41	.	60	3.9	28	74

Cow	BCS	BCSg	NEFA	BHBA	GLU	Alb	Urea	AST
8244	3.25	fair	0.051	0.404	33	3.8	29	46
8244	3.25	fair	0.625	0.634	41	4	24	76
8244	3.25	fair	0.383	0.573	32	4	22	79
8244	3.25	fair	0.352	0.464	39	4	17	69
8244	3.25	fair	0.296	0.607	30	2.9	18	51
8244	3.25	fair	0.268	.	32	4.1	23	58
8252	3.25	fair	0.069	0.39	64	3.9	30	61
8252	3.25	fair	0.525	0.549	67	4	27	78
8252	3.25	fair	0.951	0.805	36	3.4	19	80
8252	3.25	fair	0.439	0.379	50	3.9	25	62
8252	2.25	thin	0.595	0.416	44	3.8	21	60
8252	2.25	thin	0.609	0.464	60	4.1	24	66
8268	4	fat	0.101	0.42	42	4.1	24	53
8268	3.5	fair	0.828	1.25	45	4.1	23	95
8268	3.5	fair	0.946	0.752	51	3.8	25	90
8268	3.5	fair	0.602	0.838	40	3.8	18	81
8268	3.5	fair	0.933	0.651	54	4.2	19	80
8268	3.5	fair	0.883	0.569	49	3.6	23	65
8313	3.5	fair	0.179	0.401	34	3.9	22	43
8313	2.5	thin	0.399	0.629	59	3.3	16	45
8313	2.5	thin	0.516	0.513	45	3.2	17	52
8313	2.5	thin	0.51	0.479	34	2.6	15	45
8313	2.25	thin	0.587	0.686	46	3.5	16	79
8313	2.25	thin	1.02	.	27	3.3	15	62
8339	3.5	fair	0.106	0.606	52	3.9	24	51
8339	3.5	fair	0.992	1.16	30	3.3	22	123
8339	3.5	fair	0.811	0.757	36	4	27	124
8339	3.5	fair	0.745	1.21	38	3.7	24	108
8339	3.5	fair	0.609	0.938	53	3.6	18	103
8339	3.5	fair	0.392	1.04	47	3	18	65
8372	3.25	fair	0.074	0.439	44	4	29	63
8372	4	fat	0.495	0.511	33	4	24	87
8372	4	fat	0.546	0.507	41	4.4	32	78
8372	4	fat	0.439	0.501	35	4.1	24	69
8372	2.25	thin	0.626	0.584	53	3.9	21	72
8372	2.25	thin	0.842	0.508	63	3.7	24	135
8375	3.25	fair	0.384	0.597	51	4	19	55
8375	3	fair	1.5	2.59	39	3.5	16	131
8375	3	fair	0.648	2.91	44	3.1	13	145
8375	3	fair	0.64	0.945	57	3	13	137
8375	2.25	thin	0.488	0.471	52	3.1	16	102
8375	2.25	thin	0.291	0.665	56	3.5	22	103
8376	3.25	fair	0.107	0.649	55	3.9	24	80
8376	2	thin	1.06	1.14	56	4	23	80
8376	2	thin	0.598	0.992	52	3.5	18	73
8376	2	thin	0.523	0.85	60	3.8	18	73
8376	2	thin	0.725	0.63	58	3.9	20	74
8376	2	thin	0.259	0.606	44	4	21	77

Cow	BCS	BCSg	NEFA	BHBA	GLU	Alb	Urea	AST
8389	3.75	fat	0.108	0.676	54	4	29	42
8389	4.25	fat	0.802	0.756	46	4.2	27	64
8389	4.25	fat	0.667	0.728	41	4.2	27	51
8389	4.25	fat	1.31	0.779	48	4.1	22	51
8389	3.75	fat	0.711	0.822	45	3.5	17	47
8389	3.75	fat	0.291	0.65	62	4.1	26	57
8393	3.25	fair	0.131	0.397	54	4.1	23	59
8393	2.5	thin	0.636	0.76	55	3.6	17	82
8393	2.5	thin	1.22	0.795	46	4.3	26	77
8393	2.5	thin	1.9	2.7	26	3.7	24	69
8393	1.75	thin	0.81	1.73	35	4	20	100
8393	1.75	thin	0.309	0.535	45	3.5	15	74
8398	3	fair	0.15	0.462	70	3.4	23	77
8398	2	thin	0.81	1.06	55	3.6	15	125
8398	2	thin	0.455	0.784	71	3.4	17	107
8398	2	thin	0.693	1.03	54	3.4	13	97
8398	2	thin	0.828	0.728	62	3.6	17	91
8398	2	thin	0.665	0.582	61	3.5	16	115
8401	3.5	fair	0.093	0.696	72	4	28	47
8401	3.5	fair	0.272	0.803	60	4.2	21	68
8401	3.5	fair	0.64	0.624	68	4	25	52
8401	3.5	fair	0.231	0.599	43	4.2	18	57
8401	3.25	fair	0.253	0.555	57	4.3	19	55
8401	3.25	fair	0.084	.	74	4.2	24	60
8402	3.75	fat	0.144	0.438	52	3.7	19	56
8402	3.5	fair	0.44	0.535	65	3.9	18	85
8402	3.5	fair	0.659	0.602	57	3.8	22	71
8402	3.5	fair	0.902	0.719	60	3.8	18	65
8402	3.25	fair	0.686	0.652	66	3.8	19	69
8402	3.25	fair	0.296	0.622	73	4.2	23	81
8414	3.5	fair	0.124	0.423	74	4.1	21	51
8414	2.75	thin	0.423	0.502	86	3.7	21	80
8414	2.75	thin	0.385	0.363	352	3.9	23	66
8414	2.75	thin	0.357	0.263	72	3.9	22	75
8414	2.75	thin	0.495	0.403	68	3.7	21	73
8414	2.75	thin	0.139	0.343	68	3.9	23	73
8469	3.5	fair	0.071	0.653	74	3.8	36	69
8469	2.5	thin	0.793	0.579	71	3.9	22	76
8469	2.5	thin	0.574	0.56	93	4	30	71
8469	2.5	thin	0.524	0.536	74	3.8	24	81
8469	2.5	thin	0.701	1.13	66	3.9	25	94
8469	2.5	thin	0.372	0.389	77	4.1	17	90
8510	3.5	fair	0.091	0.505	64	3.7	29	48
8510	3.5	fair	0.505	0.474	54	3.6	19	82
8510	3.5	fair	0.507	0.431	73	3.9	24	79
8510	3.5	fair	0.297	0.423	47	3.4	22	77
8510	2.75	thin	0.305	0.31	59	4.2	26	72
8510	2.75	thin	0.246	0.485	76	4.3	27	79

Cow	BCS	BCSg	NEFA	BHBA	GLU	Alb	Urea	AST
8522	3.5	fair	0.086	0.591	39	3.6	27	44
8522	3.25	fair	0.691	0.919	46	3.6	23	86
8522	3.25	fair	0.404	0.415	66	4.3	30	72
8522	3.25	fair	0.55	0.909	48	4	21	85
8522	3.25	fair	0.649	0.628	57	4.1	21	82
8522	3.25	fair	0.427	0.423	51	4.1	21	77
8599	4.25	fat	0.116	0.431	57	4.2	24	71
8599	3.5	fair	0.384	0.425	68	3.4	16	142
8599	3.5	fair	0.31	0.358	71	3.8	19	79
8599	3.5	fair	0.342	0.456	57	3.7	17	68
8599	3.5	fair	0.187	0.483	71	3.8	22	77
8599	3.5	fair	0.133	.	71	4	17	71
8684	3	fair	0.097	0.538	62	4	24	62
8684	2.75	thin	0.582	0.627	60	3.4	23	75
8684	2.75	thin	0.318	0.889	86	3.7	20	86
8684	2.75	thin	0.711	0.777	69	4	22	84
8684	2.25	thin	0.63	0.545	73	4.2	22	74
8684	2.25	thin	0.778	0.753	78	4.1	21	73
8704	4	fat	0.132	0.634	46	4.2	24	56
8704	4.25	fat	0.969	0.979	24	4	21	61
8704	4.25	fat	1.36	1.26	53	3.8	20	68
8704	4.25	fat	0.835	1.17	53	3.6	17	81
8704	3.25	fair	0.67	0.833	60	4.1	18	106
8704	3.25	fair	0.266	0.671	60	3.7	19	70
8706	3.25	fair	0.031	0.372	49	3.6	23	66
8706	2.5	thin	0.394	0.551	41	3.4	20	91
8706	2.5	thin	0.5	0.606	60	3.7	18	91
8706	2.5	thin	0.946	0.67	45	3.6	18	83
8706	3.25	fair	0.497	0.562	29	3.3	19	78
8706	3.25	fair	0.337	.	42	3.2	14	67
9004	2.75	thin	0.072	0.39	35	3.1	15	67
9004	2.25	thin	0.415	0.731	49	3.7	18	81
9004	2.25	thin	0.622	0.476	48	3.8	16	89
9004	2.25	thin	0.37	0.424	47	3.8	14	85
9004	3	fair	0.186	0.518	46	3.7	20	74
9004	3	fair	0.194	0.386	60	4	18	72
9027	3.25	fair	0.073	0.597	46	3.6	23	59
9027	3	fair	0.119	0.549	49	4.3	31	85
9027	3	fair	0.093	0.693	67	4.1	25	70
9027	3	fair	0.119	0.575	72	3.9	26	65
9027	3	fair	0.162	0.525	68	4	23	71
9027	3	fair	0.176	0.444	45	3.4	19	68
9028	3	fair	0.079	0.517	42	3.8	18	74
9028	2.25	thin	0.427	0.607	46	3.6	19	84
9028	2.25	thin	0.292	0.381	35	3.6	17	81
9028	2.25	thin	0.378	0.484	44	3.9	17	75
9028	2.25	thin	0.257	0.507	41	3.5	16	77
9028	2.25	thin	0.333	0.507	57	4.1	17	179

Cow	BCS	BCSg	NEFA	BHBA	GLU	Alb	Urea	AST
9030	3.75	fat	0.077	0.616	43	3.7	22	61
9030	3.75	fat	0.31	0.399	40	3.2	14	69
9030	3.75	fat	0.286	0.439	34	3.9	22	70
9030	3.75	fat	0.359	0.465	51	3.9	19	68
9030	3.5	fair	0.633	0.6	51	3.8	18	70
9030	3.5	fair	0.537	0.526	39	4.2	21	75
9031	4	fat	0.089	0.391	47	4.4	37	56
9031	2.5	thin	0.99	1.02	32	3.7	30	119
9031	2.5	thin	0.488	0.721	41	3.9	26	115
9031	2.5	thin	0.944	0.5	36	3.4	20	80
9031	2.75	thin	0.333	0.44	50	4.1	23	89
9031	2.75	thin	0.3	0.458	63	4.1	23	108
9060	3.25	fair	0.137	0.709	58	3.5	20	80
9060	3.25	fair	0.346	0.908	40	3.2	20	74
9060	3.25	fair	0.254	0.666	45	3.6	22	77
9060	3.25	fair	0.333	0.576	57	3.3	19	67
9060	2.25	thin	0.731	1.15	33	3.6	22	74
9060	2.25	thin	0.231	0.465	45	3.8	20	84
9066	3.25	fair	0.077	0.482	72	3.4	20	70
9066	3.5	fair	0.238	0.569	84	3.8	25	93
9066	3.5	fair	0.579	0.67	83	3.5	25	80
9066	3.5	fair	0.509	0.823	83	3.7	20	84
9066	3	fair	1.16	0.637	85	3.9	20	89
9066	3	fair	0.459	0.67	64	3.8	22	104
9074	3.75	fat	0.095	0.587	47	4	22	65
9074	3.75	fat	0.464	0.46	51	3.9	16	90
9074	3.75	fat	0.531	0.43	66	4	20	80
9074	3.75	fat	0.19	0.358	54	3.3	12	61
9074	3.5	fair	0.418	0.449	68	4.2	17	81
9074	3.5	fair	0.445	0.533	76	4.2	18	108
9087	4	fat	0.19	0.473	37	3.8	19	62
9087	4	fat	0.44	0.513	78	3.7	17	76
9087	4	fat	0.481	0.28	64	3.4	13	75
9087	4	fat	0.607	0.548	68	3.4	13	73
9087	3.25	fair	0.59	0.476	54	3.4	11	85
9087	3.25	fair	0.246	0.384	69	3.3	9	128
9092	3	fair	0.091	0.624	53	3.9	31	69
9092	3	fair	0.886	0.498	49	4.1	22	59
9092	3	fair	0.447	0.559	39	4.2	24	50
9092	3	fair	0.851	0.66	49	3.8	16	58
9092	3	fair	0.335	0.546	58	3.8	17	58
9092	3	fair	0.391	0.389	63	4.2	23	64
9101	3.25	fair	0.098	0.488	34	3.6	23	91
9101	3.5	fair	0.254	0.367	22	3.4	22	87
9101	3.5	fair	0.305	0.474	35	4.2	26	89
9101	3.5	fair	0.27	0.433	30	3.6	28	85
9101	3.5	fair	0.24	0.561	34	4.1	29	78
9101	3.5	fair	0.148	.	45	4	37	70

Cow	BCS	BCSg	NEFA	BHBA	GLU	Alb	Urea	AST
9112	3.25	fair	0.128	0.596	64	3.9	28	53
9112	2.5	thin	0.75	0.819	47	3.6	19	57
9112	2.5	thin	0.764	1.12	53	4	23	75
9112	2.5	thin	0.859	0.929	67	4.2	26	71
9112	2.25	thin	0.441	1.06	57	4.1	21	69
9112	2.25	thin	0.188	0.635	68	4.4	29	58
9123	3.25	fair	0.14	0.595	43	4.4	25	87
9123	3.5	fair	0.359	0.411	52	3.7	16	73
9123	3.5	fair	0.247	0.459	58	4.1	25	66
9123	3.5	fair	0.273	0.415	37	3.7	24	62
9123	3.5	fair	0.297	0.476	54	3.9	17	60
9123	3.5	fair	0.211	.	59	4.2	22	63
9128	3	fair	0.221	0.417	52	3.7	23	62
9128	2.5	thin	0.335	0.456	48	3.9	27	66
9128	2.5	thin	0.421	0.433	50	3.9	25	69
9128	2.5	thin	0.265	0.413	46	4.2	27	70
9128	3.25	fair	0.177	0.459	62	4.2	27	73
9128	3.25	fair	0.226	0.433	56	3.9	25	61
9132	3.25	fair	0.082	0.491	29	3.8	23	78
9132	2.25	thin	0.401	0.644	49	3.8	26	84
9132	2.25	thin	0.357	0.602	52	4.2	32	74
9132	2.25	thin	0.363	0.611	47	3.6	20	85
9132	2	thin	0.17	0.598	41	3.8	29	97
9132	2	thin	0.176	0.483	49	4	21	97
9141	3.75	fat	0.1	0.625	59	4.1	30	54
9141	3.25	fair	0.478	0.442	41	3.9	23	81
9141	3.25	fair	0.75	0.492	55	3.9	22	76
9141	3.25	fair	0.546	0.516	59	4.1	22	72
9141	3.25	fair	0.662	0.58	52	4.3	22	87
9141	3.25	fair	0.442	0.476	66	4.4	23	89
9147	2.75	thin	0.107	0.542	46	3.9	24	46
9147	3.5	fair	0.279	0.537	64	3.5	19	48
9147	3.5	fair	0.582	0.552	70	4.1	23	56
9147	3.5	fair	0.309	0.41	54	3.6	21	45
9147	3.25	fair	0.275	0.485	67	4	20	41
9147	3.25	fair	0.323	.	67	4	22	55
9160	2.75	thin	0.089	0.645	59	3.6	26	63
9160	2.5	thin	0.245	0.593	53	3.8	23	86
9160	2.5	thin	0.556	0.774	63	4.2	29	75
9160	2.5	thin	0.521	0.685	53	3.9	21	74
9160	2.5	thin	0.299	0.536	53	3.9	20	84
9160	2.5	thin	0.177	0.559	59	4.1	22	90
9166	3	fair	0.122	0.492	51	3.7	23	46
9166	3	fair	0.311	0.576	52	4	22	70
9166	3	fair	0.397	0.573	61	3.9	21	59
9166	3	fair	0.639	0.514	54	3.8	20	59
9166	2.25	thin	0.662	0.525	75	4.1	19	86
9166	2.25	thin	0.762	0.632	49	4.2	22	76

Cow	BCS	BCSg	NEFA	BHBA	GLU	Alb	Urea	AST
9167	3	fair	0.116	0.594	52	4	24	65
9167	3.25	fair	0.678	0.553	57	4.1	20	75
9167	3.25	fair	0.367	0.541	45	4.2	18	76
9167	3.25	fair	0.444	0.453	53	4.2	17	64
9167	3.25	fair	0.3	0.601	41	4.3	19	77
9167	3.25	fair	0.137	0.502	50	3.8	14	65
9168	3.25	fair	0.187	0.591	46	3.8	28	57
9168	3	fair	0.261	0.541	49	3.6	23	62
9168	3	fair	0.613	0.704	51	4.2	24	76
9168	3	fair	0.517	0.666	39	3.9	23	72
9168	2.25	thin	0.858	0.581	54	3.8	22	68
9168	2.25	thin	0.95	0.689	49	3.8	21	64
9178	3	fair	0.103	0.681	40	3.7	25	46
9178	3.25	fair	0.187	0.485	68	3.8	18	72
9178	3.25	fair	0.521	0.393	62	3.9	23	60
9178	3.25	fair	0.131	0.488	65	3.9	26	72
9178	2.5	thin	0.441	0.261	68	3.6	21	76
9178	2.5	thin	0.293	0.418	71	4.3	23	76
9181	3.75	fat	0.132	0.457	49	4.2	28	55
9181	3	fair	0.512	0.65	60	4	22	71
9181	3	fair	0.407	0.386	71	4.6	31	66
9181	3	fair	0.281	0.485	51	4.1	25	53
9181	3.5	fair	0.22	0.298	58	3.7	21	51
9181	3.5	fair	0.136	.	73	4.3	24	73
9194	2.5	thin	0.107	0.717	44	3.8	25	91
9194	2.5	thin	0.45	0.499	52	4	22	113
9194	2.5	thin	0.496	0.543	56	4.3	30	100
9194	2.5	thin	0.219	0.686	37	3.7	24	68
9194	2	thin	0.217	0.583	56	3.7	20	52
9194	2	thin	0.258	0.444	47	4.1	24	57
9204	3	fair	0.077	0.499	46	4	31	43
9204	3.25	fair	0.578	0.558	19	3.7	21	61
9204	3.25	fair	0.636	0.413	35	4.2	26	62
9204	3.25	fair	0.509	0.906	23	4.2	28	64
9204	3	fair	0.245	0.5	37	3.4	22	67
9204	3	fair	0.517	0.379	42	4.2	24	64
9210	3.25	fair	0.087	0.55	49	3.8	27	55
9210	3.25	fair	0.639	0.544	57	4.1	23	78
9210	3.25	fair	0.612	0.593	48	4.3	28	64
9210	3.25	fair	0.267	0.463	42	3.7	20	60
9210	2.75	thin	0.413	0.614	46	3.8	20	64
9210	2.75	thin	0.176	0.403	58	4	19	69
9211	3.25	fair	0.108	0.562	60	3.8	24	52
9211	3.25	fair	0.262	0.476	42	3.6	14	67
9211	3.25	fair	0.173	0.463	48	3.6	14	82
9211	3.25	fair	0.27	0.392	59	3.6	16	75
9211	3	fair	0.316	0.39	62	3.5	19	75
9211	3	fair	0.116	0.545	69	3.9	19	72

Cow	BCS	BCSg	NEFA	BHBA	GLU	Alb	Urea	AST
9218	3	fair	0.082	0.494	63	3.5	27	74
9218	3.25	fair	0.076	0.485	57	3.6	20	87
9218	3.25	fair	0.177	0.45	58	3.9	24	77
9218	3.25	fair	0.267	0.359	54	3.6	19	74
9218	3.25	fair	0.226	0.428	72	3.7	21	82
9218	3.25	fair	0.125	0.364	63	3.9	24	74
9226	3.5	fair	0.491	0.276	60	3.9	23	69
9226	3.25	fair	0.405	0.61	89	3.7	16	105
9226	3.25	fair	0.154	0.442	81	3.6	18	99
9226	3.25	fair	0.099	0.264	86	3.5	15	85
9226	3.25	fair	0.557	0.311	92	3.9	18	85
9226	3.25	fair	0.568	0.341	93	3.8	26	75
9228	3	fair	0.096	0.698	72	3.8	29	69
9228	3	fair	0.695	0.757	71	3.7	21	83
9228	3	fair	0.472	0.497	91	3.9	21	68
9228	3	fair	0.507	0.457	76	3.6	19	69
9228	2.5	thin	0.644	0.434	69	3.8	17	76
9228	2.5	thin	0.475	0.358	82	3.7	20	90
9229	3.5	fair	0.217	0.607	69	3.8	26	55
9229	3.25	fair	0.724	0.511	68	3.9	24	67
9229	3.25	fair	0.787	0.615	55	4.1	25	64
9229	3.25	fair	0.53	0.454	68	3.6	19	64
9229	3.5	fair	0.551	0.624	74	3.9	19	71
9229	3.5	fair	0.373	0.547	75	4.2	24	70
9290	3	fair	0.126	0.383	40	3.8	24	85
9290	2.5	thin	0.35	0.624	39	3.6	21	93
9290	2.5	thin	0.368	0.709	35	3.8	25	71
9290	2.5	thin	0.384	0.658	52	3.8	18	71
9290	3.25	fair	0.424	0.795	47	4.2	21	76
9290	3.25	fair	0.382	0.397	75	4.2	20	95
9313	2.75	thin	0.108	0.62	34	3.1	21	33
9313	2.5	thin	0.477	0.573	64	3.7	22	67
9313	2.5	thin	0.711	0.76	52	3.6	20	97
9313	2.5	thin	0.474	0.51	52	3.3	20	77
9313	2.25	thin	0.2	0.802	48	3.6	23	79
9313	2.25	thin	0.284	0.628	50	3.8	20	72
9341	4	fat	0.135	0.565	37	3.6	25	55
9341	4	fat	0.429	0.377	52	4	21	75
9341	4	fat	0.42	0.515	54	3.7	18	64
9341	4	fat	0.411	0.343	49	3.8	16	65
9341	4.25	fat	0.245	0.36	56	3.9	21	76
9341	4.25	fat	0.151	.	54	3.8	20	74
9352	2.75	thin	0.099	0.41	42	3.3	18	41
9352	2.25	thin	0.413	0.522	52	3.8	28	67
9352	2.25	thin	0.199	0.454	43	3.8	22	63
9352	2.25	thin	0.131	0.395	66	3.7	18	64
9352	2.75	thin	0.155	0.418	72	3.9	23	67
9352	2.75	thin	0.112	0.415	71	3.7	26	63

Cow	BCS	BCSg	NEFA	BHBA	GLU	Alb	Urea	AST
9358	3.25	fair	0.095	0.474	67	3.6	20	47
9358	2.25	thin	0.657	0.683	54	4	21	79
9358	2.25	thin	0.345	0.552	71	4.1	20	80
9358	2.25	thin	0.174	0.5	73	3.9	21	72
9358	2.5	thin	0.324	0.874	65	3.9	25	74
9358	2.5	thin	0.291	0.708	72	3.8	13	73
9359	3.25	fair	0.031	0.302	52	4.1	31	64
9359	3	fair	0.369	0.309	57	4.1	23	83
9359	3	fair	0.059	0.316	35	3.2	18	61
9359	3	fair	0.322	0.445	31	3.7	21	63
9359	3.75	fat	0.237	0.367	51	4.1	21	74
9359	3.75	fat	0.11	.	51	3.9	21	73
9365	2.75	thin	0.102	0.476	65	3.9	24	45
9365	2.5	thin	0.322	0.646	46	4	26	52
9365	2.5	thin	0.192	0.442	51	4.1	30	49
9365	2.5	thin	0.317	0.447	46	3.7	22	49
9365	2.75	thin	0.509	0.593	41	4.2	22	57
9365	2.75	thin	0.195	0.479	51	4.1	16	61
9375	4.5	fat	0.2	0.407	58	4	20	29
9375	3.75	fat	0.777	0.932	49	2.7	10	46
9375	3.75	fat	0.987	0.907	75	3.8	14	63
9375	3.75	fat	0.739	0.777	52	3.2	13	63
9375	3.5	fair	0.767	0.728	57	3.3	14	59
9375	3.5	fair	0.473	.	65	3.8	14	63
9377	3.5	fair	0.173	0.582	75	3.7	25	64
9377	3.5	fair	0.904	0.537	89	3.6	21	83
9377	3.5	fair	0.47	0.598	80	3.2	19	71
9377	3.5	fair	0.581	0.494	93	3.5	20	81
9377	3.2	fair	1.09	0.767	82	3.4	19	79
9377	3.25	fair	0.536	0.61	81	3.4	25	78
9435	3.75	fat	0.159	0.398	55	4	26	67
9435	3.75	fat	1.88	1.46	36	4	24	88
9435	3.75	fat	1.48	2.17	53	3.9	26	115
9435	3.75	fat	1.07	1.65	60	3.7	23	131
9435	2.75	thin	0.796	1.17	59	3.8	24	156
9435	2.75	thin	0.542	0.473	62	3.8	18	98
9587	3.75	fat	0.113	0.36	68	4	21	51
9587	2.75	thin	0.335	0.537	68	4	21	58
9587	2.75	thin	0.358	0.655	63	4.1	18	48
9587	2.75	thin	0.427	0.621	62	4.2	25	45
9587	2.75	thin	0.255	0.43	65	4.1	23	54
9587	2.75	thin	0.244	0.628	69	4.1	22	50
9594	3.75	fat	0.102	0.298	53	3.1	19	46
9594	3	fair	1.18	3.16	56	2.7	12	469
9594	3	fair	0.515	2.39	62	2.8	13	467
9594	3	fair	0.635	1.46	27	2.1	10	403
9594	2.25	thin	0.756	1.17	55	2.9	13	562
9594	2.25	thin	0.368	.	46	2.8	15	435

Cow	BCS	BCSg	NEFA	BHBA	GLU	Alb	Urea	AST
9929	3.5	fair	0.241	0.626	61	3.4	19	97
9929	3.5	fair	0.238	0.482	78	3.2	19	94
9929	3.5	fair	0.283	0.565	82	3.2	20	98
9929	3.5	fair	0.331	0.7	76	3.5	22	101
9929	2.75	thin	0.624	0.699	72	3.4	22	97
9929	2.75	thin	0.511	0.694	81	3.7	20	84
9948	3.25	fair	0.121	0.662	48	3.7	31	82
9948	2.5	thin	0.301	0.597	56	3.9	31	65
9948	2.5	thin	0.622	0.492	57	4.5	37	70
9948	2.5	thin	0.4	0.588	56	4.1	28	64
9948	2.5	thin	0.562	0.461	44	4	28	78
9948	2.5	thin	0.133	0.628	59	4.1	31	66
51008	3	fair	0.031	0.459	64	3.7	26	62
51008	2.25	thin	0.225	0.437	40	4.1	28	61
51008	2.25	thin	0.192	0.372	28	3.4	20	59
51008	2.25	thin	0.227	0.462	22	3.8	22	55
51008	2.5	thin	0.291	0.544	27	4.1	20	64
51008	2.5	thin	0.175	.	42	3.9	25	60
51108	3.75	fat	0.11	0.719	56	3.8	23	99
51108	2.5	thin	0.525	0.853	46	4.2	24	142
51108	2.5	thin	0.444	0.805	42	3.9	19	102
51108	2.5	thin	0.392	0.678	37	3.9	17	115
51108	2.25	thin	0.205	0.6	41	3.8	20	72
51108	2.25	thin	0.177	0.611	56	4.5	19	99
55112	3.5	fair	0.041	0.517	58	3.4	23	74
55112	4	fat	0.215	0.535	83	3.2	18	91
55112	4	fat	0.385	0.517	64	4	26	97
55112	4	fat	0.345	0.55	67	3.6	22	72
55112	3.75	fat	0.969	0.622	68	3.5	21	68
55112	3.75	fat	0.672	1.19	61	3.4	25	166
81580	3.75	fat	0.175	0.614	58	3.9	20	87
81580	2.5	thin	0.328	0.768	49	3.6	16	74
81580	2.5	thin	0.462	0.705	59	3.6	13	88
81580	2.5	thin	0.589	0.775	52	3.2	15	89
81580	2.5	thin	0.71	1.25	43	3.5	15	82
81580	2.5	thin	0.356	0.607	49	3.7	13	94
82520	3.5	fair	0.105	0.333	62	4.2	21	60
82520	4.5	fat	0.568	1	45	3.7	16	79
82520	4.5	fat	1.69	1.29	50	3.7	20	78
82520	4.5	fat	1.35	1.79	49	3.9	22	79
82520	3	fair	0.726	2.63	44	4.2	22	89
82520	3	fair	0.564	2.54	57	4.2	20	87
91410	3.25	fair	0.138	0.479	52	3.7	20	84
91410	3	fair	0.578	0.59	55	4.1	24	85
91410	3	fair	0.331	0.555	59	4.2	25	70
91410	3	fair	0.177	0.444	41	3.8	17	63
91410	2.75	thin	0.375	0.621	49	3.9	27	67
91410	2.75	thin	0.144	0.44	53	4.1	20	74

Cow	BCS	BCSg	NEFA	BHBA	GLU	Alb	Urea	AST
91780	3.5	fair	0.095	0.28	62	4.2	32	56
91780	3.25	fair	0.755	0.459	54	3.3	20	68
91780	3.25	fair	1.07	1.2	46	3.8	26	206
91780	3.25	fair	0.505	0.832	61	2.9	19	190
91780	2.5	thin	0.522	0.599	103	3.6	26	162
91780	2.5	thin	0.295	0.314	65	3.3	17	60

Cow	Dystocia	MilkFever	RFM	Metritis
28	1	0	0	0
28	1	0	0	0
28	1	0	0	0
28	1	0	0	0
28	1	0	0	0
28	1	0	0	0
47	1	0	1	0
47	1	0	1	0
47	1	0	1	0
47	1	0	1	0
47	1	0	1	0
47	1	0	1	0
56	0	0	0	0
56	0	0	0	0
56	0	0	0	0
56	0	0	0	0
56	0	0	0	0
56	0	0	0	0
67	1	0	0	0
67	1	0	0	0
67	1	0	0	0
67	1	0	0	0
67	1	0	0	0
67	1	0	0	0
71	1	0	0	0
71	1	0	0	0
71	1	0	0	0
71	1	0	0	0
71	1	0	0	0
71	1	0	0	0
78	1	0	0	0
78	1	0	0	0
78	1	0	0	0
78	1	0	0	0
78	1	0	0	0
78	1	0	0	0
94	0	0	0	0
94	0	0	0	0
94	0	0	0	0
94	0	0	0	0
94	0	0	0	0
94	0	0	0	0
95	1	0	0	0
95	1	0	0	0
95	1	0	0	0
95	1	0	0	0
95	1	0	0	0
95	1	0	0	0

Cow	Dystocia	MilkFever	RFM	Metritis
99	0	0	0	0
99	0	0	0	0
99	0	0	0	0
99	0	0	0	0
99	0	0	0	0
99	0	0	0	0
102	0	0	0	0
102	0	0	0	0
102	0	0	0	0
102	0	0	0	0
102	0	0	0	0
102	0	0	0	0
106	0	0	0	0
106	0	0	0	0
106	0	0	0	0
106	0	0	0	0
106	0	0	0	0
106	0	0	0	0
107	0	0	0	0
107	0	0	0	0
107	0	0	0	0
107	0	0	0	0
107	0	0	0	0
107	0	0	0	0
110	1	0	0	0
110	1	0	0	0
110	1	0	0	0
110	1	0	0	0
110	1	0	0	0
110	1	0	0	0
112	0	0	1	1
112	0	0	1	1
112	0	0	1	1
112	0	0	1	1
112	0	0	1	1
112	0	0	1	1
118	1	0	0	0
118	1	0	0	0
118	1	0	0	0
118	1	0	0	0
118	1	0	0	0
118	1	0	0	0
120	0	0	0	1
120	0	0	0	1
120	0	0	0	1
120	0	0	0	1
120	0	0	0	1
120	0	0	0	1

Cow	Dystocia	MilkFever	RFM	Metritis
132	0	0	0	0
132	0	0	0	0
132	0	0	0	0
132	0	0	0	0
132	0	0	0	0
132	0	0	0	0
147	0	0	1	1
147	0	0	1	1
147	0	0	1	1
147	0	0	1	1
147	0	0	1	1
147	0	0	1	1
151	1	0	0	0
151	1	0	0	0
151	1	0	0	0
151	1	0	0	0
151	1	0	0	0
151	1	0	0	0
186	0	0	1	1
186	0	0	1	1
186	0	0	1	1
186	0	0	1	1
186	0	0	1	1
186	0	0	1	1
188	1	0	0	0
188	1	0	0	0
188	1	0	0	0
188	1	0	0	0
188	1	0	0	0
188	1	0	0	0
190	0	0	0	0
190	0	0	0	0
190	0	0	0	0
190	0	0	0	0
190	0	0	0	0
190	0	0	0	0
208	1	0	0	0
208	1	0	0	0
208	1	0	0	0
208	1	0	0	0
208	1	0	0	0
208	1	0	0	0
224	1	0	0	0
224	1	0	0	0
224	1	0	0	0
224	1	0	0	0
224	1	0	0	0
224	1	0	0	0

Cow	Dystocia	MilkFever	RFM	Metritis
225	0	0	0	0
225	0	0	0	0
225	0	0	0	0
225	0	0	0	0
225	0	0	0	0
225	0	0	0	0
231	0	0	0	0
231	0	0	0	0
231	0	0	0	0
231	0	0	0	0
231	0	0	0	0
231	0	0	0	0
245	0	0	1	1
245	0	0	1	1
245	0	0	1	1
245	0	0	1	1
245	0	0	1	1
245	0	0	1	1
247	1	0	0	0
247	1	0	0	0
247	1	0	0	0
247	1	0	0	0
247	1	0	0	0
247	1	0	0	0
248	1	0	0	0
248	1	0	0	0
248	1	0	0	0
248	1	0	0	0
248	1	0	0	0
248	1	0	0	0
249	0	0	0	1
249	0	0	0	1
249	0	0	0	1
249	0	0	0	1
249	0	0	0	1
249	0	0	0	1
256	1	0	0	0
256	1	0	0	0
256	1	0	0	0
256	1	0	0	0
256	1	0	0	0
256	1	0	0	0
261	0	0	0	0
261	0	0	0	0
261	0	0	0	0
261	0	0	0	0
261	0	0	0	0
261	0	0	0	0

Cow	Dystocia	MilkFever	RFM	Metritis
329	0	0	0	0
329	0	0	0	0
329	0	0	0	0
329	0	0	0	0
329	0	0	0	0
329	0	0	0	0
337	0	0	0	0
337	0	0	0	0
337	0	0	0	0
337	0	0	0	0
337	0	0	0	0
337	0	0	0	0
524	0	0	0	1
524	0	0	0	1
524	0	0	0	1
524	0	0	0	1
524	0	0	0	1
524	0	0	0	1
662	0	0	0	0
662	0	0	0	0
662	0	0	0	0
662	0	0	0	0
662	0	0	0	0
662	0	0	0	0
731	0	0	0	0
731	0	0	0	0
731	0	0	0	0
731	0	0	0	0
731	0	0	0	0
731	0	0	0	0
813	0	0	1	1
813	0	0	1	1
813	0	0	1	1
813	0	0	1	1
813	0	0	1	1
813	0	0	1	1
856	0	0	0	0
856	0	0	0	0
856	0	0	0	0
856	0	0	0	0
856	0	0	0	0
856	0	0	0	0
938	0	0	0	0
938	0	0	0	0
938	0	0	0	0
938	0	0	0	0
938	0	0	0	0
938	0	0	0	0

Cow	Dystocia	MilkFever	RFM	Metritis
944	0	0	0	0
944	0	0	0	0
944	0	0	0	0
944	0	0	0	0
944	0	0	0	0
944	0	0	0	0
1281	0	0	0	0
1281	0	0	0	0
1281	0	0	0	0
1281	0	0	0	0
1281	0	0	0	0
1281	0	0	0	0
2493	1	0	0	0
2493	1	0	0	0
2493	1	0	0	0
2493	1	0	0	0
2493	1	0	0	0
2493	1	0	0	0
2624	0	0	1	1
2624	0	0	1	1
2624	0	0	1	1
2624	0	0	1	1
2624	0	0	1	1
2624	0	0	1	1
2694	0	0	0	0
2694	0	0	0	0
2694	0	0	0	0
2694	0	0	0	0
2694	0	0	0	0
2694	0	0	0	0
2741	0	0	0	0
2741	0	0	0	0
2741	0	0	0	0
2741	0	0	0	0
2741	0	0	0	0
2741	0	0	0	0
2764	0	0	0	0
2764	0	0	0	0
2764	0	0	0	0
2764	0	0	0	0
2764	0	0	0	0
2764	0	0	0	0
2813	0	0	1	0
2813	0	0	1	0
2813	0	0	1	0
2813	0	0	1	0
2813	0	0	1	0
2813	0	0	1	0

Cow	Dystocia	MilkFever	RFM	Metritis
2940	0	0	0	0
2940	0	0	0	0
2940	0	0	0	0
2940	0	0	0	0
2940	0	0	0	0
2940	0	0	0	0
3526	0	0	0	0
3526	0	0	0	0
3526	0	0	0	0
3526	0	0	0	0
3526	0	0	0	0
3526	0	0	0	0
4019	0	0	0	0
4019	0	0	0	0
4019	0	0	0	0
4019	0	0	0	0
4019	0	0	0	0
4019	0	0	0	0
4044	0	0	0	0
4044	0	0	0	0
4044	0	0	0	0
4044	0	0	0	0
4044	0	0	0	0
4044	0	0	0	0
4053	1	0	0	0
4053	1	0	0	0
4053	1	0	0	0
4053	1	0	0	0
4053	1	0	0	0
4053	1	0	0	0
5061	1	0	0	0
5061	1	0	0	0
5061	1	0	0	0
5061	1	0	0	0
5061	1	0	0	0
5061	1	0	0	0
5104	1	0	0	1
5104	1	0	0	1
5104	1	0	0	1
5104	1	0	0	1
5104	1	0	0	1
5104	1	0	0	1
5165	0	0	0	0
5165	0	0	0	0
5165	0	0	0	0
5165	0	0	0	0
5165	0	0	0	0
5165	0	0	0	0

Cow	Dystocia	MilkFever	RFM	Metritis
5170	0	0	0	0
5170	0	0	0	0
5170	0	0	0	0
5170	0	0	0	0
5170	0	0	0	0
5170	0	0	0	0
5254	0	0	1	0
5254	0	0	1	0
5254	0	0	1	0
5254	0	0	1	0
5254	0	0	1	0
5254	0	0	1	0
5371	0	0	0	0
5371	0	0	0	0
5371	0	0	0	0
5371	0	0	0	0
5371	0	0	0	0
5371	0	0	0	0
5397	0	0	0	0
5397	0	0	0	0
5397	0	0	0	0
5397	0	0	0	0
5397	0	0	0	0
5397	0	0	0	0
5405	0	0	0	0
5405	0	0	0	0
5405	0	0	0	0
5405	0	0	0	0
5405	0	0	0	0
5405	0	0	0	0
5415	0	0	1	0
5415	0	0	1	0
5415	0	0	1	0
5415	0	0	1	0
5415	0	0	1	0
5415	0	0	1	0
5771	0	0	0	0
5771	0	0	0	0
5771	0	0	0	0
5771	0	0	0	0
5771	0	0	0	0
5771	0	0	0	0
5919	0	0	1	0
5919	0	0	1	0
5919	0	0	1	0
5919	0	0	1	0
5919	0	0	1	0
5919	0	0	1	0

Cow	Dystocia	MilkFever	RFM	Metritis
5971	0	0	0	0
5971	0	0	0	0
5971	0	0	0	0
5971	0	0	0	0
5971	0	0	0	0
5971	0	0	0	0
6052	0	0	0	0
6052	0	0	0	0
6052	0	0	0	0
6052	0	0	0	0
6052	0	0	0	0
6052	0	0	0	0
6123	0	0	0	0
6123	0	0	0	0
6123	0	0	0	0
6123	0	0	0	0
6123	0	0	0	0
6123	0	0	0	0
6145	0	0	1	0
6145	0	0	1	0
6145	0	0	1	0
6145	0	0	1	0
6145	0	0	1	0
6145	0	0	1	0
6203	0	0	0	0
6203	0	0	0	0
6203	0	0	0	0
6203	0	0	0	0
6203	0	0	0	0
6203	0	0	0	0
6220	0	0	0	0
6220	0	0	0	0
6220	0	0	0	0
6220	0	0	0	0
6220	0	0	0	0
6220	0	0	0	0
6284	0	0	0	1
6284	0	0	0	1
6284	0	0	0	1
6284	0	0	0	1
6284	0	0	0	1
6284	0	0	0	1
6515	0	0	0	0
6515	0	0	0	0
6515	0	0	0	0
6515	0	0	0	0
6515	0	0	0	0
6515	0	0	0	0

Cow	Dystocia	MilkFever	RFM	Metritis
6540	0	0	0	0
6540	0	0	0	0
6540	0	0	0	0
6540	0	0	0	0
6540	0	0	0	0
6540	0	0	0	0
6724	0	0	0	0
6724	0	0	0	0
6724	0	0	0	0
6724	0	0	0	0
6724	0	0	0	0
6724	0	0	0	0
7048	1	0	1	1
7048	1	0	1	1
7048	1	0	1	1
7048	1	0	1	1
7048	1	0	1	1
7048	1	0	1	1
7050	0	0	0	1
7050	0	0	0	1
7050	0	0	0	1
7050	0	0	0	1
7050	0	0	0	1
7050	0	0	0	1
7067	0	0	0	0
7067	0	0	0	0
7067	0	0	0	0
7067	0	0	0	0
7067	0	0	0	0
7067	0	0	0	0
7068	1	0	1	0
7068	1	0	1	0
7068	1	0	1	0
7068	1	0	1	0
7068	1	0	1	0
7068	1	0	1	0
7113	1	0	0	0
7113	1	0	0	0
7113	1	0	0	0
7113	1	0	0	0
7113	1	0	0	0
7113	1	0	0	0
7121	0	0	0	0
7121	0	0	0	0
7121	0	0	0	0
7121	0	0	0	0
7121	0	0	0	0
7121	0	0	0	0

Cow	Dystocia	MilkFever	RFM	Metritis
7133	0	0	0	0
7133	0	0	0	0
7133	0	0	0	0
7133	0	0	0	0
7133	0	0	0	0
7133	0	0	0	0
7136	0	0	0	0
7136	0	0	0	0
7136	0	0	0	0
7136	0	0	0	0
7136	0	0	0	0
7136	0	0	0	0
7140	0	0	1	1
7140	0	0	1	1
7140	0	0	1	1
7140	0	0	1	1
7140	0	0	1	1
7140	0	0	1	1
7141	0	0	0	0
7141	0	0	0	0
7141	0	0	0	0
7141	0	0	0	0
7141	0	0	0	0
7141	0	0	0	0
7151	0	0	0	0
7151	0	0	0	0
7151	0	0	0	0
7151	0	0	0	0
7151	0	0	0	0
7151	0	0	0	0
7157	0	0	0	0
7157	0	0	0	0
7157	0	0	0	0
7157	0	0	0	0
7157	0	0	0	0
7157	0	0	0	0
7177	0	0	0	0
7177	0	0	0	0
7177	0	0	0	0
7177	0	0	0	0
7177	0	0	0	0
7177	0	0	0	0
7194	1	0	0	0
7194	1	0	0	0
7194	1	0	0	0
7194	1	0	0	0
7194	1	0	0	0
7194	1	0	0	0

Cow	Dystocia	MilkFever	RFM	Metritis
7268	1	0	0	0
7268	1	0	0	0
7268	1	0	0	0
7268	1	0	0	0
7268	1	0	0	0
7268	1	0	0	0
7322	1	0	0	0
7322	1	0	0	0
7322	1	0	0	0
7322	1	0	0	0
7322	1	0	0	0
7322	1	0	0	0
7353	0	0	0	0
7353	0	0	0	0
7353	0	0	0	0
7353	0	0	0	0
7353	0	0	0	0
7353	0	0	0	0
7368	1	0	0	0
7368	1	0	0	0
7368	1	0	0	0
7368	1	0	0	0
7368	1	0	0	0
7368	1	0	0	0
7403	0	0	0	0
7403	0	0	0	0
7403	0	0	0	0
7403	0	0	0	0
7403	0	0	0	0
7403	0	0	0	0
7412	0	0	0	0
7412	0	0	0	0
7412	0	0	0	0
7412	0	0	0	0
7412	0	0	0	0
7412	0	0	0	0
7521	1	0	0	0
7521	1	0	0	0
7521	1	0	0	0
7521	1	0	0	0
7521	1	0	0	0
7521	1	0	0	0
7537	1	0	0	0
7537	1	0	0	0
7537	1	0	0	0
7537	1	0	0	0
7537	1	0	0	0
7537	1	0	0	0

Cow	Dystocia	MilkFever	RFM	Metritis
7543	1	0	1	1
7543	1	0	1	1
7543	1	0	1	1
7543	1	0	1	1
7543	1	0	1	1
7543	1	0	1	1
7550	0	0	0	0
7550	0	0	0	0
7550	0	0	0	0
7550	0	0	0	0
7550	0	0	0	0
7550	0	0	0	0
7712	0	0	0	0
7712	0	0	0	0
7712	0	0	0	0
7712	0	0	0	0
7712	0	0	0	0
7712	0	0	0	0
8015	0	0	0	0
8015	0	0	0	0
8015	0	0	0	0
8015	0	0	0	0
8015	0	0	0	0
8015	0	0	0	0
8017	0	0	0	0
8017	0	0	0	0
8017	0	0	0	0
8017	0	0	0	0
8017	0	0	0	0
8017	0	0	0	0
8027	0	0	0	0
8027	0	0	0	0
8027	0	0	0	0
8027	0	0	0	0
8027	0	0	0	0
8027	0	0	0	0
8040	0	0	0	0
8040	0	0	0	0
8040	0	0	0	0
8040	0	0	0	0
8040	0	0	0	0
8040	0	0	0	0
8046	1	0	1	1
8046	1	0	1	1
8046	1	0	1	1
8046	1	0	1	1
8046	1	0	1	1
8046	1	0	1	1

Cow	Dystocia	MilkFever	RFM	Metritis
8047	1	0	0	0
8047	1	0	0	0
8047	1	0	0	0
8047	1	0	0	0
8047	1	0	0	0
8047	1	0	0	0
8082	0	0	0	0
8082	0	0	0	0
8082	0	0	0	0
8082	0	0	0	0
8082	0	0	0	0
8082	0	0	0	0
8086	0	0	0	0
8086	0	0	0	0
8086	0	0	0	0
8086	0	0	0	0
8086	0	0	0	0
8086	0	0	0	0
8122	1	0	0	0
8122	1	0	0	0
8122	1	0	0	0
8122	1	0	0	0
8122	1	0	0	0
8122	1	0	0	0
8129	0	0	0	0
8129	0	0	0	0
8129	0	0	0	0
8129	0	0	0	0
8129	0	0	0	0
8129	0	0	0	0
8133	0	0	0	0
8133	0	0	0	0
8133	0	0	0	0
8133	0	0	0	0
8133	0	0	0	0
8133	0	0	0	0
8152	0	0	0	0
8152	0	0	0	0
8152	0	0	0	0
8152	0	0	0	0
8152	0	0	0	0
8152	0	0	0	0
8154	0	0	0	0
8154	0	0	0	0
8154	0	0	0	0
8154	0	0	0	0
8154	0	0	0	0
8154	0	0	0	0

Cow	Dystocia	MilkFever	RFM	Metritis
8156	0	0	1	1
8156	0	0	1	1
8156	0	0	1	1
8156	0	0	1	1
8156	0	0	1	1
8156	0	0	1	1
8158	0	0	0	0
8158	0	0	0	0
8158	0	0	0	0
8158	0	0	0	0
8158	0	0	0	0
8158	0	0	0	0
8160	0	0	0	0
8160	0	0	0	0
8160	0	0	0	0
8160	0	0	0	0
8160	0	0	0	0
8160	0	0	0	0
8177	0	0	0	0
8177	0	0	0	0
8177	0	0	0	0
8177	0	0	0	0
8177	0	0	0	0
8177	0	0	0	0
8181	0	0	0	0
8181	0	0	0	0
8181	0	0	0	0
8181	0	0	0	0
8181	0	0	0	0
8181	0	0	0	0
8186	0	0	0	0
8186	0	0	0	0
8186	0	0	0	0
8186	0	0	0	0
8186	0	0	0	0
8186	0	0	0	0
8221	1	0	1	0
8221	1	0	1	0
8221	1	0	1	0
8221	1	0	1	0
8221	1	0	1	0
8221	1	0	1	0
8230	0	0	0	0
8230	0	0	0	0
8230	0	0	0	0
8230	0	0	0	0
8230	0	0	0	0
8230	0	0	0	0

Cow	Dystocia	MilkFever	RFM	Metritis
8244	1	0	0	0
8244	1	0	0	0
8244	1	0	0	0
8244	1	0	0	0
8244	1	0	0	0
8244	1	0	0	0
8252	0	0	0	0
8252	0	0	0	0
8252	0	0	0	0
8252	0	0	0	0
8252	0	0	0	0
8252	0	0	0	0
8268	0	0	0	0
8268	0	0	0	0
8268	0	0	0	0
8268	0	0	0	0
8268	0	0	0	0
8268	0	0	0	0
8313	0	0	1	0
8313	0	0	1	0
8313	0	0	1	0
8313	0	0	1	0
8313	0	0	1	0
8313	0	0	1	0
8339	1	0	0	0
8339	1	0	0	0
8339	1	0	0	0
8339	1	0	0	0
8339	1	0	0	0
8339	1	0	0	0
8372	1	0	0	0
8372	1	0	0	0
8372	1	0	0	0
8372	1	0	0	0
8372	1	0	0	0
8372	1	0	0	0
8375	0	0	1	1
8375	0	0	1	1
8375	0	0	1	1
8375	0	0	1	1
8375	0	0	1	1
8375	0	0	1	1
8376	0	0	0	0
8376	0	0	0	0
8376	0	0	0	0
8376	0	0	0	0
8376	0	0	0	0
8376	0	0	0	0

Cow	Dystocia	MilkFever	RFM	Metritis
8389	0	0	0	0
8389	0	0	0	0
8389	0	0	0	0
8389	0	0	0	0
8389	0	0	0	0
8389	0	0	0	0
8393	0	0	0	0
8393	0	0	0	0
8393	0	0	0	0
8393	0	0	0	0
8393	0	0	0	0
8393	0	0	0	0
8398	0	0	0	0
8398	0	0	0	0
8398	0	0	0	0
8398	0	0	0	0
8398	0	0	0	0
8398	0	0	0	0
8401	0	0	0	0
8401	0	0	0	0
8401	0	0	0	0
8401	0	0	0	0
8401	0	0	0	0
8401	0	0	0	0
8402	0	0	1	0
8402	0	0	1	0
8402	0	0	1	0
8402	0	0	1	0
8402	0	0	1	0
8402	0	0	1	0
8414	0	0	1	1
8414	0	0	1	1
8414	0	0	1	1
8414	0	0	1	1
8414	0	0	1	1
8414	0	0	1	1
8469	0	0	0	0
8469	0	0	0	0
8469	0	0	0	0
8469	0	0	0	0
8469	0	0	0	0
8469	0	0	0	0
8510	1	0	0	0
8510	1	0	0	0
8510	1	0	0	0
8510	1	0	0	0
8510	1	0	0	0
8510	1	0	0	0

Cow	Dystocia	MilkFever	RFM	Metritis
8522	1	0	0	0
8522	1	0	0	0
8522	1	0	0	0
8522	1	0	0	0
8522	1	0	0	0
8522	1	0	0	0
8599	0	0	1	1
8599	0	0	1	1
8599	0	0	1	1
8599	0	0	1	1
8599	0	0	1	1
8599	0	0	1	1
8684	1	0	0	0
8684	1	0	0	0
8684	1	0	0	0
8684	1	0	0	0
8684	1	0	0	0
8684	1	0	0	0
8704	1	0	0	1
8704	1	0	0	1
8704	1	0	0	1
8704	1	0	0	1
8704	1	0	0	1
8704	1	0	0	1
8706	0	0	0	0
8706	0	0	0	0
8706	0	0	0	0
8706	0	0	0	0
8706	0	0	0	0
8706	0	0	0	0
9004	1	0	0	0
9004	1	0	0	0
9004	1	0	0	0
9004	1	0	0	0
9004	1	0	0	0
9004	1	0	0	0
9027	1	0	0	0
9027	1	0	0	0
9027	1	0	0	0
9027	1	0	0	0
9027	1	0	0	0
9027	1	0	0	0
9028	0	0	0	0
9028	0	0	0	0
9028	0	0	0	0
9028	0	0	0	0
9028	0	0	0	0
9028	0	0	0	0

Cow	Dystocia	MilkFever	RFM	Metritis
9030	0	0	0	0
9030	0	0	0	0
9030	0	0	0	0
9030	0	0	0	0
9030	0	0	0	0
9030	0	0	0	0
9031	0	0	1	0
9031	0	0	1	0
9031	0	0	1	0
9031	0	0	1	0
9031	0	0	1	0
9031	0	0	1	0
9060	0	0	0	0
9060	0	0	0	0
9060	0	0	0	0
9060	0	0	0	0
9060	0	0	0	0
9060	0	0	0	0
9066	0	0	0	0
9066	0	0	0	0
9066	0	0	0	0
9066	0	0	0	0
9066	0	0	0	0
9066	0	0	0	0
9074	0	0	0	0
9074	0	0	0	0
9074	0	0	0	0
9074	0	0	0	0
9074	0	0	0	0
9074	0	0	0	0
9087	0	0	0	0
9087	0	0	0	0
9087	0	0	0	0
9087	0	0	0	0
9087	0	0	0	0
9087	0	0	0	0
9092	0	0	0	0
9092	0	0	0	0
9092	0	0	0	0
9092	0	0	0	0
9092	0	0	0	0
9092	0	0	0	0
9101	0	0	0	0
9101	0	0	0	0
9101	0	0	0	0
9101	0	0	0	0
9101	0	0	0	0
9101	0	0	0	0

Cow	Dystocia	MilkFever	RFM	Metritis
9112	0	0	0	0
9112	0	0	0	0
9112	0	0	0	0
9112	0	0	0	0
9112	0	0	0	0
9112	0	0	0	0
9123	1	0	0	0
9123	1	0	0	0
9123	1	0	0	0
9123	1	0	0	0
9123	1	0	0	0
9123	1	0	0	0
9128	0	0	0	0
9128	0	0	0	0
9128	0	0	0	0
9128	0	0	0	0
9128	0	0	0	0
9128	0	0	0	0
9132	1	0	0	0
9132	1	0	0	0
9132	1	0	0	0
9132	1	0	0	0
9132	1	0	0	0
9132	1	0	0	0
9141	0	0	0	0
9141	0	0	0	0
9141	0	0	0	0
9141	0	0	0	0
9141	0	0	0	0
9141	0	0	0	0
9147	0	0	0	0
9147	0	0	0	0
9147	0	0	0	0
9147	0	0	0	0
9147	0	0	0	0
9147	0	0	0	0
9160	0	0	0	0
9160	0	0	0	0
9160	0	0	0	0
9160	0	0	0	0
9160	0	0	0	0
9160	0	0	0	0
9166	0	0	0	0
9166	0	0	0	0
9166	0	0	0	0
9166	0	0	0	0
9166	0	0	0	0
9166	0	0	0	0

Cow	Dystocia	MilkFever	RFM	Metritis
9167	0	0	0	0
9167	0	0	0	0
9167	0	0	0	0
9167	0	0	0	0
9167	0	0	0	0
9167	0	0	0	0
9168	0	0	0	0
9168	0	0	0	0
9168	0	0	0	0
9168	0	0	0	0
9168	0	0	0	0
9168	0	0	0	0
9178	1	0	0	1
9178	1	0	0	1
9178	1	0	0	1
9178	1	0	0	1
9178	1	0	0	1
9178	1	0	0	1
9181	1	0	0	0
9181	1	0	0	0
9181	1	0	0	0
9181	1	0	0	0
9181	1	0	0	0
9181	1	0	0	0
9194	0	0	0	0
9194	0	0	0	0
9194	0	0	0	0
9194	0	0	0	0
9194	0	0	0	0
9194	0	0	0	0
9204	0	0	0	0
9204	0	0	0	0
9204	0	0	0	0
9204	0	0	0	0
9204	0	0	0	0
9204	0	0	0	0
9210	0	0	0	0
9210	0	0	0	0
9210	0	0	0	0
9210	0	0	0	0
9210	0	0	0	0
9210	0	0	0	0
9211	1	0	0	1
9211	1	0	0	1
9211	1	0	0	1
9211	1	0	0	1
9211	1	0	0	1
9211	1	0	0	1

Cow	Dystocia	MilkFever	RFM	Metritis
9218	0	0	0	0
9218	0	0	0	0
9218	0	0	0	0
9218	0	0	0	0
9218	0	0	0	0
9218	0	0	0	0
9226	1	0	0	0
9226	1	0	0	0
9226	1	0	0	0
9226	1	0	0	0
9226	1	0	0	0
9226	1	0	0	0
9228	0	0	0	0
9228	0	0	0	0
9228	0	0	0	0
9228	0	0	0	0
9228	0	0	0	0
9228	0	0	0	0
9229	0	0	0	0
9229	0	0	0	0
9229	0	0	0	0
9229	0	0	0	0
9229	0	0	0	0
9229	0	0	0	0
9290	0	0	0	0
9290	0	0	0	0
9290	0	0	0	0
9290	0	0	0	0
9290	0	0	0	0
9290	0	0	0	0
9313	0	0	0	0
9313	0	0	0	0
9313	0	0	0	0
9313	0	0	0	0
9313	0	0	0	0
9313	0	0	0	0
9341	0	0	0	0
9341	0	0	0	0
9341	0	0	0	0
9341	0	0	0	0
9341	0	0	0	0
9341	0	0	0	0
9352	0	0	0	0
9352	0	0	0	0
9352	0	0	0	0
9352	0	0	0	0
9352	0	0	0	0
9352	0	0	0	0

Cow	Dystocia	MilkFever	RFM	Metritis
9358	0	0	0	0
9358	0	0	0	0
9358	0	0	0	0
9358	0	0	0	0
9358	0	0	0	0
9358	0	0	0	0
9359	0	0	0	0
9359	0	0	0	0
9359	0	0	0	0
9359	0	0	0	0
9359	0	0	0	0
9359	0	0	0	0
9365	0	0	0	0
9365	0	0	0	0
9365	0	0	0	0
9365	0	0	0	0
9365	0	0	0	0
9365	0	0	0	0
9375	1	0	0	0
9375	1	0	0	0
9375	1	0	0	0
9375	1	0	0	0
9375	1	0	0	0
9375	1	0	0	0
9377	0	0	0	0
9377	0	0	0	0
9377	0	0	0	0
9377	0	0	0	0
9377	0	0	0	0
9377	0	0	0	0
9435	1	0	0	0
9435	1	0	0	0
9435	1	0	0	0
9435	1	0	0	0
9435	1	0	0	0
9435	1	0	0	0
9587	0	0	0	0
9587	0	0	0	0
9587	0	0	0	0
9587	0	0	0	0
9587	0	0	0	0
9587	0	0	0	0
9594	1	0	0	0
9594	1	0	0	0
9594	1	0	0	0
9594	1	0	0	0
9594	1	0	0	0
9594	1	0	0	0

Cow	Dystocia	MilkFever	RFM	Metritis
9929	0	0	0	0
9929	0	0	0	0
9929	0	0	0	0
9929	0	0	0	0
9929	0	0	0	0
9929	0	0	0	0
9948	1	0	0	0
9948	1	0	0	0
9948	1	0	0	0
9948	1	0	0	0
9948	1	0	0	0
9948	1	0	0	0
51008	0	0	0	0
51008	0	0	0	0
51008	0	0	0	0
51008	0	0	0	0
51008	0	0	0	0
51008	0	0	0	0
51108	0	0	0	0
51108	0	0	0	0
51108	0	0	0	0
51108	0	0	0	0
51108	0	0	0	0
51108	0	0	0	0
55112	0	0	0	0
55112	0	0	0	0
55112	0	0	0	0
55112	0	0	0	0
55112	0	0	0	0
55112	0	0	0	0
81580	0	0	1	1
81580	0	0	1	1
81580	0	0	1	1
81580	0	0	1	1
81580	0	0	1	1
81580	0	0	1	1
82520	0	0	0	0
82520	0	0	0	0
82520	0	0	0	0
82520	0	0	0	0
82520	0	0	0	0
82520	0	0	0	0
91410	0	0	0	0
91410	0	0	0	0
91410	0	0	0	0
91410	0	0	0	0
91410	0	0	0	0
91410	0	0	0	0

Cow	Dystocia	MilkFever	RFM	Metritis
91780	0	0	1	0
91780	0	0	1	0
91780	0	0	1	0
91780	0	0	1	0
91780	0	0	1	0
91780	0	0	1	0

Cow	Cholesterol
28	65
28	69
28	70
28	97
28	101
28	104
47	71
47	80
47	80
47	83
47	83
47	84
56	76
56	79
56	80
56	85
56	106
56	.
67	99
67	100
67	107
67	137
67	137
67	152
71	83
71	83
71	89
71	120
71	132
71	.
78	107
78	108
78	109
78	123
78	128
78	.
94	88
94	97
94	100
94	117
94	117
94	131
95	70
95	71
95	72
95	87
95	106
95	.

Cow	Cholesterol
99	88
99	92
99	95
99	96
99	108
99	122
102	122
102	131
102	134
102	136
102	141
102	141
106	123
106	130
106	135
106	141
106	145
106	173
107	133
107	138
107	165
107	184
107	187
107	.
110	116
110	124
110	130
110	134
110	163
110	165
112	65
112	80
112	92
112	97
112	109
112	.
118	77
118	82
118	85
118	93
118	94
118	.
120	64
120	77
120	92
120	100
120	108
120	.

Cow	Cholesterol
132	116
132	129
132	137
132	139
132	145
132	168
147	101
147	106
147	108
147	128
147	147
147	186
151	95
151	102
151	130
151	138
151	144
151	171
186	66
186	75
186	78
186	82
186	103
186	.
188	93
188	97
188	102
188	108
188	.
188	.
190	109
190	115
190	117
190	126
190	128
190	145
208	70
208	73
208	82
208	83
208	114
208	118
224	79
224	87
224	95
224	102
224	107
224	111

Cow	Cholesterol
225	110
225	110
225	115
225	120
225	122
225	145
231	107
231	112
231	117
231	133
231	137
231	160
245	69
245	72
245	83
245	90
245	111
245	131
247	122
247	126
247	129
247	138
247	153
247	171
248	54
248	60
248	70
248	72
248	.
248	.
249	50
249	95
249	98
249	102
249	137
249	148
256	120
256	125
256	138
256	139
256	145
256	174
261	108
261	111
261	130
261	139
261	143
261	152

Cow	Cholesterol
329	33
329	44
329	74
329	81
329	101
329	.
337	102
337	102
337	109
337	112
337	137
337	168
524	63
524	65
524	87
524	105
524	.
524	.
662	67
662	67
662	71
662	84
662	101
662	131
731	83
731	88
731	100
731	106
731	124
731	146
813	88
813	102
813	111
813	112
813	.
813	.
856	95
856	102
856	105
856	117
856	120
856	167
938	124
938	128
938	140
938	142
938	153
938	202

Cow	Cholesterol
944	83
944	95
944	111
944	111
944	123
944	149
1281	86
1281	89
1281	91
1281	93
1281	105
1281	111
2493	60
2493	76
2493	89
2493	91
2493	98
2493	.
2624	86
2624	87
2624	89
2624	92
2624	131
2624	195
2694	15
2694	68
2694	70
2694	71
2694	81
2694	93
2741	44
2741	67
2741	73
2741	79
2741	108
2741	120
2764	107
2764	112
2764	116
2764	129
2764	132
2764	.
2813	115
2813	116
2813	117
2813	120
2813	124
2813	131

Cow	Cholesterol
2940	65
2940	82
2940	84
2940	104
2940	.
2940	.
3526	125
3526	143
3526	165
3526	183
3526	188
3526	206
4019	122
4019	123
4019	124
4019	145
4019	149
4019	.
4044	102
4044	118
4044	127
4044	131
4044	141
4044	163
4053	78
4053	80
4053	86
4053	.
4053	.
4053	.
5061	90
5061	93
5061	102
5061	124
5061	125
5061	.
5104	65
5104	67
5104	75
5104	82
5104	105
5104	.
5165	80
5165	91
5165	104
5165	106
5165	111
5165	.

Cow	Cholesterol
5170	74
5170	80
5170	94
5170	104
5170	118
5170	144
5254	45
5254	61
5254	76
5254	79
5254	79
5254	.
5371	111
5371	125
5371	135
5371	139
5371	145
5371	182
5397	101
5397	121
5397	131
5397	140
5397	154
5397	.
5405	86
5405	112
5405	115
5405	136
5405	151
5405	.
5415	64
5415	66
5415	69
5415	69
5415	84
5415	96
5771	75
5771	75
5771	77
5771	82
5771	85
5771	86
5919	80
5919	96
5919	98
5919	102
5919	121
5919	127

Cow	Cholesterol
5971	76
5971	80
5971	84
5971	88
5971	94
5971	.
6052	124
6052	144
6052	149
6052	152
6052	160
6052	208
6123	56
6123	67
6123	79
6123	83
6123	103
6123	.
6145	81
6145	113
6145	122
6145	131
6145	134
6145	.
6203	87
6203	103
6203	103
6203	111
6203	130
6203	.
6220	71
6220	86
6220	90
6220	99
6220	.
6220	.
6284	67
6284	74
6284	79
6284	84
6284	106
6284	110
6515	54
6515	70
6515	79
6515	84
6515	91
6515	.

Cow	Cholesterol
6540	74
6540	79
6540	83
6540	88
6540	88
6540	115
6724	128
6724	136
6724	160
6724	170
6724	173
6724	185
7048	41
7048	58
7048	58
7048	71
7048	85
7048	93
7050	66
7050	77
7050	83
7050	100
7050	116
7050	.
7067	100
7067	112
7067	120
7067	136
7067	142
7067	158
7068	104
7068	107
7068	112
7068	130
7068	141
7068	157
7113	79
7113	99
7113	101
7113	112
7113	144
7113	.
7121	92
7121	103
7121	107
7121	111
7121	115
7121	138

Cow	Cholesterol
7133	65
7133	101
7133	104
7133	104
7133	107
7133	128
7136	45
7136	82
7136	88
7136	92
7136	99
7136	104
7140	43
7140	44
7140	58
7140	113
7140	.
7140	.
7141	89
7141	92
7141	97
7141	99
7141	109
7141	132
7151	67
7151	100
7151	102
7151	104
7151	112
7151	121
7157	66
7157	82
7157	86
7157	103
7157	112
7157	.
7177	90
7177	101
7177	102
7177	112
7177	131
7177	.
7194	86
7194	93
7194	104
7194	118
7194	119
7194	121

Cow	Cholesterol
7268	84
7268	86
7268	101
7268	103
7268	115
7268	.
7322	52
7322	56
7322	61
7322	75
7322	89
7322	162
7353	89
7353	94
7353	103
7353	104
7353	120
7353	147
7368	63
7368	71
7368	77
7368	82
7368	104
7368	111
7403	83
7403	85
7403	89
7403	93
7403	113
7403	125
7412	71
7412	72
7412	92
7412	101
7412	.
7412	.
7521	115
7521	127
7521	148
7521	148
7521	154
7521	195
7537	78
7537	81
7537	87
7537	88
7537	89
7537	.

Cow	Cholesterol
7543	57
7543	62
7543	88
7543	95
7543	96
7543	.
7550	84
7550	84
7550	87
7550	99
7550	113
7550	118
7712	63
7712	92
7712	111
7712	115
7712	122
7712	135
8015	75
8015	75
8015	77
8015	81
8015	91
8015	97
8017	130
8017	131
8017	133
8017	134
8017	138
8017	165
8027	67
8027	106
8027	109
8027	112
8027	135
8027	.
8040	93
8040	97
8040	107
8040	113
8040	117
8040	.
8046	64
8046	83
8046	94
8046	108
8046	115
8046	119

Cow	Cholesterol
8047	100
8047	112
8047	115
8047	117
8047	142
8047	170
8082	131
8082	136
8082	137
8082	141
8082	141
8082	163
8086	136
8086	142
8086	144
8086	146
8086	174
8086	190
8122	131
8122	135
8122	143
8122	144
8122	165
8122	.
8129	106
8129	114
8129	129
8129	138
8129	192
8129	.
8133	103
8133	111
8133	111
8133	126
8133	137
8133	213
8152	130
8152	138
8152	149
8152	152
8152	153
8152	158
8154	93
8154	99
8154	113
8154	122
8154	123
8154	.

Cow	Cholesterol
8156	53
8156	54
8156	86
8156	.
8156	.
8156	.
8158	73
8158	77
8158	79
8158	80
8158	88
8158	114
8158	153
8158	160
8158	172
8158	174
8158	180
8158	257
8160	82
8160	83
8160	99
8160	105
8160	114
8160	121
8177	80
8177	82
8177	85
8177	99
8177	112
8177	143
8181	99
8181	104
8181	111
8181	112
8181	123
8181	157
8186	74
8186	78
8186	80
8186	90
8186	92
8186	118
8221	91
8221	92
8221	106
8221	108
8221	119
8221	150

Cow	Cholesterol
8230	102
8230	123
8230	125
8230	145
8230	149
8230	179
8244	87
8244	92
8244	97
8244	100
8244	106
8244	137
8252	81
8252	87
8252	96
8252	97
8252	101
8252	102
8252	104
8252	111
8252	117
8252	127
8252	129
8252	147
8268	97
8268	107
8268	114
8268	115
8268	.
8268	.
8313	77
8313	82
8313	83
8313	83
8313	108
8313	.
8339	102
8339	110
8339	111
8339	124
8339	125
8339	183
8372	92
8372	113
8372	115
8372	121
8372	126
8372	129

Cow	Cholesterol
8375	62
8375	74
8375	77
8375	92
8375	120
8375	130
8376	108
8376	109
8376	110
8376	123
8376	131
8376	.
8389	90
8389	91
8389	97
8389	97
8389	116
8389	.
8393	122
8393	133
8393	135
8393	141
8393	142
8393	145
8398	95
8398	117
8398	120
8398	121
8398	144
8398	.
8401	79
8401	81
8401	88
8401	100
8401	101
8401	.
8402	84
8402	88
8402	95
8402	99
8402	107
8402	.
8414	74
8414	80
8414	82
8414	89
8414	104
8414	121

Cow	Cholesterol
8469	124
8469	147
8469	153
8469	155
8469	156
8469	157
8510	92
8510	110
8510	131
8510	136
8510	144
8510	173
8522	97
8522	106
8522	110
8522	110
8522	122
8522	144
8599	92
8599	102
8599	106
8599	122
8599	129
8599	152
8684	61
8684	77
8684	78
8684	79
8684	86
8684	102
8704	115
8704	116
8704	116
8704	121
8704	144
8704	146
8706	70
8706	95
8706	99
8706	119
8706	.
8706	.
9004	116
9004	119
9004	124
9004	132
9004	141
9004	191

Cow	Cholesterol
9027	91
9027	94
9027	96
9027	100
9027	119
9027	.
9028	106
9028	109
9028	112
9028	115
9028	121
9028	159
9030	86
9030	94
9030	98
9030	102
9030	111
9030	148
9031	11
9031	85
9031	105
9031	125
9031	158
9031	182
9060	89
9060	99
9060	101
9060	121
9060	124
9060	163
9066	93
9066	99
9066	104
9066	116
9066	129
9066	163
9074	114
9074	143
9074	146
9074	153
9074	169
9074	172
9087	86
9087	87
9087	88
9087	99
9087	110
9087	.

Cow	Cholesterol
9092	85
9092	95
9092	96
9092	112
9092	132
9092	143
9101	100
9101	130
9101	131
9101	138
9101	144
9101	178
9112	126
9112	161
9112	164
9112	170
9112	176
9112	244
9123	119
9123	133
9123	160
9123	166
9123	167
9123	235
9128	94
9128	128
9128	134
9128	143
9128	165
9128	201
9132	107
9132	118
9132	128
9132	133
9132	151
9132	159
9141	12
9141	13
9141	94
9141	109
9141	115
9141	122
9141	124
9141	158
9141	170
9141	175
9141	182
9141	217

Cow	Cholesterol
9147	68
9147	98
9147	100
9147	109
9147	110
9147	146
9160	106
9160	111
9160	120
9160	127
9160	133
9160	139
9166	115
9166	143
9166	169
9166	.
9166	.
9166	.
9167	136
9167	143
9167	143
9167	146
9167	152
9167	204
9168	72
9168	86
9168	105
9168	106
9168	112
9168	136
9178	66
9178	77
9178	83
9178	85
9178	89
9178	93
9178	94
9178	98
9178	100
9178	102
9178	127
9178	137
9181	81
9181	126
9181	126
9181	142
9181	143
9181	.

Cow	Cholesterol
9194	92
9194	109
9194	110
9194	119
9194	123
9194	.
9204	81
9204	108
9204	118
9204	119
9204	126
9204	155
9210	102
9210	103
9210	111
9210	126
9210	128
9210	151
9211	83
9211	101
9211	102
9211	104
9211	108
9211	136
9218	73
9218	74
9218	75
9218	84
9218	86
9218	.
9226	98
9226	98
9226	99
9226	114
9226	116
9226	.
9228	124
9228	127
9228	137
9228	154
9228	158
9228	.
9229	95
9229	97
9229	112
9229	115
9229	123
9229	.

Cow	Cholesterol
9290	78
9290	82
9290	98
9290	100
9290	116
9290	.
9313	87
9313	112
9313	120
9313	133
9313	133
9313	166
9341	92
9341	121
9341	123
9341	125
9341	140
9341	.
9352	99
9352	125
9352	133
9352	137
9352	151
9352	186
9358	104
9358	112
9358	115
9358	134
9358	144
9358	.
9359	115
9359	132
9359	154
9359	154
9359	162
9359	194
9365	66
9365	76
9365	81
9365	84
9365	109
9365	126
9375	86
9375	119
9375	122
9375	126
9375	127
9375	131

Cow	Cholesterol
9377	70
9377	73
9377	76
9377	85
9377	111
9377	111
9435	62
9435	64
9435	66
9435	76
9435	80
9435	86
9587	110
9587	119
9587	120
9587	124
9587	133
9587	150
9594	47
9594	52
9594	56
9594	58
9594	87
9594	88
9929	68
9929	73
9929	93
9929	100
9929	103
9929	.
9948	123
9948	124
9948	137
9948	141
9948	155
9948	172
51008	102
51008	109
51008	111
51008	122
51008	147
51008	.
51108	118
51108	135
51108	138
51108	163
51108	166
51108	206

Cow	Cholesterol
55112	49
55112	49
55112	54
55112	62
55112	64
55112	.

Cow	group	Time	Milk Kg	Fat %	Protein %
28	Ins	month 1	32	4.42	2.86
28	Ins	month 2	38.4	3.91	2.55
28	Ins	month 3	48.2	2.41	2.7
28	Ins	month 4	51.2	2.84	2.83
28	Ins	month 5	47	2.96	2.77
28	Ins	month 6	45.8	2.73	2.85
47	Con	month 1	.	.	.
47	Con	month 2	.	.	.
47	Con	month 3	.	.	.
47	Con	month 4	.	.	.
47	Con	month 5	.	.	.
47	Con	month 6	.	.	.
56	Con	month 1	15.4	6.77	3.25
56	Con	month 2	46.9	3.66	2.51
56	Con	month 3	49.8	2.41	2.73
56	Con	month 4	37.6	3.3	2.79
56	Con	month 5	37.5	3.12	2.78
56	Con	month 6	47	3.33	2.69
67	Ins	month 1	44.2	3.37	2.43
67	Ins	month 2	44.2	3.52	2.56
67	Ins	month 3	40.4	3.59	2.6
67	Ins	month 4	41.6	3.09	2.53
67	Ins	month 5	30.9	4.3	2.62
67	Ins	month 6	34.2	3.27	2.75
71	Dex	month 1	38.9	1.95	2.63
71	Dex	month 2	36.4	3.41	2.68
71	Dex	month 3	41.5	3.3	2.5
71	Dex	month 4	43.5	3.33	2.64
71	Dex	month 5	35	3.33	2.32
71	Dex	month 6	43.3	2.82	2.7
78	Ins	month 1	31.2	3.96	2.85
78	Ins	month 2	41	3.4	2.52
78	Ins	month 3	38.1	2.76	2.61
78	Ins	month 4	39.1	3.71	2.62
78	Ins	month 5	43.8	3.62	2.65
78	Ins	month 6	41.8	3.54	2.72
94	Ins+Dex	month 1	24	4.08	2.53
94	Ins+Dex	month 2	29	3.74	2.51
94	Ins+Dex	month 3	29.9	3.32	2.86
94	Ins+Dex	month 4	28.8	3.68	2.88
94	Ins+Dex	month 5	27.2	4.11	2.99
94	Ins+Dex	month 6	29.8	3.93	2.95
95	Con	month 1	36	4.38	3.45
95	Con	month 2	36	5.07	3.52
95	Con	month 3	32	4.93	3.51
95	Con	month 4	30.8	5.09	3.88
95	Con	month 5	29.4	4.77	3.66
95	Con	month 6	29.8	5.16	3.48

Cow	group	Time	Milk Kg	Fat %	Protein %
99	Con	month 1	35	3.87	2.92
99	Con	month 2	39	3.71	3.05
99	Con	month 3	32.9	4.62	3
99	Con	month 4	35.6	4.39	3.17
99	Con	month 5	33.4	4.88	3.01
99	Con	month 6	36.4	4.48	3.23
102	Con	month 1	23.4	2.58	4.07
102	Con	month 2	50.4	3.3	2.38
102	Con	month 3	45.9	3.92	2.76
102	Con	month 4	50.9	3.36	2.74
102	Con	month 5	49.9	3.15	2.74
102	Con	month 6	46.6	3.52	2.64
106	Ins	month 1	29.6	4.94	3.39
106	Ins	month 2	38.6	3.61	2.65
106	Ins	month 3	38.1	3.64	3.35
106	Ins	month 4	36	3.87	2.85
106	Ins	month 5	45.1	3.58	2.98
106	Ins	month 6	30.3	4.52	3.05
107	Ins	month 1	36.4	4.54	2.32
107	Ins	month 2	43.5	2.58	2.6
107	Ins	month 3	41.1	3.24	2.66
107	Ins	month 4	39.6	3.13	2.57
107	Ins	month 5	41.8	3.51	2.66
107	Ins	month 6	40.2	3.27	2.74
110	Ins+Dex	month 1	29.2	5.01	3.2
110	Ins+Dex	month 2	28.6	5.07	2.75
110	Ins+Dex	month 3	30.6	3.86	2.73
110	Ins+Dex	month 4	33	3.27	2.56
110	Ins+Dex	month 5	32.7	3.82	2.71
110	Ins+Dex	month 6	33.6	3.47	3.04
112	Ins	month 1	36.4	2.31	2.37
112	Ins	month 2	45	2.45	2.41
112	Ins	month 3	45.2	2.5	2.55
112	Ins	month 4	47.2	3.01	2.63
112	Ins	month 5	53.6	2.99	2.58
112	Ins	month 6	39.8	2.73	2.6
118	Ins+Dex	month 1	19.5	4.42	2.6
118	Ins+Dex	month 2	28.8	3.48	2.49
118	Ins+Dex	month 3	30.3	2.94	2.63
118	Ins+Dex	month 4	32.4	4.4	2.78
118	Ins+Dex	month 5	26.5	.	.
118	Ins+Dex	month 6	32.5	4.66	2.91
120	Con	month 1	21.5	2.75	2.83
120	Con	month 2	27.9	4.6	2.7
120	Con	month 3	30.7	6.08	2.76
120	Con	month 4	26.4	6.26	2.91
120	Con	month 5	26	3.83	3
120	Con	month 6	28.2	7.2	3.14

Cow	group	Time	Milk Kg	Fat %	Protein %
132	Ins	month 1	29.2	3.95	2.99
132	Ins	month 2	36.6	2.97	2.57
132	Ins	month 3	38.3	4.13	2.55
132	Ins	month 4	37.3	3.36	2.76
132	Ins	month 5	36.7	2.85	2.78
132	Ins	month 6	29.5	3.7	2.56
147	Dex	month 1	43.2	3.57	2.6
147	Dex	month 2	47.4	2.12	2.61
147	Dex	month 3	52	2.28	2.49
147	Dex	month 4	50.8	3.02	2.92
147	Dex	month 5	52	3.25	2.76
147	Dex	month 6	47.4	3.02	2.86
151	Ins+Dex	month 1	41.4	4.25	3.28
151	Ins+Dex	month 2	50.3	5.37	2.74
151	Ins+Dex	month 3	49.1	3.07	2.93
151	Ins+Dex	month 4	42	4.38	2.98
151	Ins+Dex	month 5	44.1	3.69	3.06
151	Ins+Dex	month 6	39.7	5.36	3.22
186	Con	month 1	34.6	2.89	2.68
186	Con	month 2	37.2	4.2	2.49
186	Con	month 3	36.2	2.9	2.61
186	Con	month 4	38.4	3.87	2.66
186	Con	month 5	38	4.22	2.72
186	Con	month 6	41.2	3.13	2.77
188	Ins	month 1	28.2	5.15	3.25
188	Ins	month 2	43.6	4.24	2.42
188	Ins	month 3	46.4	3.64	2.42
188	Ins	month 4	37.6	3.46	2.99
188	Ins	month 5	40.2	2.79	2.81
188	Ins	month 6	41.8	3.71	2.72
190	Ins+Dex	month 1	32.4	3.44	2.68
190	Ins+Dex	month 2	41.2	2.44	2.89
190	Ins+Dex	month 3	41.4	3.76	2.84
190	Ins+Dex	month 4	41.4	3.18	2.97
190	Ins+Dex	month 5	43.4	4.09	3
190	Ins+Dex	month 6	41.4	3.67	3.15
208	Con	month 1	28.4	2.74	2.56
208	Con	month 2	23	15.8	1.86
208	Con	month 3	.	.	.
208	Con	month 4	.	.	.
208	Con	month 5	.	.	.
208	Con	month 6	.	.	.
224	Dex	month 1	24.6	3.65	3.45
224	Dex	month 2	26.2	1.78	2.64
224	Dex	month 3	42.2	1.88	2.45
224	Dex	month 4	45.4	3	2.54
224	Dex	month 5	45.2	3.51	2.76
224	Dex	month 6	47.8	2.98	2.65

Cow	group	Time	Milk Kg	Fat %	Protein %		
225	Con	month 1	23	4.83	3.2		
225	Con	month 2	47.6	2.45	2.41		
225	Con	month 3	51	1.52	2.36		
225	Con	month 4	55.4	2.29	2.41		
225	Con	month 5	58.4	2.57	2.37		
225	Con	month 6	59	2.25	2.43		
231	Dex	month 1	20	1.84	2.73		
231	Dex	month 2	35.2	3.78	2.54		
231	Dex	month 3	36.8	3.11	2.63		
231	Dex	month 4	36.2	3.58	2.78		
231	Dex	month 5	36.8	3.68	2.79		
231	Dex	month 6	33	3.66	2.9		
245	Dex	month 1	34.8	3.53	2.65		
245	Dex	month 2	37	3.95	2.53		
245	Dex	month 3	42.2	2.92	2.77		
245	Dex	month 4	40	8.67	2.4		
245	Dex	month 5	43.2	3.23	2.93		
245	Dex	month 6	38.2	3.28	3		
247	Ins	month 1	36.4	2.97	2.62		
247	Ins	month 2	38.2	2.93	2.62		
247	Ins	month 3	40.2	2.84	2.58		
247	Ins	month 4	40	3.3	2.64		
247	Ins	month 5	45.8	2.76	2.6		
247	Ins	month 6	42	2.57	2.88		
248	Dex	month 1	21.2	3.25	2.69		
248	Dex	month 2	14	3.74	2.84		
248	Dex	month 3	19	2.21	2.91		
248	Dex	month 4	32	3.96	2.93		
248	Dex	month 5	34.4	3.5	2.98		
248	Dex	month 6	33.2	3.46	3.1		
249	Ins	month 1	23.6	3.84	2.78		
249	Ins	month 2	36.2	3.36	2.67		
249	Ins	month 3	34	3.38	2.66		
249	Ins	month 4	37	3.68	3.01		
249	Ins	month 5	33	4.72	3.24		
249	Ins	month 6	35.2	3.79	3.02		
256	Dex	month 1	34.6	4.31	2.61		
256	Dex	month 2	41.2	2.73	2.62		
256	Dex	month 3	39	3.47	2.73		
256	Dex	month 4	42	2.9	2.81		
256	Dex	month 5	46.2	3.74	2.7		
256	Dex	month 6	38.8	3.61	2.78		
261	Ins+Dex	month 1	24.6	2.96	2.76		
261	Ins+Dex	month 2	36.4	1.65	2.56		
261	Ins+Dex	month 3	37	3.34	2.56		
261	Ins+Dex	month 4	43.4	2.45	2.76		
261	Ins+Dex	month 5	43.8	3.38	2.77		
261	Ins+Dex	month 6	40.2	3.31	2.7		

Cow	group	Time	Milk Kg	Fat %	Protein %
329	Dex	month 1	33	2.56	3
329	Dex	month 2	42.8	3.99	2.53
329	Dex	month 3	33.8	4.13	2.73
329	Dex	month 4	27.5	4.61	2.56
329	Dex	month 5	35.1	3.56	2.71
329	Dex	month 6	31.5	2.89	2.78
337	Con	month 1	30.8	4.16	3.23
337	Con	month 2	39.2	3.4	2.72
337	Con	month 3	32.8	3.22	2.75
337	Con	month 4	36	3.28	2.85
337	Con	month 5	31.8	3.09	2.89
337	Con	month 6	17.7	3.64	2.78
524	Con	month 1	29	6.9	3.54
524	Con	month 2	53.6	3.46	2.41
524	Con	month 3	22.6	.	.
524	Con	month 4	.	.	.
524	Con	month 5	.	.	.
524	Con	month 6	.	.	.
662	Ins	month 1	17.9	1.39	3.13
662	Ins	month 2	34.2	4.15	2.99
662	Ins	month 3	40.4	4.34	3.06
662	Ins	month 4	35.2	4.53	2.99
662	Ins	month 5	25.8	4.5	2.68
662	Ins	month 6	33.4	4.68	2.99
731	Ins	month 1	32.1	7.04	3.28
731	Ins	month 2	.	.	.
731	Ins	month 3	.	.	.
731	Ins	month 4	.	.	.
731	Ins	month 5	.	.	.
731	Ins	month 6	.	.	.
813	Ins+Dex	month 1	20.4	1.22	2.85
813	Ins+Dex	month 2	40.8	2.03	2.53
813	Ins+Dex	month 3	36.8	3.68	2.72
813	Ins+Dex	month 4	37.6	2.75	2.94
813	Ins+Dex	month 5	36.4	3.84	3.02
813	Ins+Dex	month 6	36	4.45	2.97
856	Con	month 1	67.6	3.02	2.55
856	Con	month 2	56.4	2.88	2.83
856	Con	month 3	.	.	.
856	Con	month 4	.	.	.
856	Con	month 5	.	.	.
856	Con	month 6	.	.	.
938	Dex	month 1	13.4	4.18	3.38
938	Dex	month 2	33.8	2.93	2.68
938	Dex	month 3	37	2.94	2.76
938	Dex	month 4	34.6	3.03	2.67
938	Dex	month 5	36.9	2.47	2.74
938	Dex	month 6	32.8	2.94	2.51

Cow	group	Time	Milk Kg	Fat %	Protein %
944	Ins+Dex	month 1	42.8	4.34	3.37
944	Ins+Dex	month 2	57	4.05	2.7
944	Ins+Dex	month 3	54.6	4.31	2.75
944	Ins+Dex	month 4	50	5.82	2.83
944	Ins+Dex	month 5	53.4	3.98	2.77
944	Ins+Dex	month 6	53.6	4.81	2.77
1281	Ins+Dex	month 1	10.2	.	.
1281	Ins+Dex	month 2	17.8	.	.
1281	Ins+Dex	month 3	.	.	.
1281	Ins+Dex	month 4	.	.	.
1281	Ins+Dex	month 5	.	.	.
1281	Ins+Dex	month 6	.	.	.
2493	Dex	month 1	39.5	4.97	3.53
2493	Dex	month 2	52	3.89	2.52
2493	Dex	month 3	45.3	4.45	2.59
2493	Dex	month 4	47.8	3.25	2.89
2493	Dex	month 5	46.6	4.07	3.05
2493	Dex	month 6	44.6	4.61	3.2
2624	Con	month 1	56.4	4.9	2.72
2624	Con	month 2	58.7	3.71	2.72
2624	Con	month 3	36.3	3.9	2.69
2624	Con	month 4	45.2	3.08	2.85
2624	Con	month 5	43	3.41	2.93
2624	Con	month 6	42.8	3.79	2.8
2694	Ins+Dex	month 1	44.8	4.13	2.93
2694	Ins+Dex	month 2	52.1	3.39	3.11
2694	Ins+Dex	month 3	60.8	3.03	2.78
2694	Ins+Dex	month 4	51.6	3.55	2.79
2694	Ins+Dex	month 5	46.7	3.72	2.76
2694	Ins+Dex	month 6	.	.	.
2741	Con	month 1	34.8	4.31	3.34
2741	Con	month 2	52	2.86	2.64
2741	Con	month 3	50.5	3.86	2.81
2741	Con	month 4	42.6	3.72	2.73
2741	Con	month 5	47.8	2.9	2.76
2741	Con	month 6	38.3	3.47	2.91
2764	Ins	month 1	59.2	3.83	2.57
2764	Ins	month 2	54.1	3.33	2.92
2764	Ins	month 3	36.8	6.64	2.91
2764	Ins	month 4	42.5	3.93	3.13
2764	Ins	month 5	44.6	3.51	3.12
2764	Ins	month 6	46.6	3.78	3.01
2813	Con	month 1	17.7	3.38	2.83
2813	Con	month 2	32.4	3.33	2.75
2813	Con	month 3	37.7	3.38	2.97
2813	Con	month 4	36.4	3.6	2.75
2813	Con	month 5	35.2	3.64	2.91
2813	Con	month 6	38.2	3.43	2.88

Cow	group	Time	Milk Kg	Fat %	Protein %		
2940	Con	month 1	.	.	.		
2940	Con	month 2	.	.	.		
2940	Con	month 3	.	.	.		
2940	Con	month 4	.	.	.		
2940	Con	month 5	.	.	.		
2940	Con	month 6	.	.	.		
3526	Dex	month 1	46	3.53	2.81		
3526	Dex	month 2	53	4.02	2.72		
3526	Dex	month 3	50.3	4.38	2.78		
3526	Dex	month 4	57.3	4.32	2.78		
3526	Dex	month 5	46.5	3.08	2.84		
3526	Dex	month 6	46.4	3.41	3.04		
4019	Dex	month 1	31.2	3.81	3		
4019	Dex	month 2	36.6	4.63	3.11		
4019	Dex	month 3	36.5	4.07	3.36		
4019	Dex	month 4	35.3	5.01	3.17		
4019	Dex	month 5	28.6	2.79	2.49		
4019	Dex	month 6	28.1	4.71	3.28		
4044	Ins+Dex	month 1	41.4	3.08	2.78		
4044	Ins+Dex	month 2	40.9	4.57	2.41		
4044	Ins+Dex	month 3	35.7	5.83	2.43		
4044	Ins+Dex	month 4	21.8	4.24	2.96		
4044	Ins+Dex	month 5	20.6	4.48	2.89		
4044	Ins+Dex	month 6	8.8	5.78	3.38		
4053	Ins	month 1	15	1.78	9.2		
4053	Ins	month 2	45.8	4.57	2.75		
4053	Ins	month 3	44.4	3.66	2.81		
4053	Ins	month 4	39.2	2.68	2.92		
4053	Ins	month 5	45	4.23	3.05		
4053	Ins	month 6	43.2	2.77	3.07		
5061	Dex	month 1	21.2	4.34	3.01		
5061	Dex	month 2	24.2	.	.		
5061	Dex	month 3	29	2.93	2.75		
5061	Dex	month 4	46.4	4.26	2.82		
5061	Dex	month 5	46.5	2.8	2.81		
5061	Dex	month 6	42.3	3.52	2.66		
5104	Dex	month 1	27.2	2.61	2.61		
5104	Dex	month 2	.	.	.		
5104	Dex	month 3	.	.	.		
5104	Dex	month 4	.	.	.		
5104	Dex	month 5	.	.	.		
5104	Dex	month 6	.	.	.		
5165	Con	month 1	39	3.38	3.1		
5165	Con	month 2	44.3	3.6	2.63		
5165	Con	month 3	42	2.78	2.68		
5165	Con	month 4	45.6	4.27	2.74		
5165	Con	month 5	45.9	3.86	2.72		
5165	Con	month 6	44.2	3.61	2.71		

Cow	group	Time	Milk Kg	Fat %	Protein %		
5170	Ins	month 1	54.8	2.24	2.75		
5170	Ins	month 2	61.4	3.87	2.42		
5170	Ins	month 3	64	3.44	2.55		
5170	Ins	month 4	.	.	.		
5170	Ins	month 5	.	.	.		
5170	Ins	month 6	.	.	.		
5254	Ins	month 1	46.4	3.68	2.62		
5254	Ins	month 2	.	.	.		
5254	Ins	month 3	.	.	.		
5254	Ins	month 4	.	.	.		
5254	Ins	month 5	.	.	.		
5254	Ins	month 6	.	.	.		
5371	Ins	month 1	28.8	5.38	3.62		
5371	Ins	month 2	45.4	3.47	2.91		
5371	Ins	month 3	45.6	3.96	2.9		
5371	Ins	month 4	.	.	.		
5371	Ins	month 5	.	.	.		
5371	Ins	month 6	.	.	.		
5397	Ins	month 1	25.6	3.56	3.93		
5397	Ins	month 2	44.1	3.88	2.76		
5397	Ins	month 3	43.8	3.66	2.84		
5397	Ins	month 4	42.6	3.86	2.86		
5397	Ins	month 5	43.2	3.13	3.02		
5397	Ins	month 6	39.2	4.36	3.02		
5405	Ins+Dex	month 1	33.3	3.57	2.97		
5405	Ins+Dex	month 2	39.9	3.25	2.95		
5405	Ins+Dex	month 3	32.4	3.29	2.93		
5405	Ins+Dex	month 4	18.6	.	.		
5405	Ins+Dex	month 5	33.8	2.8	2.84		
5405	Ins+Dex	month 6	31.4	3.49	3.05		
5415	Ins+Dex	month 1	29.7	3.75	2.29		
5415	Ins+Dex	month 2	45.4	3.9	2.72		
5415	Ins+Dex	month 3	49.4	2.83	2.75		
5415	Ins+Dex	month 4	48.6	3.16	2.86		
5415	Ins+Dex	month 5	.	.	.		
5415	Ins+Dex	month 6	.	.	.		
5771	Dex	month 1	27.5	2.78	2.68		
5771	Dex	month 2	60.7	3.99	2.71		
5771	Dex	month 3	32.6	3.49	2.98		
5771	Dex	month 4	31.8	3.14	2.88		
5771	Dex	month 5	32.6	3.97	2.82		
5771	Dex	month 6	35.7	3	2.94		
5919	Con	month 1	27.4	3.64	2.73		
5919	Con	month 2	44.1	3.48	2.65		
5919	Con	month 3	41.4	3.86	2.85		
5919	Con	month 4	36.5	4	2.85		
5919	Con	month 5	35.3	3.17	2.8		
5919	Con	month 6	31.9	3.38	3.03		

Cow	group	Time	Milk Kg	Fat %	Protein %
5971	Ins+Dex	month 1	32.7	4.93	3.28
5971	Ins+Dex	month 2	46	3.47	2.36
5971	Ins+Dex	month 3	44.1	2.86	2.65
5971	Ins+Dex	month 4	44.6	2.92	2.65
5971	Ins+Dex	month 5	38.1	3.32	2.81
5971	Ins+Dex	month 6	38.9	3.61	2.84
6052	Ins+Dex	month 1	34.5	4.51	3.76
6052	Ins+Dex	month 2	46.5	4.33	2.59
6052	Ins+Dex	month 3	47.9	2.16	2.67
6052	Ins+Dex	month 4	40	4.5	2.8
6052	Ins+Dex	month 5	38.2	3.46	2.86
6052	Ins+Dex	month 6	39.2	2.62	2.92
6123	Dex	month 1	.	.	.
6123	Dex	month 2	.	.	.
6123	Dex	month 3	.	.	.
6123	Dex	month 4	.	.	.
6123	Dex	month 5	.	.	.
6123	Dex	month 6	.	.	.
6145	Dex	month 1	30.8	3.49	2.3
6145	Dex	month 2	52.8	3.08	2.27
6145	Dex	month 3	48.8	.	.
6145	Dex	month 4	48.8	2.89	2.41
6145	Dex	month 5	45.8	3.6	2.38
6145	Dex	month 6	43.9	3.21	2.45
6203	Dex	month 1	40.2	5.15	2.75
6203	Dex	month 2	53	4.44	2.53
6203	Dex	month 3	32.2	2.75	2.69
6203	Dex	month 4	39.8	2.51	2.87
6203	Dex	month 5	31.5	1.97	2.94
6203	Dex	month 6	25.4	3.74	2.82
6220	Ins+Dex	month 1	27.6	4.72	3.67
6220	Ins+Dex	month 2	37	5.08	2.89
6220	Ins+Dex	month 3	46.1	.	.
6220	Ins+Dex	month 4	42.5	5.09	2.95
6220	Ins+Dex	month 5	21.7	4.93	3.04
6220	Ins+Dex	month 6	43.2	4.34	3.08
6284	Ins+Dex	month 1	53	3.04	2.49
6284	Ins+Dex	month 2	54.4	2.24	2.58
6284	Ins+Dex	month 3	55	2.13	2.75
6284	Ins+Dex	month 4	46.4	3.49	3
6284	Ins+Dex	month 5	45	3.48	3
6284	Ins+Dex	month 6	35.4	1.11	3.05
6515	Con	month 1	22.4	6.17	4.1
6515	Con	month 2	.	.	.
6515	Con	month 3	.	.	.
6515	Con	month 4	.	.	.
6515	Con	month 5	.	.	.
6515	Con	month 6	.	.	.

Cow	group	Time	Milk Kg	Fat %	Protein %		
6540	Ins	month 1	36	3.87	3.78		
6540	Ins	month 2	54	2.99	2.5		
6540	Ins	month 3	44	3.64	2.74		
6540	Ins	month 4	33.4	4.15	3.2		
6540	Ins	month 5	32	4.9	3.38		
6540	Ins	month 6	24.8	4.95	3.29		
6724	Ins	month 1	48	4.13	2.92		
6724	Ins	month 2	44.7	4.46	2.97		
6724	Ins	month 3	40.6	5.17	2.93		
6724	Ins	month 4	40.6	5.08	3.08		
6724	Ins	month 5	38.7	4.79	3.15		
6724	Ins	month 6	38.4	4.54	3.07		
7048	Ins	month 1	26	5.32	3.55		
7048	Ins	month 2	52.4	3.04	2.76		
7048	Ins	month 3	67	2.42	2.65		
7048	Ins	month 4	66	1.85	2.77		
7048	Ins	month 5	66.6	3.38	2.74		
7048	Ins	month 6	63.4	3.88	2.79		
7050	Con	month 1	55.4	3.69	2.51		
7050	Con	month 2	65.6	2.51	2.4		
7050	Con	month 3	40.8	1.63	2.84		
7050	Con	month 4	47	3.76	2.68		
7050	Con	month 5	44	4.54	2.6		
7050	Con	month 6	40.6	4.83	2.66		
7067	Ins+Dex	month 1	39.8	4.19	2.69		
7067	Ins+Dex	month 2	38.6	3.16	2.87		
7067	Ins+Dex	month 3	37.6	4.09	3.09		
7067	Ins+Dex	month 4	39.8	5.06	2.98		
7067	Ins+Dex	month 5	37.2	4.05	3.09		
7067	Ins+Dex	month 6	42.4	5.83	2.99		
7068	Dex	month 1	49.8	3.55	2.66		
7068	Dex	month 2	56	1.62	2.74		
7068	Dex	month 3	51	1.73	2.92		
7068	Dex	month 4	45.4	2.76	2.99		
7068	Dex	month 5	14	4.61	3.5		
7068	Dex	month 6	36.4	2.63	2.93		
7113	Dex	month 1	56.4	2.59	2.55		
7113	Dex	month 2	72	3.77	2.41		
7113	Dex	month 3	73	3.19	2.6		
7113	Dex	month 4	56	1.56	2.81		
7113	Dex	month 5	69.2	2.64	2.75		
7113	Dex	month 6	68.2	3.01	2.58		
7121	Ins+Dex	month 1	38.2	.	.		
7121	Ins+Dex	month 2	30.9	5.54	4		
7121	Ins+Dex	month 3	38	2.51	2.84		
7121	Ins+Dex	month 4	38.6	3.01	2.91		
7121	Ins+Dex	month 5	38.6	4.2	2.82		
7121	Ins+Dex	month 6	39.1	4.24	2.81		

Cow	group	Time	Milk Kg	Fat %	Protein %
7133	Ins+Dex	month 1	38.4	3.01	2.49
7133	Ins+Dex	month 2	.	.	.
7133	Ins+Dex	month 3	.	.	.
7133	Ins+Dex	month 4	.	.	.
7133	Ins+Dex	month 5	.	.	.
7133	Ins+Dex	month 6	.	.	.
7136	Ins	month 1	13.4	4.58	2.64
7136	Ins	month 2	.	.	.
7136	Ins	month 3	.	.	.
7136	Ins	month 4	.	.	.
7136	Ins	month 5	.	.	.
7136	Ins	month 6	.	.	.
7140	Ins+Dex	month 1	20.4	11	3.79
7140	Ins+Dex	month 2	.	.	.
7140	Ins+Dex	month 3	.	.	.
7140	Ins+Dex	month 4	.	.	.
7140	Ins+Dex	month 5	.	.	.
7140	Ins+Dex	month 6	.	.	.
7141	Ins	month 1	26.5	4.02	2.78
7141	Ins	month 2	28.7	2.91	2.75
7141	Ins	month 3	34.8	2.95	3.07
7141	Ins	month 4	19.2	4.07	3.04
7141	Ins	month 5	21.4	4.04	3.3
7141	Ins	month 6	19.3	4	3.15
7151	Ins+Dex	month 1	39.8	3.09	2.67
7151	Ins+Dex	month 2	32.4	1.89	2.97
7151	Ins+Dex	month 3	35.2	3.86	2.93
7151	Ins+Dex	month 4	40.1	2.61	2.86
7151	Ins+Dex	month 5	23.7	4.01	2.78
7151	Ins+Dex	month 6	31.6	3.19	2.88
7157	Con	month 1	33.8	2.4	2.69
7157	Con	month 2	35.2	2.4	2.52
7157	Con	month 3	30	3.81	3.01
7157	Con	month 4	32	3.38	2.95
7157	Con	month 5	32.4	2.2	3.28
7157	Con	month 6	28.8	3.87	2.92
7177	Con	month 1	23.4	4.79	3.79
7177	Con	month 2	16.2	4.46	2.74
7177	Con	month 3	7.4	6	3.08
7177	Con	month 4	5.6	4.06	3.26
7177	Con	month 5	3.2	2.83	3.19
7177	Con	month 6	.	.	.
7194	Ins+Dex	month 1	7.6	8.13	5.88
7194	Ins+Dex	month 2	.	.	.
7194	Ins+Dex	month 3	.	.	.
7194	Ins+Dex	month 4	.	.	.
7194	Ins+Dex	month 5	.	.	.
7194	Ins+Dex	month 6	.	.	.

Cow	group	Time	Milk Kg	Fat %	Protein %
7268	Dex	month 1	54.6	3.39	2.82
7268	Dex	month 2	58	3.91	2.67
7268	Dex	month 3	54.6	2.88	2.75
7268	Dex	month 4	55.4	3.02	2.99
7268	Dex	month 5	53.2	3.5	3.03
7268	Dex	month 6	45	3.64	3.07
7322	Ins+Dex	month 1	21.6	3.89	2.92
7322	Ins+Dex	month 2	.	.	.
7322	Ins+Dex	month 3	.	.	.
7322	Ins+Dex	month 4	.	.	.
7322	Ins+Dex	month 5	.	.	.
7322	Ins+Dex	month 6	.	.	.
7353	Ins	month 1	50.4	1.77	2.71
7353	Ins	month 2	48.4	3.34	2.48
7353	Ins	month 3	56.6	3.51	2.64
7353	Ins	month 4	52.4	3.59	2.8
7353	Ins	month 5	54.6	3.8	2.78
7353	Ins	month 6	43.6	3.19	2.74
7368	Con	month 1	48.4	3.69	3.53
7368	Con	month 2	57	2.67	2.57
7368	Con	month 3	58	2.71	2.93
7368	Con	month 4	61.6	3.54	2.97
7368	Con	month 5	.	.	.
7368	Con	month 6	.	.	.
7403	Con	month 1	41.8	3.15	2.8
7403	Con	month 2	62	2.54	2.57
7403	Con	month 3	46	3.03	2.53
7403	Con	month 4	55.8	2.81	2.67
7403	Con	month 5	56	3.43	2.71
7403	Con	month 6	48.2	2.95	2.77
7412	Dex	month 1	25.2	4.55	2.95
7412	Dex	month 2	61	2.85	2.38
7412	Dex	month 3	60.6	3.71	2.31
7412	Dex	month 4	61	2.22	2.37
7412	Dex	month 5	57.6	3.04	2.29
7412	Dex	month 6	.	.	.
7521	Con	month 1	57	3.02	2.49
7521	Con	month 2	58.8	3.45	2.5
7521	Con	month 3	61.4	4.16	2.64
7521	Con	month 4	59.4	4.05	2.84
7521	Con	month 5	52.4	4.28	2.78
7521	Con	month 6	44.4	2.92	2.82
7537	Ins	month 1	32.2	3.93	3.7
7537	Ins	month 2	45.4	4.73	2.77
7537	Ins	month 3	44.6	4.23	2.79
7537	Ins	month 4	33	5.2	2.98
7537	Ins	month 5	31.8	4.15	3.03
7537	Ins	month 6	31	3.59	2.86

Cow	group	Time	Milk Kg	Fat %	Protein %
7543	Dex	month 1	51.8	2.67	2.51
7543	Dex	month 2	53.4	3.02	2.54
7543	Dex	month 3	60.2	2.56	2.77
7543	Dex	month 4	61.4	3.36	2.79
7543	Dex	month 5	27.6	4.3	3.46
7543	Dex	month 6	.	.	.
7550	Ins	month 1	48.8	3.72	3.04
7550	Ins	month 2	50.6	3.96	2.93
7550	Ins	month 3	52.6	3.01	3
7550	Ins	month 4	48.4	3.82	3.1
7550	Ins	month 5	45.8	4.7	3.04
7550	Ins	month 6	42.6	4.51	3.04
7712	Con	month 1	48.4	3.65	2.52
7712	Con	month 2	50.4	2.87	2.56
7712	Con	month 3	43.4	3.49	2.68
7712	Con	month 4	41.1	3.11	2.87
7712	Con	month 5	39.7	2.75	2.96
7712	Con	month 6	41.4	3.3	2.84
8015	Ins	month 1	41.4	3.35	2.77
8015	Ins	month 2	37.4	4.78	3.85
8015	Ins	month 3	.	.	.
8015	Ins	month 4	.	.	.
8015	Ins	month 5	.	.	.
8015	Ins	month 6	.	.	.
8017	Dex	month 1	49.3	3.96	2.82
8017	Dex	month 2	44.1	3.65	2.82
8017	Dex	month 3	45.2	3.76	2.74
8017	Dex	month 4	42.6	3.04	2.83
8017	Dex	month 5	44.5	3.01	3.03
8017	Dex	month 6	38	4.98	2.86
8027	Dex	month 1	38.5	4.23	2.92
8027	Dex	month 2	60.4	2.46	2.52
8027	Dex	month 3	52.8	2.72	2.57
8027	Dex	month 4	44.2	4.42	2.68
8027	Dex	month 5	47.8	4.47	2.78
8027	Dex	month 6	45.2	2.88	2.94
8040	Ins+Dex	month 1	41.8	4.61	3
8040	Ins+Dex	month 2	53.1	4.48	2.72
8040	Ins+Dex	month 3	48.4	2.84	2.79
8040	Ins+Dex	month 4	47.6	3.63	2.83
8040	Ins+Dex	month 5	47.2	3.5	2.97
8040	Ins+Dex	month 6	45.2	4.59	3.04
8046	Dex	month 1	30.4	2.62	2.95
8046	Dex	month 2	52	3.44	2.64
8046	Dex	month 3	46.6	3.19	2.57
8046	Dex	month 4	52.6	3.35	2.93
8046	Dex	month 5	47	4.59	3.01
8046	Dex	month 6	63	3.18	2.8

Cow	group	Time	Milk Kg	Fat %	Protein %
8047	Ins	month 1	45.6	3.48	3.25
8047	Ins	month 2	45.8	4.34	2.92
8047	Ins	month 3	45.2	3.54	2.83
8047	Ins	month 4	48.4	3.65	2.98
8047	Ins	month 5	46.4	3.84	2.84
8047	Ins	month 6	38.8	3.16	2.98
8082	Con	month 1	45.6	3.21	2.72
8082	Con	month 2	64.8	2.5	2.58
8082	Con	month 3	65.2	2.77	2.68
8082	Con	month 4	61.6	1.77	2.71
8082	Con	month 5	58.2	1.04	2.91
8082	Con	month 6	47.4	2.45	2.69
8086	Ins	month 1	46.6	3.41	2.71
8086	Ins	month 2	48.8	3.57	2.4
8086	Ins	month 3	47.2	3.25	2.82
8086	Ins	month 4	45	3.74	2.8
8086	Ins	month 5	44.2	4.02	2.75
8086	Ins	month 6	34.8	3.86	2.81
8122	Con	month 1	29.2	3.3	3.39
8122	Con	month 2	44.3	3.08	2.55
8122	Con	month 3	36.5	2.61	2.76
8122	Con	month 4	28.7	3.32	2.8
8122	Con	month 5	30	2.94	2.9
8122	Con	month 6	28.3	4.55	2.95
8129	Dex	month 1	43.2	2.93	2.68
8129	Dex	month 2	38.8	2.64	3.79
8129	Dex	month 3	37.4	3.09	2.72
8129	Dex	month 4	43.2	3.06	2.75
8129	Dex	month 5	43.6	3.44	2.68
8129	Dex	month 6	43	2.83	2.78
8133	Ins+Dex	month 1	52.6	3.64	2.51
8133	Ins+Dex	month 2	49.8	3.37	2.68
8133	Ins+Dex	month 3	43.8	2.92	2.88
8133	Ins+Dex	month 4	38.1	4.15	2.91
8133	Ins+Dex	month 5	37.4	3.83	3.18
8133	Ins+Dex	month 6	37.1	6.24	2.88
8152	Dex	month 1	48.5	3.45	2.42
8152	Dex	month 2	62	2.55	2.7
8152	Dex	month 3	44	3.7	2.78
8152	Dex	month 4	41.2	5.18	2.75
8152	Dex	month 5	35	3.98	2.94
8152	Dex	month 6	34	4.47	2.86
8154	Dex	month 1	47.8	2.91	2.72
8154	Dex	month 2	48	2.71	2.62
8154	Dex	month 3	47.4	2.94	2.81
8154	Dex	month 4	41	4.39	3.1
8154	Dex	month 5	40.8	4.02	3
8154	Dex	month 6	31.8	4.99	2.95

Cow	group	Time	Milk Kg	Fat %	Protein %
8156	Ins+Dex	month 1	23.6	2.88	3.79
8156	Ins+Dex	month 2	45.8	2.45	2.64
8156	Ins+Dex	month 3	54.8	3.27	2.85
8156	Ins+Dex	month 4	29	1.71	2.77
8156	Ins+Dex	month 5	44	3.73	3.07
8156	Ins+Dex	month 6	43.6	3.81	2.98
8158	Ins	month 1	40.8	4.02	2.95
8158	Ins	month 2	59.4	3.61	2.48
8158	Ins	month 3	52	2.52	2.64
8158	Ins	month 4	46	3.6	2.74
8158	Ins	month 5	41.3	3.18	3.02
8158	Ins	month 6	44.7	3.26	2.97
8158	Ins+Dex	month 1	48	5.43	2.86
8158	Ins+Dex	month 2	50.4	3.13	2.74
8158	Ins+Dex	month 3	47.8	2.52	2.89
8158	Ins+Dex	month 4	43.6	4.53	3.15
8158	Ins+Dex	month 5	38.6	3.65	3.08
8158	Ins+Dex	month 6	38	4.17	3.18
8160	Dex	month 1	42	4.25	2.93
8160	Dex	month 2	49.8	2.94	3.06
8160	Dex	month 3	42	2.82	3.13
8160	Dex	month 4	34	3.45	3.38
8160	Dex	month 5	32.2	3.83	3.2
8160	Dex	month 6	12.8	4.39	3.44
8177	Ins+Dex	month 1	48.8	3.78	2.69
8177	Ins+Dex	month 2	57.8	4.34	2.51
8177	Ins+Dex	month 3	56.2	3.26	2.6
8177	Ins+Dex	month 4	50	3.46	2.8
8177	Ins+Dex	month 5	44	4.88	2.67
8177	Ins+Dex	month 6	35.8	4.18	3.16
8181	Ins	month 1	38.6	3.25	3.29
8181	Ins	month 2	47.8	3.55	2.63
8181	Ins	month 3	43.4	1.48	2.8
8181	Ins	month 4	43.6	3.69	3.1
8181	Ins	month 5	50.2	3.86	2.66
8181	Ins	month 6	40.6	3.55	2.86
8186	Con	month 1	46.2	3.64	2.63
8186	Con	month 2	48.8	3.7	2.44
8186	Con	month 3	45	1.58	2.66
8186	Con	month 4	50.8	3.9	2.91
8186	Con	month 5	48.6	3.96	2.9
8186	Con	month 6	45.4	3.5	2.9
8221	Con	month 1	40	4.63	3.2
8221	Con	month 2	45.6	3.37	2.85
8221	Con	month 3	48	3.08	2.94
8221	Con	month 4	40.6	3.1	3
8221	Con	month 5	40.2	5.43	3.11
8221	Con	month 6	36.2	4.91	3.15

Cow	group	Time	Milk Kg	Fat %	Protein %
8230	Ins	month 1	54	2.88	2.63
8230	Ins	month 2	34.6	3.17	2.27
8230	Ins	month 3	36	2.81	2.49
8230	Ins	month 4	43.4	2.7	2.83
8230	Ins	month 5	41.4	3.97	2.83
8230	Ins	month 6	36.8	3.12	2.77
8244	Ins	month 1	53.2	3.76	2.59
8244	Ins	month 2	65	3.12	2.53
8244	Ins	month 3	65.4	2.48	2.65
8244	Ins	month 4	68	3.43	2.73
8244	Ins	month 5	51.4	3.31	2.63
8244	Ins	month 6	57	2.59	2.66
8252	Ins	month 1	54.5	3.27	2.37
8252	Ins	month 2	56	2.62	2.6
8252	Ins	month 3	52	2.69	2.45
8252	Ins	month 4	46.2	3.92	2.43
8252	Ins	month 5	55.1	3.62	2.41
8252	Ins	month 6	36.1	.	.
8252	Dex	month 1	58.4	1.73	2.61
8252	Dex	month 2	61	1.88	2.81
8252	Dex	month 3	52	1.95	2.97
8252	Dex	month 4	42.4	2.88	3.18
8252	Dex	month 5	48	2.53	3.01
8252	Dex	month 6	45.8	2.13	3.02
8268	Dex	month 1	46.4	4	2.39
8268	Dex	month 2	54	2.65	2.34
8268	Dex	month 3	56.8	2.19	2.44
8268	Dex	month 4	51.6	4.26	2.66
8268	Dex	month 5	48.6	3.24	2.69
8268	Dex	month 6	41	2.71	2.75
8313	Ins	month 1	64.6	2.61	2.75
8313	Ins	month 2	37.2	2.41	3.11
8313	Ins	month 3	46.6	3.29	2.96
8313	Ins	month 4	46	2.15	3.14
8313	Ins	month 5	47	3.27	3.06
8313	Ins	month 6	41	3.23	3.09
8339	Dex	month 1	51	5.13	2.72
8339	Dex	month 2	50	3.53	2.29
8339	Dex	month 3	50.6	2.65	2.62
8339	Dex	month 4	25	2.16	2.74
8339	Dex	month 5	51.4	3.52	2.82
8339	Dex	month 6	47.6	3.39	2.76
8372	Ins+Dex	month 1	45	1.87	2.63
8372	Ins+Dex	month 2	54.8	2.44	2.43
8372	Ins+Dex	month 3	53.6	1.32	2.74
8372	Ins+Dex	month 4	48.2	2.6	2.76
8372	Ins+Dex	month 5	40.8	2.32	2.7
8372	Ins+Dex	month 6	35	3	2.88

Cow	group	Time	Milk Kg	Fat %	Protein %
8375	Con	month 1	28.2	2.59	2.79
8375	Con	month 2	59.4	3.09	2.54
8375	Con	month 3	63.6	3.26	2.44
8375	Con	month 4	59.4	2.63	2.77
8375	Con	month 5	56.4	3.49	2.77
8375	Con	month 6	52.8	2.81	2.61
8376	Con	month 1	59	3.95	2.79
8376	Con	month 2	62.4	3.39	2.57
8376	Con	month 3	59.6	2.65	2.63
8376	Con	month 4	59	3.32	2.85
8376	Con	month 5	56.2	2.82	2.79
8376	Con	month 6	50.4	3.32	2.87
8389	Dex	month 1	47.8	4.26	3.25
8389	Dex	month 2	67.6	1.86	2.63
8389	Dex	month 3	62	2.13	2.64
8389	Dex	month 4	65	2.43	2.79
8389	Dex	month 5	65	2.47	2.82
8389	Dex	month 6	57.8	3.34	2.79
8393	Dex	month 1	37.4	4.14	2.75
8393	Dex	month 2	45	3.69	2.43
8393	Dex	month 3	46.6	3.47	2.76
8393	Dex	month 4	43.4	3.02	2.85
8393	Dex	month 5	44.2	3.89	2.76
8393	Dex	month 6	40.2	3.32	2.74
8398	Ins	month 1	52.8	3.2	2.54
8398	Ins	month 2	55.6	2.15	2.41
8398	Ins	month 3	48	2.91	2.64
8398	Ins	month 4	50.8	3.73	2.73
8398	Ins	month 5	49.8	2.45	2.79
8398	Ins	month 6	45	3.12	2.74
8401	Ins+Dex	month 1	28.8	2.43	3.38
8401	Ins+Dex	month 2	39	4.75	2.94
8401	Ins+Dex	month 3	34.8	2.1	3.06
8401	Ins+Dex	month 4	27	2.18	3.08
8401	Ins+Dex	month 5	34.8	4.49	2.96
8401	Ins+Dex	month 6	18.2	2.44	3.04
8402	Ins+Dex	month 1	51.4	4.13	2.88
8402	Ins+Dex	month 2	54.8	3.28	2.7
8402	Ins+Dex	month 3	55.2	3.71	2.81
8402	Ins+Dex	month 4	33	3.88	3.12
8402	Ins+Dex	month 5	44.8	2.28	3.23
8402	Ins+Dex	month 6	37.2	3.06	3.11
8414	Ins+Dex	month 1	21	3.79	3.54
8414	Ins+Dex	month 2	40.6	3.15	2.62
8414	Ins+Dex	month 3	42.6	2.63	2.51
8414	Ins+Dex	month 4	40.2	3.56	2.76
8414	Ins+Dex	month 5	36.6	4.12	2.91
8414	Ins+Dex	month 6	34	3.77	2.8

Cow	group	Time	Milk Kg	Fat %	Protein %
8469	Ins+Dex	month 1	23.6	3.16	4.66
8469	Ins+Dex	month 2	52.8	3.01	2.75
8469	Ins+Dex	month 3	34.2	4.12	2.63
8469	Ins+Dex	month 4	28.8	3.38	2.46
8469	Ins+Dex	month 5	.	.	.
8469	Ins+Dex	month 6	.	.	.
8510	Ins	month 1	40.4	3.53	3.09
8510	Ins	month 2	61.4	2.3	2.47
8510	Ins	month 3	58.8	2.63	2.67
8510	Ins	month 4	49	2.64	2.89
8510	Ins	month 5	46.8	4.03	2.64
8510	Ins	month 6	7	4.49	3.34
8522	Con	month 1	49.2	3.83	2.88
8522	Con	month 2	57.4	3.2	2.78
8522	Con	month 3	61.8	2.01	2.8
8522	Con	month 4	61	3.06	3.01
8522	Con	month 5	58.6	4.31	2.8
8522	Con	month 6	47.4	5.25	3.07
8599	Dex	month 1	32.8	4.1	3.21
8599	Dex	month 2	46.4	2.8	2.79
8599	Dex	month 3	42	4.37	2.94
8599	Dex	month 4	35.4	3.81	2.98
8599	Dex	month 5	41.6	3.9	3.11
8599	Dex	month 6	36.6	2.93	3.12
8684	Ins	month 1	42.6	3.84	3.08
8684	Ins	month 2	54.6	2.16	2.52
8684	Ins	month 3	55	2.84	2.68
8684	Ins	month 4	52	2.93	2.9
8684	Ins	month 5	48.8	2.24	3.02
8684	Ins	month 6	52.2	3.58	2.87
8704	Ins	month 1	67.2	2.41	2.86
8704	Ins	month 2	47.4	1.73	2.47
8704	Ins	month 3	51.2	2.62	2.7
8704	Ins	month 4	49.6	3.3	2.77
8704	Ins	month 5	52.8	3.46	2.73
8704	Ins	month 6	46	3.54	2.8
8706	Ins	month 1	48	1.77	2.9
8706	Ins	month 2	54.8	3.19	2.63
8706	Ins	month 3	51.8	3.36	2.72
8706	Ins	month 4	50.4	3.06	2.85
8706	Ins	month 5	48.2	3.38	2.83
8706	Ins	month 6	40.4	3.83	2.74
9004	Con	month 1	39.3	4.22	3.12
9004	Con	month 2	46.9	3.48	2.57
9004	Con	month 3	46.8	7.38	2.76
9004	Con	month 4	50.2	3.32	2.86
9004	Con	month 5	41	4.76	2.92
9004	Con	month 6	44.4	4.26	2.89

Cow	group	Time	Milk Kg	Fat %	Protein %
9027	Dex	month 1	12.2	3.36	3.65
9027	Dex	month 2	32.2	4.52	2.97
9027	Dex	month 3	32	4.8	2.91
9027	Dex	month 4	38.2	2.94	3.2
9027	Dex	month 5	36.4	3.45	3.24
9027	Dex	month 6	38.4	4.03	3.13
9028	Ins	month 1	35.7	3.83	3.25
9028	Ins	month 2	51.9	2.33	2.4
9028	Ins	month 3	53	2.38	2.64
9028	Ins	month 4	46	3.23	2.6
9028	Ins	month 5	36.4	3.41	2.59
9028	Ins	month 6	51	2.72	2.74
9030	Dex	month 1	49.4	3.31	2.72
9030	Dex	month 2	50.2	1.98	2.64
9030	Dex	month 3	45.4	2.96	2.95
9030	Dex	month 4	46.6	3.8	3.13
9030	Dex	month 5	40.4	3.62	3.24
9030	Dex	month 6	39.4	3.84	3.14
9031	Ins	month 1	45	3.37	2.66
9031	Ins	month 2	38	2.63	2.29
9031	Ins	month 3	48.2	1.89	2.75
9031	Ins	month 4	47.8	2.22	2.87
9031	Ins	month 5	44	3.25	2.88
9031	Ins	month 6	40.8	2.86	2.91
9060	Dex	month 1	33.3	3.75	2.45
9060	Dex	month 2	50	3.16	2.15
9060	Dex	month 3	47.8	3.41	2.94
9060	Dex	month 4	43.8	3.22	2.42
9060	Dex	month 5	37.8	4.06	2.54
9060	Dex	month 6	37.6	2.14	2.72
9066	Ins+Dex	month 1	39.4	3.59	3.22
9066	Ins+Dex	month 2	53	3.71	2.44
9066	Ins+Dex	month 3	43.2	3.65	2.59
9066	Ins+Dex	month 4	50.4	3.3	2.73
9066	Ins+Dex	month 5	46.6	3.39	2.98
9066	Ins+Dex	month 6	47	4.4	2.87
9074	Ins+Dex	month 1	37.2	3.34	3.52
9074	Ins+Dex	month 2	44.6	1.99	2.85
9074	Ins+Dex	month 3	36.4	2.88	3.17
9074	Ins+Dex	month 4	38	2.75	3.24
9074	Ins+Dex	month 5	41.4	2.9	3.2
9074	Ins+Dex	month 6	37.6	3.12	3.18
9087	Con	month 1	15.8	10.16	2.81
9087	Con	month 2	38.6	3.76	3.01
9087	Con	month 3	27.6	4.96	3.23
9087	Con	month 4	37.8	2.12	3.21
9087	Con	month 5	34.8	3.58	3.37
9087	Con	month 6	33.2	3.88	3.1

Cow	group	Time	Milk Kg	Fat %	Protein %
9092	Dex	month 1	48.8	3.28	3.27
9092	Dex	month 2	60	3.28	2.58
9092	Dex	month 3	61.4	3.18	2.6
9092	Dex	month 4	61	2.83	2.9
9092	Dex	month 5	60	3.64	2.85
9092	Dex	month 6	53.8	3.35	2.87
9101	Con	month 1	34.6	3.94	3.5
9101	Con	month 2	41.1	4.49	2.72
9101	Con	month 3	37.3	2.3	2.82
9101	Con	month 4	32.6	5.59	3.13
9101	Con	month 5	33.7	3.57	3.15
9101	Con	month 6	28.1	4.65	3.25
9112	Con	month 1	55	2.97	2.9
9112	Con	month 2	62.4	2.53	2.43
9112	Con	month 3	54	1.56	2.68
9112	Con	month 4	57.6	3.1	2.78
9112	Con	month 5	52	3.37	2.76
9112	Con	month 6	48	3.95	2.86
9123	Ins+Dex	month 1	31.4	3.88	3.7
9123	Ins+Dex	month 2	39.5	3.17	2.96
9123	Ins+Dex	month 3	40.4	3.17	3.01
9123	Ins+Dex	month 4	32.2	4.9	2.95
9123	Ins+Dex	month 5	40.2	3.26	3.1
9123	Ins+Dex	month 6	31.3	4.75	3.13
9128	Con	month 1	27.6	2.86	3.9
9128	Con	month 2	40.7	6.07	3.39
9128	Con	month 3	44.2	2.82	2.59
9128	Con	month 4	44	2.97	2.61
9128	Con	month 5	40.8	3.26	2.59
9128	Con	month 6	40.9	2.75	2.69
9132	Dex	month 1	31.1	3.86	2.63
9132	Dex	month 2	41.5	3.64	2.05
9132	Dex	month 3	39.6	2.7	2.97
9132	Dex	month 4	39.4	3.39	2.85
9132	Dex	month 5	39.6	3.81	2.93
9132	Dex	month 6	38.1	4.25	3.14
9141	Con	month 1	44.4	3.45	3.02
9141	Con	month 2	59.6	2.6	2.48
9141	Con	month 3	52	0.99	2.86
9141	Con	month 4	43.8	2.48	3.21
9141	Con	month 5	48.4	1.92	2.96
9141	Con	month 6	44	2.59	2.79
9141	Dex	month 1	26.5	6.41	3.8
9141	Dex	month 2	39.5	3.64	2.81
9141	Dex	month 3	43.2	4.58	3.04
9141	Dex	month 4	35.7	3.62	3.12
9141	Dex	month 5	38.2	4.08	3.15
9141	Dex	month 6	39.1	4.23	3.31

Cow	group	Time	Milk Kg	Fat %	Protein %		
9147	Ins+Dex	month 1	26.4	5.7	4.25		
9147	Ins+Dex	month 2	50.4	3.42	2.67		
9147	Ins+Dex	month 3	60.4	3.91	2.81		
9147	Ins+Dex	month 4	64	2.91	2.89		
9147	Ins+Dex	month 5	60.2	3.77	2.76		
9147	Ins+Dex	month 6	49.6	2.08	3.05		
9160	Ins+Dex	month 1	44	4.65	3.28		
9160	Ins+Dex	month 2	46.4	2.86	2.87		
9160	Ins+Dex	month 3	49.2	3.99	2.98		
9160	Ins+Dex	month 4	.	.	.		
9160	Ins+Dex	month 5	.	.	.		
9160	Ins+Dex	month 6	.	.	.		
9166	Con	month 1	32.4	4.26	4.1		
9166	Con	month 2	49	3.89	2.75		
9166	Con	month 3	45.4	1.73	2.83		
9166	Con	month 4	38.8	1.39	2.95		
9166	Con	month 5	.	.	.		
9166	Con	month 6	.	.	.		
9167	Ins	month 1	50.2	3.23	2.66		
9167	Ins	month 2	47	3.41	2.9		
9167	Ins	month 3	39.6	3.75	2.8		
9167	Ins	month 4	26.4	2.05	3.15		
9167	Ins	month 5	.	.	.		
9167	Ins	month 6	.	.	.		
9168	Con	month 1	52.4	4.32	2.92		
9168	Con	month 2	57.2	2.79	2.68		
9168	Con	month 3	52.6	2.56	2.89		
9168	Con	month 4	47.8	3.54	3.27		
9168	Con	month 5	48.8	4.15	3.13		
9168	Con	month 6	45.4	3.81	2.94		
9178	Con	month 1	43.8	2.28	2.47		
9178	Con	month 2	53.2	1.72	2.5		
9178	Con	month 3	48.2	2.46	2.63		
9178	Con	month 4	44.2	3.29	2.5		
9178	Con	month 5	44.2	3.16	2.54		
9178	Con	month 6	41.2	3.26	2.5		
9178	Ins	month 1	32.2	3.8	2.8		
9178	Ins	month 2	.	.	.		
9178	Ins	month 3	.	.	.		
9178	Ins	month 4	.	.	.		
9178	Ins	month 5	.	.	.		
9178	Ins	month 6	.	.	.		
9181	Ins+Dex	month 1	40.4	5.5	2.74		
9181	Ins+Dex	month 2	45.8	2.35	2.8		
9181	Ins+Dex	month 3	46.2	3.28	2.83		
9181	Ins+Dex	month 4	44.8	3.53	3.06		
9181	Ins+Dex	month 5	42.2	2.93	3.01		
9181	Ins+Dex	month 6	34.4	4.28	2.97		

Cow	group	Time	Milk Kg	Fat %	Protein %
9194	Dex	month 1	32	3.54	2.99
9194	Dex	month 2	36	4.24	3.03
9194	Dex	month 3	36.6	4	3.44
9194	Dex	month 4	34	4.18	3.57
9194	Dex	month 5	30	4.59	3.35
9194	Dex	month 6	30.8	4.78	3.23
9204	Ins+Dex	month 1	40.8	2.41	3.34
9204	Ins+Dex	month 2	.	.	.
9204	Ins+Dex	month 3	.	.	.
9204	Ins+Dex	month 4	.	.	.
9204	Ins+Dex	month 5	.	.	.
9204	Ins+Dex	month 6	.	.	.
9210	Dex	month 1	23	2.72	4.31
9210	Dex	month 2	43.6	4.43	3.15
9210	Dex	month 3	40.8	3.53	2.85
9210	Dex	month 4	37.4	3.25	3.13
9210	Dex	month 5	35.6	4.12	3.24
9210	Dex	month 6	31.4	4.81	3.24
9211	Con	month 1	30	4.3	4.37
9211	Con	month 2	36	4.12	2.89
9211	Con	month 3	42	3.61	2.95
9211	Con	month 4	34	3.55	3.06
9211	Con	month 5	32.2	5.81	3.18
9211	Con	month 6	32.2	4.14	3.11
9218	Dex	month 1	30.6	4.8	3.49
9218	Dex	month 2	40.2	3.27	3.01
9218	Dex	month 3	36.4	3.01	3
9218	Dex	month 4	34.4	3.47	3.39
9218	Dex	month 5	38	3.19	3.3
9218	Dex	month 6	.	.	.
9226	Con	month 1	33.6	1.88	2.48
9226	Con	month 2	38.1	3.4	2.79
9226	Con	month 3	41.4	2.21	2.87
9226	Con	month 4	38.9	3.62	2.99
9226	Con	month 5	37.9	3	3.17
9226	Con	month 6	37.9	3.34	3.08
9228	Ins+Dex	month 1	48.4	3.48	2.88
9228	Ins+Dex	month 2	53	2.84	2.98
9228	Ins+Dex	month 3	45.2	1.52	3.28
9228	Ins+Dex	month 4	47	4.58	3.13
9228	Ins+Dex	month 5	44.6	2.89	2.98
9228	Ins+Dex	month 6	39	3.45	3.05
9229	Dex	month 1	39.4	3.79	2.86
9229	Dex	month 2	52	2.91	2.74
9229	Dex	month 3	57.6	2.37	2.63
9229	Dex	month 4	55.4	3.48	2.74
9229	Dex	month 5	51.8	3.45	2.68
9229	Dex	month 6	51.4	2.86	2.62

Cow	group	Time	Milk Kg	Fat %	Protein %		
9290	Ins+Dex	month 1	43.4	3.72	2.98		
9290	Ins+Dex	month 2	52	3.63	2.56		
9290	Ins+Dex	month 3	51.4	2.89	2.65		
9290	Ins+Dex	month 4	49.6	2.78	2.92		
9290	Ins+Dex	month 5	23.8	3.33	3.62		
9290	Ins+Dex	month 6	25.8	2.65	2.93		
9313	Con	month 1	37	4.8	3.58		
9313	Con	month 2	50.6	3.21	2.85		
9313	Con	month 3	44	3.17	2.88		
9313	Con	month 4	46.6	4.14	3.08		
9313	Con	month 5	38.4	4.63	3.27		
9313	Con	month 6	42.4	3.43	3.08		
9341	Ins	month 1	38.8	3.03	3.16		
9341	Ins	month 2	56.8	3.66	2.56		
9341	Ins	month 3	55.4	2.79	2.67		
9341	Ins	month 4	54.4	3.07	2.81		
9341	Ins	month 5	49.8	3.95	2.9		
9341	Ins	month 6	48	3.29	2.83		
9352	Ins	month 1	39.6	3.3	3.17		
9352	Ins	month 2	47	2.91	2.76		
9352	Ins	month 3	39.4	4.7	3.12		
9352	Ins	month 4	46	2.42	3		
9352	Ins	month 5	47.2	1.89	3		
9352	Ins	month 6	46	2.4	2.84		
9358	Con	month 1	36.8	5.31	2.79		
9358	Con	month 2	38.4	4.75	2.61		
9358	Con	month 3	44.2	2.63	2.7		
9358	Con	month 4	42.4	3.17	2.78		
9358	Con	month 5	30.4	3.89	2.83		
9358	Con	month 6	21.2	2.8	2.83		
9359	Ins+Dex	month 1	35.2	3.53	2.88		
9359	Ins+Dex	month 2	42	3.97	2.65		
9359	Ins+Dex	month 3	40.4	2.92	2.83		
9359	Ins+Dex	month 4	37.4	4.03	3.12		
9359	Ins+Dex	month 5	37.8	3.31	2.82		
9359	Ins+Dex	month 6	36.4	3.05	2.8		
9365	Ins+Dex	month 1	42.4	3.8	3.14		
9365	Ins+Dex	month 2	50.4	2.99	2.76		
9365	Ins+Dex	month 3	50.8	2.8	2.97		
9365	Ins+Dex	month 4	46.4	3.72	3.09		
9365	Ins+Dex	month 5	44.4	2.91	3.09		
9365	Ins+Dex	month 6	39.8	3.57	3.13		
9375	Ins+Dex	month 1	43.2	2.78	2.26		
9375	Ins+Dex	month 2	9	7.41	3.42		
9375	Ins+Dex	month 3	33.8	1.28	2.63		
9375	Ins+Dex	month 4	39.8	1.92	2.61		
9375	Ins+Dex	month 5	40	3.26	2.45		
9375	Ins+Dex	month 6	36	2.18	2.52		

Cow	group	Time	Milk Kg	Fat %	Protein %
9377	Con	month 1	28	2.7	3.69
9377	Con	month 2	49	4.28	2.61
9377	Con	month 3	36.4	2.9	2.49
9377	Con	month 4	42.6	2.49	2.94
9377	Con	month 5	46	3.12	2.92
9377	Con	month 6	44.8	3.54	2.67
9435	Con	month 1	41.4	2.38	2.46
9435	Con	month 2	41.5	2.42	2.47
9435	Con	month 3	42.2	2.59	2.33
9435	Con	month 4	40.6	1.73	2.34
9435	Con	month 5	30	.	.
9435	Con	month 6	41.1	1.67	2.75
9587	Dex	month 1	33.8	1.93	2.63
9587	Dex	month 2	32.6	2.21	2.45
9587	Dex	month 3	35.4	2.28	2.58
9587	Dex	month 4	40	2.7	2.68
9587	Dex	month 5	39	3.35	2.63
9587	Dex	month 6	40	3.04	2.77
9594	Ins+Dex	month 1	32.2	2.89	2.46
9594	Ins+Dex	month 2	33.2	1.92	2.83
9594	Ins+Dex	month 3	40.2	3.5	3.01
9594	Ins+Dex	month 4	44	3.56	2.84
9594	Ins+Dex	month 5	.	.	.
9594	Ins+Dex	month 6	.	.	.
9929	Ins	month 1	40.5	4.03	2.94
9929	Ins	month 2	40.8	4.39	1.9
9929	Ins	month 3	45.3	5.88	2.7
9929	Ins	month 4	44.6	3.1	2.61
9929	Ins	month 5	42	3.81	2.7
9929	Ins	month 6	34.2	3.1	3.25
9948	Dex	month 1	30.6	4.25	3.43
9948	Dex	month 2	38.8	3.66	2.6
9948	Dex	month 3	36.9	4.15	2.68
9948	Dex	month 4	34.8	3.37	2.95
9948	Dex	month 5	38.7	3.77	2.99
9948	Dex	month 6	33.3	4.38	3.19
51008	Ins+Dex	month 1	27.6	3.48	4.37
51008	Ins+Dex	month 2	50.4	3.78	2.78
51008	Ins+Dex	month 3	42.8	4.94	2.9
51008	Ins+Dex	month 4	43	5.14	2.92
51008	Ins+Dex	month 5	.	.	.
51008	Ins+Dex	month 6	.	.	.
51108	Con	month 1	40.9	5.05	2.99
51108	Con	month 2	27	5.19	2.94
51108	Con	month 3	34.6	2.91	3.03
51108	Con	month 4	30.8	4.85	3.21
51108	Con	month 5	12.8	8.22	3.34
51108	Con	month 6	26.8	4.22	3.19

Cow	group	Time	Milk Kg	Fat %	Protein %		
55112	Dex	month 1	33.1	2.02	2.46		
55112	Dex	month 2	.	.	.		
55112	Dex	month 3	.	.	.		
55112	Dex	month 4	.	.	.		
55112	Dex	month 5	.	.	.		
55112	Dex	month 6	.	.	.		
