# Supplementary material for: Effects of Dexamethasone and Insulin Alone or in Combination on Energy and Protein Metabolism Indicators and Milk Production in Dairy Cows in Early Lactation – A Randomized Controlled Trial
Source: PLoS One. 2015 Sep 30;10(9):e0139276. doi: 10.1371/journal.pone.0139276 (PMC4589323; doi:10.1371/journal.pone.0139276)
Supplement: S2 Table — (DOC) [file pone.0139276.s002.doc]

| 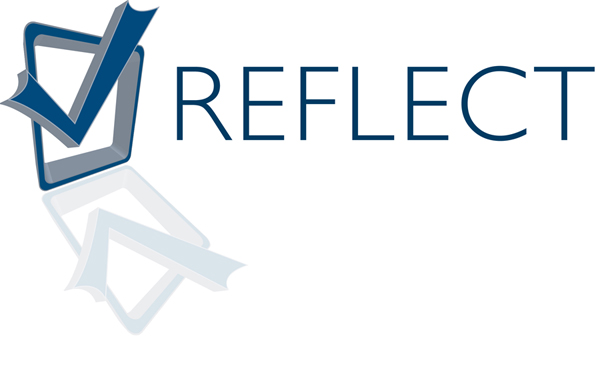 | Checklist for REFLECT statement: Reporting guidelines For randomized control trials in livestock and food safety. Bold text are modifications from the CONSORT statement description (Altman DG et al . Ann Intern Med 2001; 134(8):663-694). | | |
| --- | --- | --- | --- |
| **Paper section and topic** | **Item** | **Descriptor of REFLECT statement item** | **Reported on Page #** |
| Title & Abstract | 1 | How **study units** were allocated to interventions ( eg, "random allocation," "randomized," or "randomly assigned"). **Clearly state whether the outcome was the result of natural exposure or was the result of a deliberate agent challenge.** | 5 |
| Introduction Background | 2 | Scientific background and explanation of rationale. | 3-4 |
| Methods Participants | 3 | Eligibility criteria **for owner/managers and study units at each level of the organizational structure,** and the settings and locations where the data were collected. | 5 |
| Interventions | 4 | Precise details of the interventions intended for each group, **the level at which the intervention was allocated,** and how and when interventions were actually administered. | 5-6 |
|  | 4b | **Precise details of the agent and the challenge model, if a challenge study design was used.** | 5-6 |
| Objectives | 5 | Specific objectives and hypotheses. **Clearly state primary and secondary objectives (if applicable).** | 4 |
| Outcomes | 6 | Clearly defined primary and secondary outcome measures and the levels at which they were measured, and, when applicable, any methods used to enhance the quality of measurements (eg, multiple observations, training of assessors). | 8 |
| Sample size | 7 | How sample size was determined and, when applicable, explanation of any interim analyses and stopping rules. **Sample-size considerations should include sample-size determinations at each level of the organizational structure and the assumptions used to account for any non-independence among groups or individuals within a group.** | 5 |
| Randomization --Sequence generation | 8 | Method used to generate the random allocation sequence **at the relevant level of the organizational structure**, including details of any restrictions (eg, blocking, stratification) | 5 |
| Randomization --Allocation concealment | 9 | Method used to implement the random allocation sequence **at the relevant level of the organizational structure**, (eg, numbered containers **~~or central telephone~~),** clarifying whether the sequence was concealed until interventions were assigned. | 5 |
|  |  | | |
| Randomization --Implementation | 10 | Who generated the allocation sequence, who enrolled **study units,** and who assigned **study units** to their groups **at the relevant level of the organizational structure**. | 5-6 |
| Blinding (masking) | 11 | Whether or not **~~participants~~** those administering the interventions, **caregivers** and those assessing the outcomes were blinded to group assignment. If done, how the success of blinding was evaluated**. Provide justification for not using blinding if it was not used.** | 5 |
| Statistical methods | 12 | Statistical methods used to compare groups for all outcome(s); Clearly state the level of statistical analysis **and methods used to account for the organizational structure, where applicable;** methods for additional analyses, such as subgroup analyses and adjusted analyses. | 9-10 |
| Results  **Study** flow | 13 | Flow **of study units** through each stage **for each level of the** **organization structure of the study** (a diagram is strongly recommended). Specifically, for each group, report the numbers of **study units** randomly assigned, receiving intended treatment, completing the study protocol, and analyzed for the primary outcome. Describe protocol deviations from study as planned, together with reasons. | 10-14 |
| Recruitment | 14 | Dates defining the periods of recruitment and follow-up. | 5-6 |
| Baseline data | 15 | Baseline demographic and clinical characteristics of each group, **explicitly providing information for each relevant level of the organizational structure. Data should be reported in such a way that secondary analysis, such as risk assessment, is possible.** | 6 and 9 |
| Numbers analyzed | 16 | Number **of study units** (denominator) in each group included in each analysis and whether the analysis was by "intention-to-treat." State the results in absolute numbers when feasible (eg, 10/20, not 50%). | 11-13 |
| Outcomes and estimation | 17 | For each primary and secondary outcome, a summary of results for each group, **accounting for each relevant level of the organizational structure**, and the estimated effect size and its precision (e.g., 95% confidence interval) | 12-14 |
| Ancillary analyses | 18 | Address multiplicity by reporting any other analyses performed, including subgroup analyses and adjusted analyses, indicating those pre-specified and those exploratory. | 10-11 |
| Adverse events | 19 | All important adverse events or side effects in each intervention group. | 10 |
| Discussion Interpretation | 20 | Interpretation of the results, taking into account study hypotheses, sources of potential bias or imprecision, and the dangers associated with multiplicity of analyses and outcomes. **Where relevant, a discussion of herd immunity should be included. If applicable, a discussion of the relevance of the disease challenge should be included.** | 14-19 |
| Generalizability | 21 | Generalizability (external validity) of the trial findings. | 19 |
| Overall evidence | 22 | General interpretation of the results in the context of current evidence. | 19 |
